# The Emotional Experience of Time: Effects of Anxiety and Interoceptive Ability on Temporal Perception

**DOI:** 10.1002/hbm.70650

**Published:** 2026-09-24

**Authors:** Gaia Lapomarda, Alessio Fracasso, Carmen Morawetz, Alessandro Grecucci, David Melcher

**Affiliations:** ^1^ Department of Psychology University of Innsbruck Innsbruck Austria; ^2^ Department of General Psychology University of Padua Padua Italy; ^3^ Department of Education, Psychology and Communication Sciences University of Bari Bari Italy; ^4^ Division of Science New York University Abu Dhabi Abu Dhabi UAE; ^5^ Center for Brain & Health New York University Abu Dhabi Abu Dhabi UAE

**Keywords:** anxiety, interoception, Threat of Scream, time perception

## Abstract

Our experience of time is deeply shaped by emotional and bodily states. Anxiety is known to distort our temporal perception, yet the underlying mechanisms remain poorly understood. Here, we tested whether interoception links anxiety to variability in timing. Thirty participants underwent fMRI while performing a temporal reproduction task within a modified Threat of Scream paradigm. Safe and Threat blocks alternated, with aversive screams unpredictably delivered during Threat blocks. Screams reliably increased subjective anxiety and engaged salience and limbic regions, including the amygdala, hippocampus, and insula. Despite these affective and neural responses, Threat did not produce systematic changes in temporal accuracy. Instead, individuals with higher interoceptive accuracy tended to preserve reproduction accuracy under elevated anxiety, implicating an interoceptive contribution to anxiety‐related variability in timing. At the neural level, reproduction compared with the encoding phase increased activity in sensorimotor and parietal regions, with engagement of the insula, cingulate, and striato‐thalamic regions in the Safe context. Under Threat, the same contrast encompassed a more circumscribed set of sensorimotor and parietal regions, and screams robustly recruited a salience–limbic–temporal network relative to both non‐emotional tones. Together, these findings suggest that individual differences in interoceptive accuracy may moderate the relationship between anxiety and temporal reproduction, and characterize the neural response to threat during a timing task.

## Key Points

- Threat increased subjective anxiety but did not systematically alter temporal reproduction accuracy.
- Individual differences in interoception relate to how strongly anxiety impacts timing.
- Scream events engaged salience and limbic networks, and encoding and reproduction phases showed slightly different activation patterns under Safe and Threat.

## Graphical abstract

Does the body shape how anxiety warps time? During fMRI, the threat of unpredictable screams engaged salience–limbic circuits but did not uniformly distort timing. Instead, individuals with higher interoceptive accuracy preserved temporal reproduction under anxiety, suggesting bodily awareness as a moderator linking anxiety and subjective time.

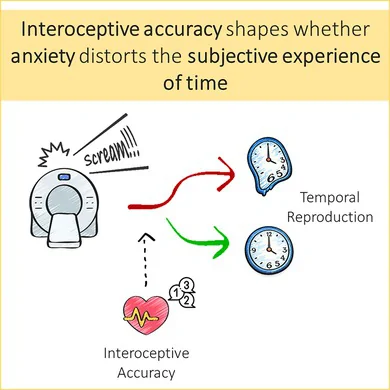

## 1 Introduction

A coherent sense of time is essential for human cognition. It allows us to coordinate actions, plan effectively, and adapt flexibly to an ever‐changing environment. When this sense is disrupted, the continuity of daily life becomes disorganized. Events may feel disconnected, tasks may be difficult to structure, and social interactions may become challenging to maintain. Such disturbances in temporal perception are commonly seen in various psychiatric and neurological conditions. For instance, individuals with depression often report a slowing of time, while those with anxiety may experience time as accelerated (Mioni et al. 2016). Disruptions of time are also a characteristic of ADHD (Ptacek et al. 2019), and schizophrenia is associated with a fragmented sense of temporal flow (Thoenes and Oberfeld 2017). These alterations underscore the relevance of studying time perception, both as a window into fundamental cognitive processes and as a potential transdiagnostic mechanism in mental health.

Emotions exert a powerful influence on how we experience time. Threatening or unpleasant events are typically perceived as lasting too long, whereas enjoyable or engaging moments feel like time passes too quickly (Droit‐Volet and Gil 2009; Droit‐Volet and Meck 2007; Wittmann 2009). Classical accounts of time perception, such as the internal clock theory (Gibbon 1977) and the attentional gate model (Block and Zakay 1997), conceptualize interval timing as a process of accumulating pulses akin to a pacemaker filtered through attentional mechanisms. While influential, these models fall short of explaining why emotional states so powerfully distort temporal judgments, pointing to the need for models that integrate affective dimensions into theories of time perception.

An increasing amount of evidence suggests that interoceptive signals play a key role in our perception of time. According to the embodiment framework, the insula and associated prefrontal regions integrate ascending visceral rhythms, such as cardiac signals, into a unified sense of time (Arslanova et al. 2023; Craig 2002, 2004, 2009). Consistent with this view, activity in the posterior and anterior insula builds up across several‐second intervals during temporal reproduction, which is compatible with an accumulator‐like coding of time (Vicario et al. 2020; Wittmann et al. 2010). Psychophysiological studies further demonstrate that duration reproduction accuracy correlates with both autonomic changes during interval encoding and individual differences in heartbeat perception, supporting a contribution of interoceptive processing to supra‐second timing (Meissner and Wittmann 2011; Otten et al. 2015; Pollatos et al. 2014). More broadly, research indicates that temporal experience is “embodied” in ongoing bodily rhythms and their cortical representation (Di Lernia et al. 2018; Uraguchi et al. 2022; Volodina et al. 2025).

At the same time, the direction of the interoceptive influence on the emotion–time relationship is not straightforward. Individuals with higher cardiac interoceptive accuracy show stronger autonomic and subjective responses to emotional stimuli (Pollatos, Gramann, and Schandry 2007; Pollatos, Herbert, et al. 2007; Ventura‐Bort et al. 2021), suggesting that enhanced bodily awareness may amplify affective states. In contrast, people with higher interoceptive accuracy can be less susceptible to emotion‐driven temporal distortions (Özoğlu and Thomaschke 2020), pointing to a potential protective role of interoceptive accuracy against some temporal distortions. These findings suggest that interoceptive ability does not uniformly increase or decrease temporal distortions, but rather modulates how strongly momentary anxiety states affect temporal perception (Tabor et al. 2019).

This proposal aligns with models of the salience network, in which the anterior insula and dorsal anterior cingulate cortex (ACC) integrate bodily signals and external sensory information to prioritize salient events. Once such events are detected, this network helps shift the brain into an appropriate processing mode, for example, by disengaging from ongoing activity and engaging task‐focused control networks under threat (Menon and Uddin 2010; Seeley et al. 2007; Seeley 2019). Anxiety can be conceptualized as a state in which threat‐related and bodily signals receive heightened weighting in this network, often accompanied by increased engagement of the amygdala and related limbic structures. Threat‐of‐shock paradigms are a well‐established tool to induce such anticipatory anxiety in the scanner, as they reliably elevate subjective anxiety and recruit amygdala–insula–ACC circuits as a function of anxiety ratings (Balderston et al. 2017; Bijsterbosch et al. 2015; Carlson et al. 2010; Drabant et al. 2011; Vytal et al. 2014). Building on these works, the Threat of Scream (ToS) paradigm has recently been introduced as a non‐invasive alternative, in which electric shocks are replaced by unpredictable human distress screams delivered during “Threat” blocks that alternate with “Safe” blocks in which no screams occur (Beaurenaut et al. 2020). In this design, low‐intensity but aversive screams reliably increase subjective anxiety and autonomic activity (skin conductance) in the Threat relative to Safe block, demonstrating that unpredictable aversive screams can induce sustained anxiety (Beaurenaut et al. 2020). Yet, few studies combined an experimental manipulation of anxiety with objective measures of interoceptive accuracy and neuroimaging during a temporal reproduction task (Lake et al. 2016; Vicario et al. 2020; Volodina et al. 2025). Consequently, it remains unclear whether, and under what conditions, threat‐related anxiety alters temporal perception, and whether individual differences in interoceptive processing influence this relationship. Because the present design measures interoception offline (that is, before the MRI task), it is suited to testing whether interoception moderates the effect of threat on timing, rather than to identifying a specific underlying timing mechanism.

The present study addresses this gap by combining anxiety manipulation (alternating Safe and Threat blocks with unpredictable aversive screams) with a temporal reproduction task, interoceptive assessment, and functional magnetic resonance imaging (fMRI). In particular, we aimed to test whether threat‐induced anxiety alters temporal estimation through its interaction with interoception. Given that interoceptive abilities differ in each individual, this framework allowed us to additionally test the hypothesis that anxiety effects may emerge in specific interoceptive profiles. On a behavioral level, we first predicted that the threat of unpredictable screams would increase subjective anxiety compared to safe contexts. Secondly, we tested whether subjective anxiety impairs temporal reproduction performance and whether interoception moderates this relationship. Specifically, we predicted that individuals with greater interoceptive accuracy would display stronger subjective anxiety responses to threat. Regarding temporal reproduction, we tested whether interoceptive accuracy amplified or buffered anxiety‐related effects on temporal accuracy, without specifying a direction, given mixed evidence in previous research. At the neural level, we expected that scream delivery would selectively recruit salience and limbic circuits (such as amygdala, insula, anterior cingulate), confirming the sensitivity and specificity of the manipulation. Finally, we further explored how encoding versus reproduction phases of time perception may differently recruit cortical and subcortical systems under safe and threatening conditions, with a particular emphasis on the amygdala and insula as core hubs of threat detection and interoceptive integration.

## 2 Materials and Methods

### 2.1 Participants

To inform the final design of the fMRI experiment, we first ran a behavioral pilot (*N* = 45, 19–23 years). Interoceptive accuracy, interoceptive sensibility, and trait anxiety were not assessed in the pilot. However, post‐block subjective anxiety ratings were collected. The pilot served to validate the temporal reproduction task and the threat manipulation and to select the target durations for the fMRI task (see Section 2.4). In particular, the pilot showed that the sub‐second interval (0.6 s) could not be reliably reproduced, as participants reported difficulties in detecting it. Also, studies using 2‐second intervals struggled to show how neural activity develops over time, while those with longer intervals demonstrated how the perception of duration evolves (Wittmann et al. 2010). So, we decided to use only supra‐second intervals (i.e., 4‐, 9‐, and 14‐s) in the scanner.

For the fMRI experiment, a sample of 30 healthy volunteers (15 females, mean age = 21.75 ± 4.28) was recruited at New York University Abu Dhabi (NYUAD) and received either course credit or monetary compensation. All participants signed and provided informed consent and a data protection agreement. Inclusion criteria included normal or corrected‐to‐normal vision, no hearing impairments, no metallic foreign bodies (e.g., metal plates, pins, or bolts in the head, cardiac pacemaker, any magnetic implants), and not indicating any risk factor on the NYUAD MRI screening questionnaire administered before participation. They also did not have any history of brain injury, brain surgery, general cognitive impairment, or a history of neurological disorders. Data were collected in accordance with the Declaration of Helsinki, and the study protocol was approved by the local ethics committee (HRPP‐2022‐140).

### 2.2 Questionnaires

One week before the MRI scan, participants anonymously completed two self‐report online questionnaires: the State‐Trait Anxiety Inventory (STAI) (Spielberger et al. 1970) and the Multidimensional Assessment of Interoceptive Awareness (MAIA) (Mehling et al. 2012, 2018).

The STAI is a widely used self‐report instrument designed to measure State (Form Y‐1) and Trait (Form Y‐2) anxiety. State anxiety is a temporary emotional state marked by feelings of tension and nervousness, reflecting how someone feels “right now.” Trait anxiety refers to stable individual differences in anxiety levels, indicating a person's general tendency to perceive situations as threatening and feel anxious. Each scale consists of 20 items, yielding separate total scores for state and trait anxiety (range 20–80). Higher scores indicate greater anxiety.

The MAIA is a self‐report questionnaire to assess multiple dimensions of interoceptive sensibility—the self‐perceived tendency to notice and interpret internal bodily sensations (Garfinkel et al. 2015). It consists of 32 items, rated on a 6‐point Likert scale (0 = *Never* to 5 = *Always*), and covers eight subscales: Noticing (the awareness of comfortable, uncomfortable, and neutral body sensations); Not‐Distracting (the tendency not to ignore or distract oneself from sensations of pain or discomfort); Not‐Worrying (the tendency not to experience emotional distress or worry with sensations of pain or discomfort); Attention Regulation (the ability to sustain and control attention to body sensations); Emotional Awareness (the awareness of the connection between bodily sensations and emotional states); Self‐Regulation (the ability to regulate distress by attention to body sensations); Body Listening (the active listening to the body for insight); Trusting (the experience of the body as safe and trustworthy). Higher scores indicate greater interoceptive sensibility in each dimension.

### 2.3 Heartbeat Tracking Task

Interoceptive accuracy was assessed outside the scanner using the Heartbeat Tracking (HBT) task (Garfinkel et al. 2015; Schandry 1981). Participants silently counted their heartbeats during different periods (e.g., 25, 35, 45 s) without manually checking, from the time they heard “START” to when they heard “STOP.” To objectively measure participants' cardiac signals, their actual heartbeats were continuously recorded (beats*real*) with a Polar H10 chest strap (Polar Electro, Kempele, Finland), which uses electrocardiography technology to detect R‐peaks (Schaffarczyk et al., Schaffarczyk et al. 2022). The test was performed six times to form six trials, using time windows of 25, 30, 35, 40, 45, and 50 s, presented in randomized order. An interoceptive accuracy index (IAcc) for each participant was derived (Garfinkel et al. 2015; Schandry 1981):

$$\text{IAcc}=\frac{1}{6}\times \Sigma \times \left(1-|\left.n\;\text{beats}\text{real}-n\;\text{beats}\text{reported}\right.|/n\;\text{beats}\text{real}\right)\;$$

Seven participants out of 30 performed 5 trials using time windows of 25, 30, 35, 40, and 45 s, due to an error in the measurements with the Polar chest strap.

After hearing “STOP,” participants were asked to report the number of heartbeats mentally counted (beats*reported*).

### 2.4 Experimental Procedure

Before coming to the lab, participants completed trait anxiety and interoceptive sensibility questionnaires online. On the day of scanning, they performed the interoceptive accuracy task before entering the scanner. After informed consent, participants completed a brief training session outside the scanner to familiarize themselves with the task. They were instructed not to count or tap during timing and to reproduce tone durations as accurately as possible.

**FIGURE 1:**
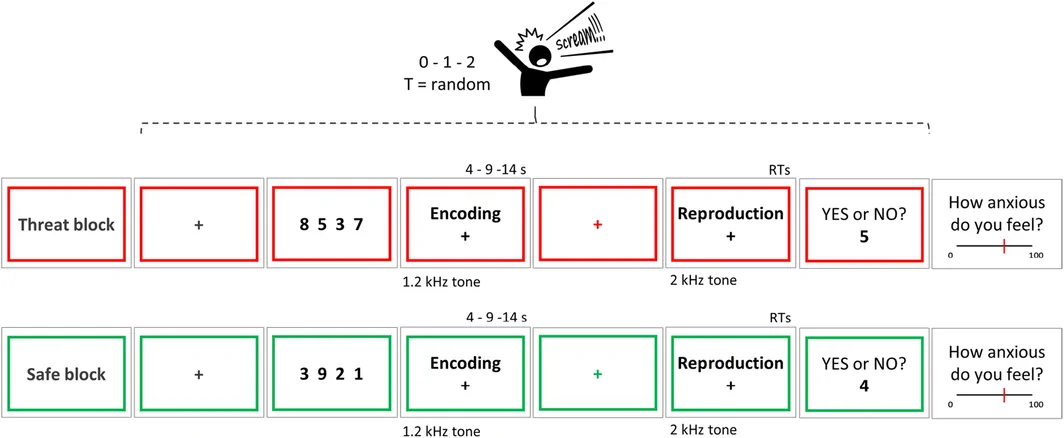
Task overview. During fMRI, participants performed a temporal reproduction task under alternating blocks of Safe (green) and Threat (red). On each trial, they heard a tone of varying durations (Encoding: 1.2 kHz; 4/9/14 s) and then reproduced its duration by stopping a subsequent tone (Reproduction: 2 kHz). In Threat blocks, unpredictable human screams occurred on ~10% of trials (never in Safe), inducing acute anxiety while the timing task was ongoing. A brief memory task (4‐digit set and probe) discouraged counting. Subjective anxiety was rated after each block on a scale from 0 to 100. Trait anxiety, interoceptive sensibility, and interoceptive accuracy were assessed outside the scanner.

During the fMRI session, participants were presented with a novel Anxiety Temporal Reproduction Task in the scanner, which combined a “Threat of Scream” (ToS) manipulation (Beaurenaut et al. 2020) with a temporal reproduction task (Wittmann et al. 2010). They completed 12 blocks, alternating 6 Threat (red side bars = risk of scream) and 6 Safe (green side bars = no screams). During both blocks, they performed a temporal reproduction task with three target durations (tones: 4–9–14 s) and a secondary memory task to discourage counting (Figure 1). In the pilot (6 blocks; 30 trials/block), 0.6–4–9 s were tested. The sub‐second interval proved unreliable to reproduce, so the fMRI task focused on seconds‐range durations (Wittmann et al. 2010). Each trial followed the same sequence: (i) four‐digit memory set to memorize and maintain until the end of the trial; (ii) delivery of encoding tone (T1; 1.2 kHz; 4–9–14 s); (iii) brief fixation cross; (iv) delivery of reproduction tone (T2; 2 kHz) terminated by button press when participants judged it reached the same duration as T1; (v) memory probe (single digit) to indicate via button press (yes/no) whether it belonged to the initial four‐digit set. In Threat blocks only, six human screams (3 females, 3 males; normalized to −2 dB) (Beaurenaut et al. 2020; Fecteau et al. 2005) occurred unpredictably on ~10% of trials randomly at one of three windows (before memory set; after memory set/before T1; after T2/before probe). All sounds (i.e., tones, screams) were presented binaurally via MRI‐compatible headphones. After each block, participants rated subjective anxiety (0–100).

### 2.5 Behavioral Analysis

Statistical analyses were performed in R (https://www.r‐project.org/) using the *lme4* package to test linear mixed‐effects models (LMMs). Random slopes by subject were included if supported by a likelihood‐ratio test, and the resulting model was non‐singular and convergent. If not, a random‐intercept model was applied (Matuschek et al. 2017).

#### 2.5.1 Sensitivity Analysis

Because the pilot did not include interoceptive measures, we did not run an a priori power analysis for the individual‐difference effects. We therefore conducted a simulation‐based sensitivity analysis using the *simr* package in R (Green and MacLeod 2016) to determine the smallest subjective‐anxiety × interoceptive accuracy interaction detectable in our design. Holding the sample (*N* = 30), trial structure, and random‐effects structure of the fitted model, we simulated 1000 datasets at each of a range of interaction effect sizes and computed the proportion that produced a significant effect (*α* = 0.05).

#### 2.5.2 Manipulation Check

LMM tested the effect of the condition (Safe, Threat) on subjective anxiety ratings (pilot and main). Random intercepts for participants and random slopes for condition were included.

#### 2.5.3 Temporal Reproduction Task

LMMs tested the effects of target duration (pilot: 0.6–4–9 s; main: 4–9–14 s) and condition (Safe, Threat) on reproduced durations. Random intercepts for participants and random slopes for target durations were included.

Only trials with a correct response on the memory probe were included (attentional check). An accuracy of reproduction index was computed per target duration and condition (Teghil, Boccia, et al. 2020; Teghil, Di Vita, et al. 2020):

$$\mathrm{AR}=1-|\left(\text{Target duration}-\text{Reproduced duration}\right)/\text{Target duration}|$$

and modeled with target durations, conditions, and their interaction.

#### 2.5.4 Effect of Trait Anxiety on Subjective Anxiety

LMM tested the effect of condition (Safe, Threat), trait anxiety, and their interaction on subjective anxiety ratings. Random intercepts for participants and random slopes for condition were included.

#### 2.5.5 Effect of Interoception on Subjective Anxiety

LMM tested the effects of condition (Safe, Threat), interoceptive accuracy (IAcc), and their interaction on subjective anxiety ratings. LMM also tested the effects of condition, interoceptive sensibility (MAIA subscales), and their interactions on subjective anxiety. Random intercepts for participants and random slopes for condition were included.

#### 2.5.6 Effect of Subjective Anxiety and Interoception on Accuracy of Reproduction

LMM tested the effects of temporal duration (pilot and main), subjective anxiety ratings, and their interaction on the accuracy of reproduction (AR). In the main study, an additional LMM tested the effects of temporal duration, subjective anxiety, interoceptive accuracy, and their interaction on AR. LMM also tested the effects of condition, interoceptive sensibility (MAIA subscales), and their interactions on AR. Random intercepts for participants and random slopes for target durations were included.

### 2.6 fMRI Data Acquisition

Whole‐brain functional and anatomical images were acquired using a Siemens Prisma 3T full‐body MRI scanner (Siemens Medical Solutions) using a 64‐channel head coil at the New York University Abu Dhabi (NYUAD). For each observer, a T1‐weighted anatomical scan was acquired (TR = 2400 ms; TE = 2.22 ms; flip angle = 8°; 0.8 mm isotropic voxels). This anatomical volume was used for white/gray matter segmentation and coregistration with the functional scans. T2*‐weighted functional scans were acquired using an echoplanar imaging sequence, EPI (TR = 1000 ms; TE = 37 ms; flip angle = 68°; multiband factor = 6; FOV = 104 × 104 mm^2^; 2 × 2 × 2 mm^3^ isotropic voxels; 72 slices).

### 2.7 fMRI Data Processing

Data were organized into the Brain Imaging Data Structure (BIDS) format using *dcm2bids* (Gorgolewski et al. 2017) and *dcm2niix* (Li et al. 2016). Anatomical images underwent preprocessing using the fMRIPrep pipeline version 20.2.3 (Esteban, Blair, et al. 2019; Esteban, Markiewicz, et al. 2019). We processed functional images using *mrtrix* for denoising and AFNI (https://afni.nimh.nih.gov/) (Cox 1996; Cox and Hyde 1997; Veraart et al. 2016).

#### 2.7.1 Anatomical Data Preprocessing

The T1‐weighted (T1w) images were corrected for intensity non‐uniformity (INU) using N4BiasFieldCorrection (Tustison et al. 2014), distributed with ANTs 2.3.3 (Avants et al. 2011), and were used as T1w‐references throughout the workflow. Each T1w‐reference was skull‐stripped with a Nipype implementation of the antsBrainExtraction.sh workflow (from ANTs), using OASIS30ANTs as the target template. Brain tissue segmentation of cerebrospinal fluid (CSF), white matter (WM), and gray matter (GM) was performed on the brain‐extracted T1w images using fast (FSL 5.0.9) (Zhang et al. 2001). Brain surfaces were reconstructed using recon‐all (FreeSurfer 6.0.1) (Dale et al. 1999), and volume‐based spatial normalization to two standard spaces (MNI152NLin2009cAsym, MNI152NLin6Asym) was performed through nonlinear registration with *antsRegistration* (ANTs 2.3.3), using brain‐extracted versions of both the T1w reference and the T1w template.

#### 2.7.2 Functional Data Preprocessing

First, we removed noise from each of the 12 EPI runs using the function *dwidenoise* from *mrtrix3* (https://mrtrix.readthedocs.io/). This is a principal component analysis‐based method that is independent of the study design and has been shown to remove local signal fluctuations originating from thermal noise, while keeping anatomical details unaltered. Next, the 12 EPI runs were slice time corrected using the function *3dTshift* in AFNI. For each fMRI session, a fieldmap was estimated based on two echo‐planar imaging (EPI) references with opposing phase‐encoding directions (anterior‐posterior and posterior‐anterior) using the AFNI function *3dQwarp*, and stored for later use. We computed motion between successive EPI images using the AFNI function *3dVolreg*, aligning EPI images to the EPI with the least amount of motion, determined on the EPI data itself. We combined the fieldmap and the motion estimates in a single field and applied fieldmap correction and motion correction in a single interpolation step to decrease the amount of blurring in the data. We applied a spatial smoothing of 4 mm FWHM Gaussian kernel using the AFNI function *3dMerge*, and converted voxel‐wise signal time courses were scaled to percent signal change. EPI images were then co‐registered to the skull‐stripped T1w reference using the AFNI function *align_epi_anat.py*, we opted for local Pearson correlation (lpc) as cost function, and estimated the best affine transformation (3 rotations, 3 shifts, 3 scaling, 3 shears), projecting the EPI data on the T1w anatomical space.

### 2.8 fMRI Analysis

#### 2.8.1 First‐Level Analysis

Separate general linear models (GLMs) were run for Threat and Safe using the AFNI function 3dDeconvolve within *afni_proc.py*. Stimulus timings were modeled using amplitude‐modulated regressors (AM2) convolved with a dmUBLOCK basis function to accommodate different trial durations. Threat GLMs included three regressors of interest, based on three tone presentation phases (encoding tone, reproduction tone, scream delivery). Safe GLMs included encoding and reproduction tone. Each GLM included nuisance regressors alongside regressors of interest, comprising six motion parameters and fifth‐order polynomial terms to account for low‐frequency signal drifts. The analysis yielded beta coefficients and *t*‐statistics for each condition and contrast of interest. GLM modeling was performed in individual EPI space, then subject‐level statistical maps were projected onto the individual T1w space using the affine transformation described above, and projected on individual cortical surfaces using tools provided by SUMA function *3dVol2Surf*. This was performed separately for left and right hemispheres and for both Safe and Threat conditions.

#### 2.8.2 Region‐of‐Interest (ROI) Based Analysis

To obtain regional estimates of task‐related activation, subject‐level statistical maps (beta and *t*‐statistic volumes) were up‐sampled to the resolution of anatomical atlases using AFNI's *3dresample*. This procedure ensured voxel‐level alignment between functional contrast maps and cortical/subcortical label maps derived from FreeSurfer. Analyses focused on the Destrieux atlas (Destrieux et al. 2010). Voxel‐wise values were imported into R using the *AFNIio* library. For each ROI and each beta coefficient, we computed the 50th percentile of the absolute *t*‐statistic values within the selected ROI. We kept only those betas exceeding the thresholding value and computed the mean beta from the thresholded distribution. We extracted mean beta values across all participants and all ROIs provided in the Destrieux atlas (Destrieux et al. 2010). This resulted in a participant × ROI × regressor dataset for each condition (safe, threat). Details of the specific ROIs can be found in Table S1. To determine whether each regressor elicited significant activation above baseline, we first tested mean beta values for each ROI against zero. Specifically, we fit intercept‐only linear models across participants and examined the significance of the intercept. False discovery rate (FDR) correction was applied across ROIs for each regressor. To adopt a conservative approach and minimize false positives, *p*‐value thresholds were guided by FDR‐corrected results in control regions, specifically the 3rd, 4th, and lateral ventricles, and cerebral and cerebellar WM. Next, to evaluate the effect of our experimental manipulations, we performed ROI‐based repeated‐measures ANOVAs. A 2 × 2 design was used with condition (Safe vs. Threat) and tone presentation phase (encoding vs. reproduction) as within‐subjects factors, to test their main effects and potential interactions. FDR correction was applied across ROIs to control for multiple comparisons. We then examined phase‐related effects (encoding, reproduction) separately within each condition. For Safe and Threat conditions, encoding versus reproduction served as a within‐subjects factor. In the Threat condition, scream delivery was added as an additional factor, and we conducted post‐hoc pairwise comparisons between phases (encoding, reproduction, scream), with FDR correction applied to account for multiple testing.

## 3 Results

### 3.1 Behavioral Pilot Results

The pilot confirmed the manipulation, showing higher subjective anxiety in Threat than Safe (*b* = 18.66, *t*(44) = 6.54, *p* < 0.001). Reproduced durations were significantly shorter at 0.6 s (*b* = −3.01, *t*(58) = –47.80, *p* < 0.001) and longer at 9 s (*b* = 2.75, *t*(47) = 21.01, *p* < 0.001) compared to 4 s. There was no significant main effect of condition on accuracy of reproduction (AR) (*p* = 0.171), nor significant interactions between condition and durations (all *p* ≥ 0.879). A significant effect of subjective anxiety emerged instead (*b* = −0.01, *t*(199) = −2.45, *p* = 0.015), indicating that higher subjective anxiety was associated with lower reproduction accuracy overall, together with a significant interaction between subjective anxiety and the shortest duration (0.6 s: *b* = 0.03, *t*(1018) = 3.42, *p* < 0.001), such that this relationship reversed at 0.6 s, where higher anxiety was associated with greater accuracy.

### 3.2 Behavioral Results

#### 3.2.1 Manipulation Check

**FIGURE 2:**
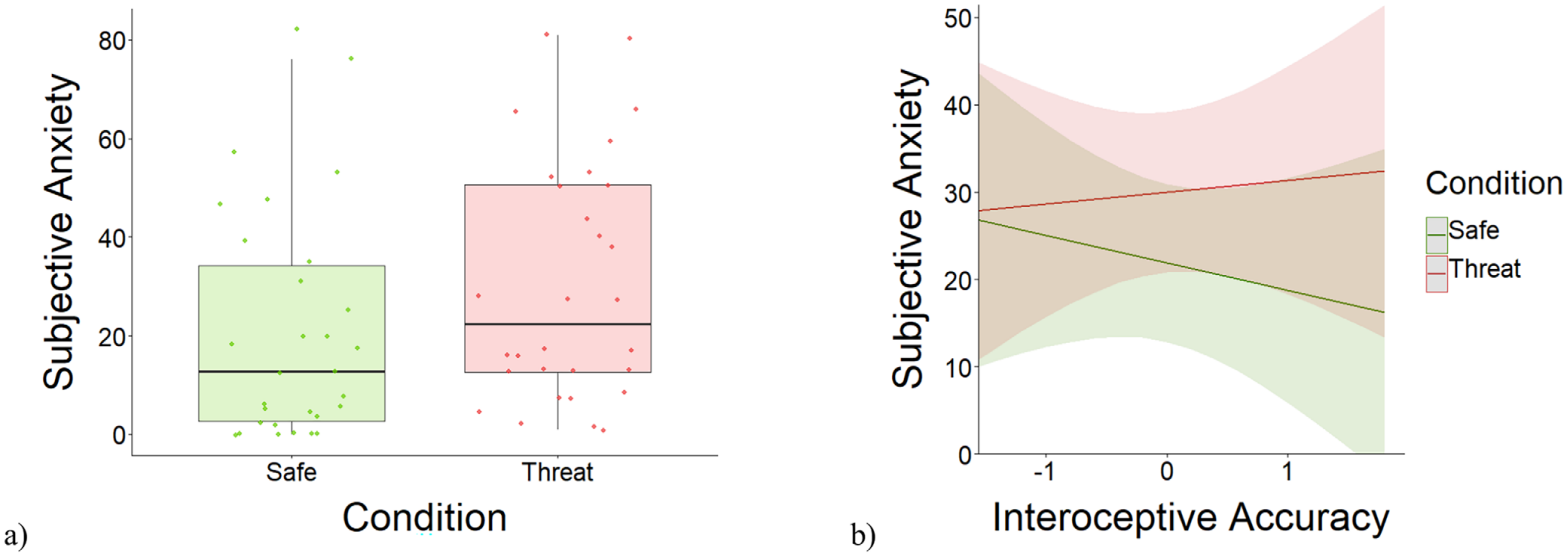
Effects of condition and interoceptive accuracy (IAcc) on subjective anxiety. (a) Main effect of condition, showing higher subjective anxiety scores in the Threat condition compared to the Safe condition (*p* = 0.001). (b) Interaction between condition (Safe vs. Threat) and interoceptive accuracy (IAcc), indicating that the increase in anxiety from Safe to Threat is larger among individuals with higher IAcc (*p* = 0.041).

Our results showed a significant main effect of condition, indicating that subjective anxiety scores were higher in the Threat compared to the Safe condition (*b* = 9.38, *t*(29) = 3.50, *p* = 0.001) (Figure 2a). To assess whether the anxiety induction diminished over the course of the blocks, we incorporated block order and its interaction with the condition into the manipulation‐check model. The main effect of condition remained significant (*F*(1, 29) = 12.36, *p* = 0.001). In contrast, neither the main effect of block (*F*(11, 290) = 0.48, *p* = 0.913) nor the interaction between condition and block (*F*(11, 280) = 1.56, *p* = 0.111) reached significance. The difference in subjective anxiety between the Threat and Safe conditions remained stable across the 12 blocks, suggesting that habituation did not contribute to the observed behavioral results.

#### 3.2.2 Temporal Reproduction Task

Compared to the reference target duration (4 s), the reproduced durations were longer at 9 s (*b* = 3.28, *t*(34) = 14.17, *p* < 0.001) and 14 s (*b* = 5.58, *t*(30) = 14.24, *p* < 0.001). There was no significant main effect of condition (Safe, Threat) (*b* = 0.03, *t*(2731) = 0.37, *p* = 0.714) or significant interactions between target duration and condition (all *p* ≥ 0.22) on reproduced durations.

The accuracy of reproduction index (AR) was significantly lower at 9 s (*b* = −0.05, *t*(33) = −2.69, *p* = 0.011) and 14 s (*b* = −0.14, *t*(31) = −5.77, *p* < 0.001) as compared to the reference target duration (4 s). No significant main effect of condition (*b* = 0.01, *t*(2728) = 1.34, *p* = 0.18), and interactions between target duration and condition (all *p* ≥ 0.40) emerged.

#### 3.2.3 Effect of Trait Anxiety on Subjective Anxiety

Anxiety scores were significantly higher in the Threat compared to the Safe condition (*b* = 8.79, *t*(26) = 3.37, *p* = 0.002), and higher trait anxiety was associated with higher overall anxiety scores (*b* = 10.70, *t*(26) = 2.59, *p* = 0.015) (Figure S1). No significant interaction between condition and trait anxiety emerged (*b* = −3.02, *t*(26) = −1.16, *p* = 0.25).

#### 3.2.4 Effect of Interoception on Subjective Anxiety

When considering the effects of condition (Safe, Threat) and interoceptive accuracy (IAcc) on anxiety scores, a significant main effect of condition emerged, showing higher anxiety scores in the Threat compared to the Safe condition (*b* = 8.11, *t*(26) = 3.90, *p* < 0.001). There was no significant main effect of IAcc (*b* = −3.15, *t*(26) = −0.68, *p* = 0.503). A significant interaction between condition and IAcc (*b* = 4.50, *t*(26) = 2.15, *p* = 0.041) emerged, indicating that the increase in anxiety from Safe to Threat was larger in participants with higher interoceptive accuracy (Figure 2b).

When assessing the effect of condition and interoceptive sensibility (MAIA subscales), a significant main effect of condition emerged (*b* = 8.67, *t*(26) = 3.22, *p* = 0.003). None of the MAIA subscales showed significant main effects on anxiety (all *p* ≥ 0.13), and no significant interactions with condition were observed (*p* > 0.12) (details in Table S2).

#### 3.2.5 Effect of Subjective Anxiety and Interoception on Accuracy of Reproduction

A significant main effect of duration on accuracy of reproduction (AR) emerged for both 9 and 14 s, compared to the reference target duration (4 s) (9 s: *b* = −0.05, *t*(28) = −3.06, *p* = 0.005; 14 s: *b* = −0.14, *t*(28) = −5.88, *p* < 0.001). No significant main effect of anxiety (*b* = 0.003, *t*(328) = 0.52, *p* = 0.599), nor interaction effects between anxiety and duration (9 s × anxiety: *b* = 0.0004, *t*(199) = 0.05, *p* = 0.957; 14 s × anxiety: *b* = 0.003, *t*(395) = 0.33, *p* = 0.739) emerged.

**FIGURE 3:**
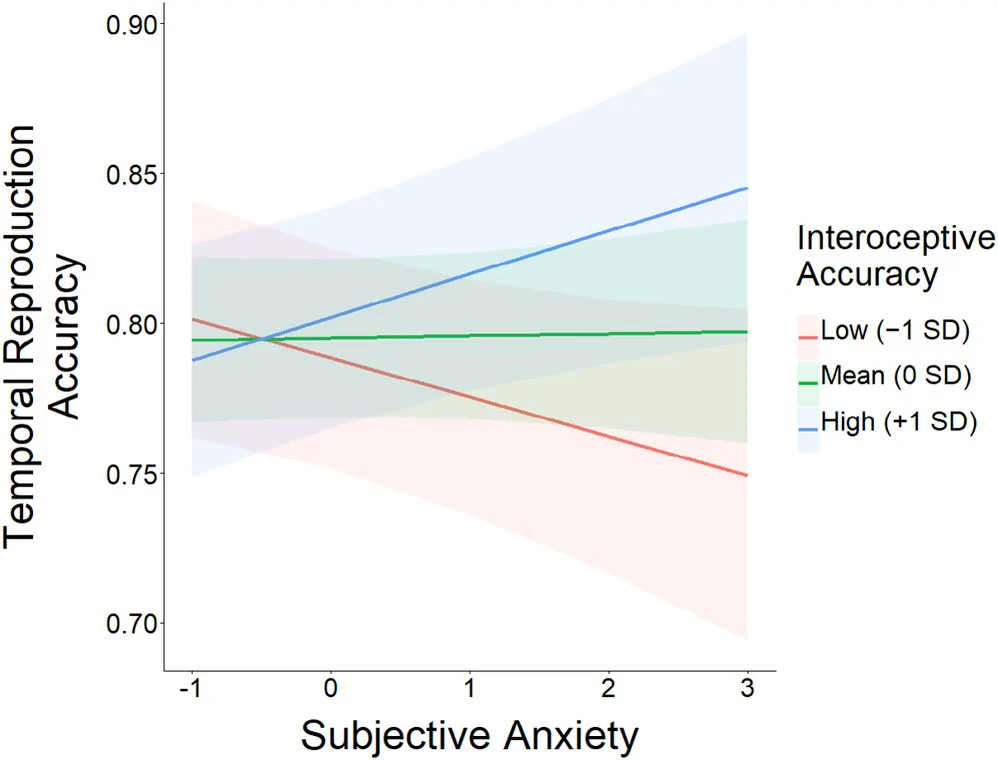
Interaction between subjective anxiety and interoceptive accuracy (IAcc) in predicting temporal reproduction accuracy (AR). Model‐predicted AR across the range of subjective anxiety at low (−1 SD), mean, and high (+1 SD) IAcc, illustrating that the association between subjective anxiety and AR varies as a function of IAcc, such that positive at high IAcc and negative at low IAcc (subjective anxiety × IAcc interaction, *p* = 0.004).

When adding the interoceptive accuracy (IAcc) index to the model, the analysis revealed a significant main effect of temporal duration (*p* ≤ 0.008). No significant main effects of anxiety (*b* = 0.0007, *t*(1497) = 0.16, *p* = 0.875) or IAcc (*b* = 0.006, *t*(26) = 0.52, *p* = 0.608) emerged. A significant interaction was observed between subjective anxiety and IAcc (*b* = 0.014, *t*(1622) = 2.86, *p* = 0.004) (Figure 3). The association between subjective anxiety and reproduction accuracy was more positive at higher interoceptive accuracy and more negative at lower interoceptive accuracy, such that individuals with greater interoceptive accuracy were able to maintain their accuracy even as their anxiety levels increased. The sensitivity analysis indicated that the power to detect this interaction was 18% at *b* = 0.005, 45% at *b* = 0.009, 62% at *b* = 0.011, 69% at *b* = 0.012, and 82% at *b* = 0.014. The observed effect (*b* = 0.014) thus lay at the threshold of reliable detectability given the present sample.

To address whether interoceptive sensibility also moderates the relationship between anxiety and temporal reproduction, we fitted eight separate models, each testing the interaction between subjective anxiety and one MAIA subscale on AR, using the same random‐effects structure as the interoceptive accuracy model, with FDR correction across subscales. No subscale × anxiety interaction reached significance, either uncorrected (all *p* ≥ 0.133) or after correction (all *p*_FDR ≥ 0.299), and the estimates were mixed in sign rather than systematically positive or negative. All interaction estimates (|*b*| ≤ 0.008) fell below the smallest effect this design could detect with adequate power (*b* = 0.014; see above). Higher Emotional Awareness was associated with reduced accuracy of reproduction overall (*b* = −0.028, *t*(27) = −2.37, *p* = 0.025), but it did not survive FDR correction (*p*_FDR = 0.203) (Table S3).

### 3.3 fMRI Results

To adopt a conservative approach and minimize false positives, *p*‐value thresholds were guided by FDR‐corrected results in control regions, specifically the 3rd, 4th, and lateral ventricles, and cerebral and cerebellar WM.

#### 3.3.1 ROI‐Based Whole‐Brain Results

**FIGURE 4:**
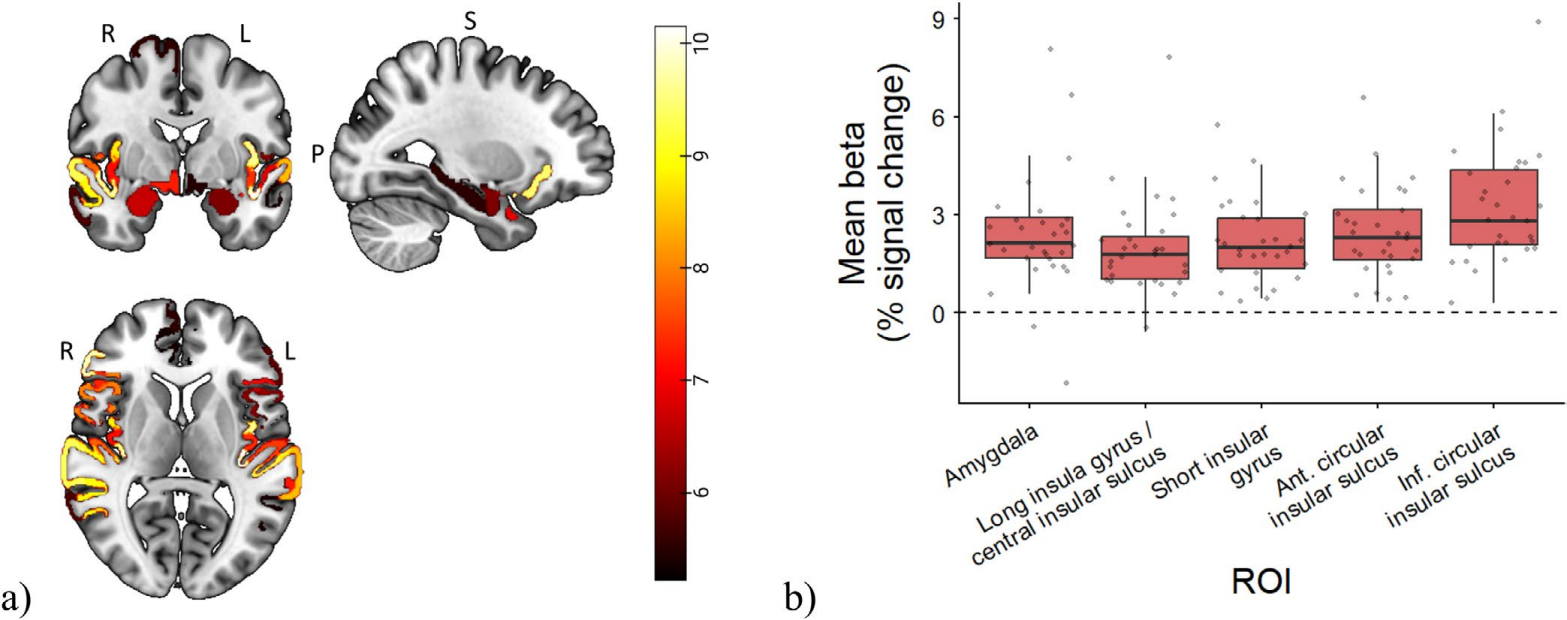
Threat‐block activations during scream events. (a) ROI‐wise Whole‐brain statistical maps showing greater activations during Scream delivery within Threat blocks (FDR‐corrected, *p* < 0.00004). Activation is most prominent in a salience/affective network including the amygdala, anterior insula/insular cortex, and cingulate regions, with additional involvement of frontal and sensorimotor territories. ROIs taken from cortical and subcortical parcellations (Destrieux atlas). (b) ROI summary of extracted responses during Scream delivery. Boxplots show ROI‐mean beta estimates (% signal change) for key regions (amygdala and insula subregions).

ROI‐based whole‐brain analysis revealed that both conditions (Safe, Threat) recruited a largely overlapping sensorimotor, auditory, and salience network during tone processing (encoding, reproduction), with additional limbic engagement specifically for scream delivery in Threat condition. In Safe condition, both encoding and reproduction phases engaged sensorimotor and auditory regions, including precentral gyrus and precentral sulci, transverse temporal gyrus and planum temporale, as well as mid‐ACC, anterior insula, and ventral diencephalon (all *p* ≤ 0.001 FDR‐corr). Under Threat, both encoding and reproduction phases were also associated with increased activation in a network encompassing premotor and somatosensory cortices, auditory regions, mid‐ACC, and anterior insula (all *p* ≤ 0.00004 FDR‐corr). During scream delivery, this network expanded to include frontal and limbic regions, with significant activation in amygdala, insula, and cingulate areas, indicating strong engagement of affective and salience circuits under Threat (*p* < 0.00004 FDR‐corr) (Figure 4). Full ROI‐wise statistics are reported in Tables S4 and S5.

#### 3.3.2 ROI‐Based ANOVA Results

**FIGURE 5:**
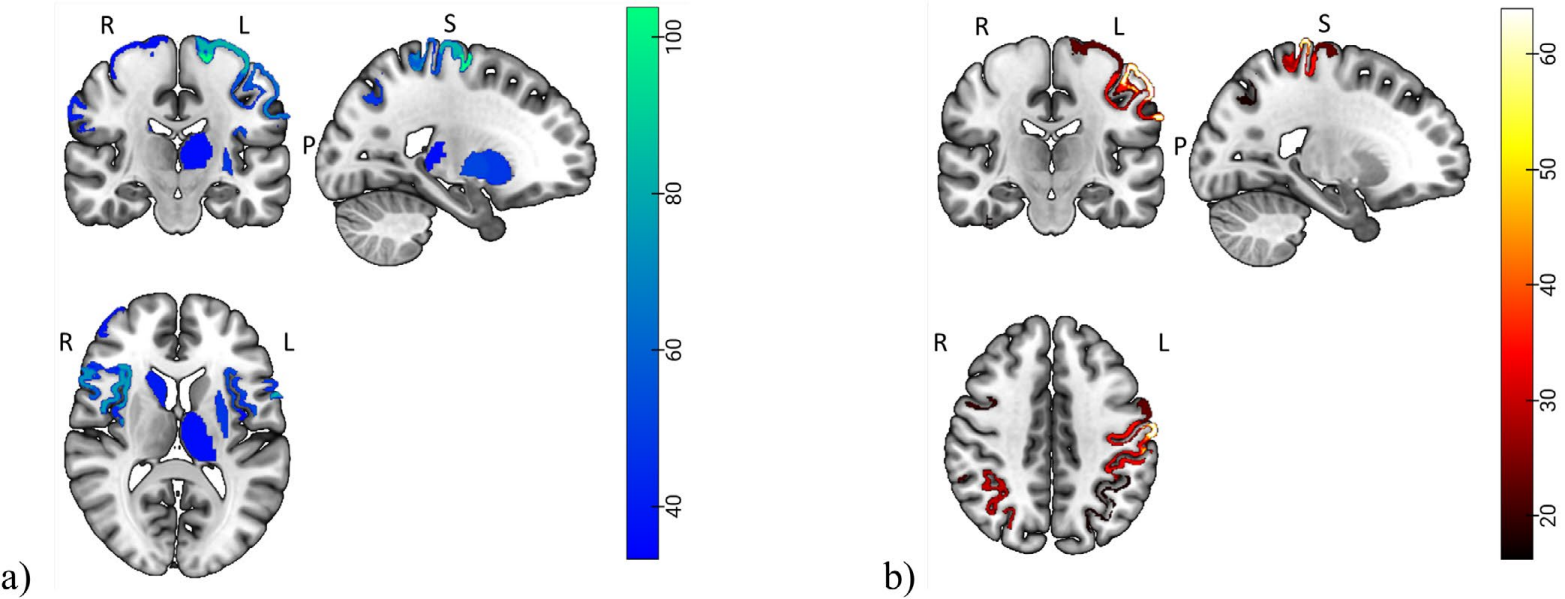
Reproduction > Encoding in Safe and Threat conditions. (a) ROI‐wise whole‐brain statistical map showing greater activation during Reproduction than Encoding within Safe blocks (FDR‐corrected, *p* < 0.00002). Increases were observed in sensorimotor cortices (pre‐ and postcentral gyri, central sulcus), parietal regions (intraparietal and transverse parietal sulci, supramarginal gyrus), insula (short gyrus and anterior circular sulcus), mid–anterior cingulate cortex, and cortico‐striato‐thalamic nuclei (putamen, caudate, pallidum, thalamus). (b) ROI‐wise whole‐brain contrast map showing greater activation during Reproduction than Encoding within Threat blocks (FDR‐corrected, *p* < 0.007). Increases were observed primarily in sensorimotor cortices and parietal regions, indicating stronger recruitment during reproduction than encoding under Threat. ROIs were defined using cortical (Destrieux) and subcortical parcellations. ROIs taken from cortical and subcortical parcellations (Destrieux atlas).

ROI‐based repeated‐measures ANOVAs with factors tone presentation phase (encoding vs. reproduction) and condition (Safe vs. Threat) revealed significant main effects of tone presentation phases in regions implicated in auditory, sensorimotor, and salience processing, including intraparietal and transverse parietal sulcus, supramarginal gyrus, precentral/postcentral gyrus, and mid‐ACC (all *p* ≤ 0.001 FDR‐corr). Additional significant phase effects were observed in the anterior insula, inferior/middle frontal cortex, putamen, and pallidum. In contrast, no significant main effects of condition or interactions between condition and tone presentation phase emerged, indicating that activation differences were primarily driven by tone presentation phase rather than by condition. Within‐condition pairwise contrasts further clarified these phase effects. In Safe condition, stronger activation in reproduction compared to encoding phase emerged in sensorimotor cortices (pre/postcentral gyri, central sulcus), parietal regions (intraparietal/transverse parietal sulcus, supramarginal gyrus), insula (short/anterior circular sulci), mid–anterior cingulate, and cortico‐striato‐thalamic nuclei (putamen, caudate, pallidum, thalamus) (all *p* ≤ 0.00002 FDR‐corr) (Figure 5a). In the Threat condition, increased activations during reproduction compared to encoding were limited to sensorimotor and parietal regions (all *p* ≤ 0.009 FDR‐corr) (Figure 5b). Full ROI‐wise statistics are reported in Tables S6 and S7.

**FIGURE 6:**
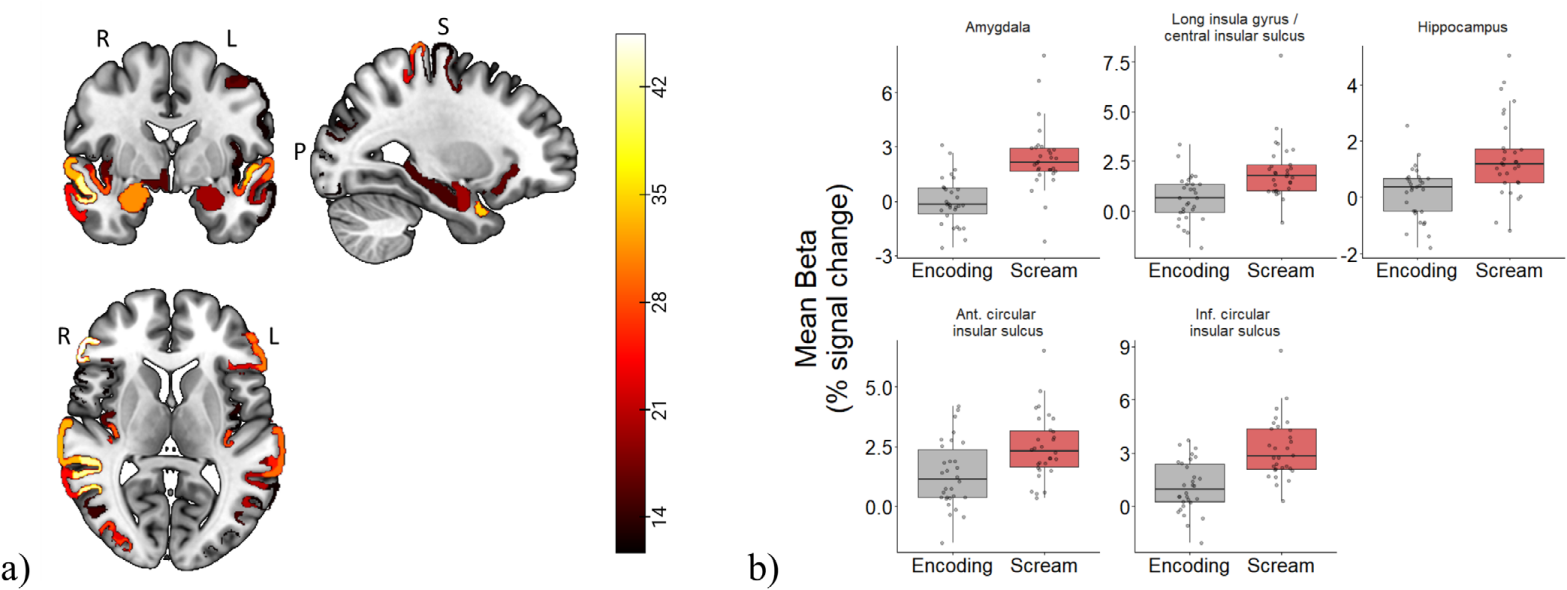
Scream > Encoding within Threat condition. (a) ROI‐wise whole‐brain statistical map showing greater activation for Scream than Encoding within Threat blocks (FDR‐corrected, *p* < 0.01). Increases were observed across the insula (long insular gyrus; central/superior/anterior circular sulci), cingulate/subparietal cortex, limb ic/subcortical regions (bilateral amygdala, hippocampus, ventral diencephalon), bilateral superior temporal cortex (planum polare, planum temporale, superior temporal sulcus), middle temporal gyrus, inferior frontal gyrus (triangular/orbital parts), and orbitofrontal/frontopolar cortices. A smaller subset of sensorimotor and occipito‐parietal regions (pre‐/postcentral gyri, central/intraparietal sulci, middle/inferior occipital and occipito‐temporal regions) showed decreases for Scream relative to Encoding. ROIs taken from cortical and subcortical parcellations (Destrieux atlas). (b) ROI summary of extracted responses within Threat blocks for the Encoding and Scream phases. Boxplots show ROI‐mean beta estimates (% signal change) for representative ROIs highlighted by the Scream > Encoding contrast (e.g., amygdala, hippocampus, and insula subregions).

**FIGURE 7:**
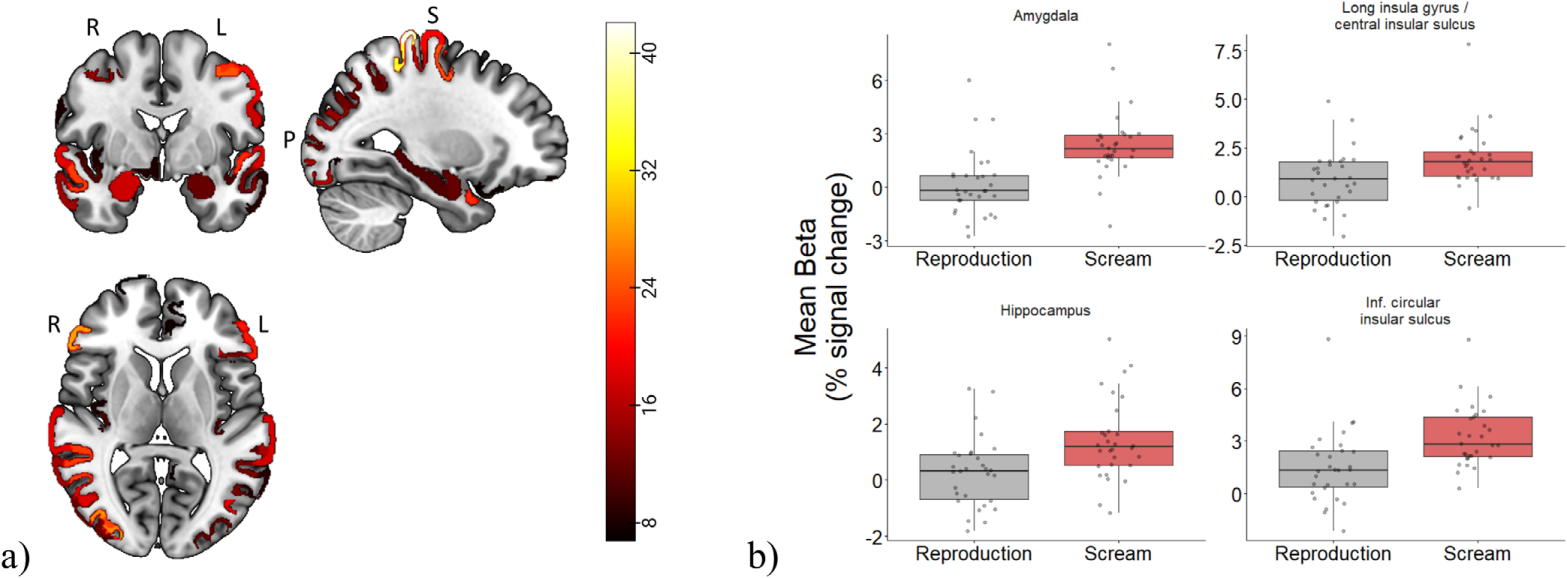
Scream > Reproduction within Threat condition. (a) ROI‐wise whole‐brain statistical map showing greater activation for Scream than Reproduction within Threat blocks (FDR‐corrected, *p* < 0.05). Increases were observed in a salience–limbic–temporo‐frontal network, including anterior and posterior insula (short and long gyri; anterior/inferior circular sulci), cingulate cortex, bilateral amygdala, hippocampus, ventral diencephalon, bilateral superior and middle temporal cortex (planum polare, planum temporale, superior temporal sulcus), inferior frontal gyrus (triangular, opercular, orbital parts), and orbitofrontal/superior frontal regions. ROIs taken from cortical and subcortical parcellations (Destrieux atlas). (b) ROI summary of extracted responses within Threat blocks for the Reproduction and Scream phases. Boxplots show ROI‐mean beta estimates (% signal change) for representative ROIs highlighted by the Scream > Reproduction contrast (e.g., amygdala, hippocampus, and insula subregions).

Under Threat, screams elicited stronger responses than non‐emotional tones in limbic, insular, temporo‐frontal, and cingulo‐parietal regions. Specifically, screams induced stronger activations than encoding tones in insular cortex (large insular gyrus, central/circular sulci), cingulate and subparietal cortex, limbic/subcortical structures (bilateral amygdala, hippocampus, ventral diencephalon), bilateral superior temporal cortex (planum polare/planum temporale and superior temporal sulcus), middle temporal gyrus, inferior frontal cortex (triangular/orbital parts), and orbitofrontal and frontopolar regions (all *p* ≤ 0.01 FDR‐corr) (Figure 6). A smaller subset of regions in sensorimotor and occipito‐parietal cortex showed reduced activity during scream delivery than encoding phase. Compared to reproduction tones, screams induced stronger activations in anterior and posterior insula (short and long gyri and anterior/inferior circular sulci), cingulate cortex, limbic and subcortical areas (bilateral amygdala, hippocampus, ventral diencephalon), bilateral superior and middle temporal cortex (planum polare/planum temporale and superior temporal sulcus), inferior frontal gyrus (triangular, opercular and orbital parts), and orbitofrontal and superior frontal regions (all *p* ≤ 0.05 FDR‐corr) (Figure 7). Reduced activity during scream delivery compared to reproduction phase was observed in primary sensorimotor and occipital regions. Full ROI‐wise statistics are reported in Tables S8 and S9.

## 4 Discussion

Using a novel Anxiety Temporal Reproduction task based on a modified ToS paradigm (Beaurenaut et al. 2020), we combined temporal reproduction performance, subjective anxiety ratings, interoceptive measures, and fMRI to test whether subjective anxiety alters timing and whether interoception moderates this relationship. Three findings anchor our results. First, the threat manipulation effectively increased subjective anxiety in Threat blocks compared to Safe blocks, validating the paradigm as a measure of threat‐related states. Second, contrary to our initial prediction, neither the Threat condition nor subjective anxiety had a significant effect on temporal reproduction accuracy. Third, the central finding was an interaction between subjective anxiety and interoceptive accuracy, such that individuals with higher interoceptive accuracy tended to preserve reproduction accuracy as anxiety increased. Conceptually, this pattern aligns with views that threat‐related states reallocate limited resources toward rapid detection and avoidance (Riemer et al. 2015; Robinson et al. 2013), and the moderating role of interoceptive accuracy fits with frameworks proposing that time perception results from an interplay of sensory information, bodily state signals, and expectations (Buhusi and Meck 2005; Lake et al. 2016; Sarigiannidis et al. 2020).

However, we observed inconsistent results between the pilot and the fMRI study about the interaction of anxiety and temporal durations. This is in line with mixed evidence on the effect of anxiety on timing, in which anxiety shortened perceived duration under unpredictable threat (Sarigiannidis et al. 2020), lengthened it for threatening cues or anticipated aversive events (Bar‐Haim et al. 2010; Harjunen et al. 2022), and sometimes left accuracy unchanged (Lake et al. 2016), depending on threat predictability, interval range, and task demands (Liu and Li 2019). Also, the MRI environment itself can elevate physiological arousal (Muehlhan et al. 2011) and alter auditory processing due to scanner acoustic noise (Skouras et al. 2013), potentially shifting responses relative to the behavioral setting.

Individual differences can clarify when anxiety matters (Tabor et al. 2019). Individuals with higher trait anxiety reported greater subjective anxiety during the task (Endler and Kocovski 2001; Lau et al. 2006; Leal et al. 2017). Crucially, interoceptive accuracy amplified the subjective effect of threat, as individuals with higher interoceptive accuracy experienced greater anxiety when feeling threatened, suggesting that enhanced sensitivity to bodily signals amplifies the salience of threat‐related body changes and intensifies emotional responses (Mennella et al. 2025; Pollatos, Gramann, and Schandry 2007; Pollatos, Herbert, et al. 2007; Pollatos et al. 2014; Riemer et al. 2015; Ventura‐Bort et al. 2021). Interoceptive sensibility, however, showed no reliable association with subjective anxiety or its impact on temporal reproduction. This indicates that the objective and subjective aspects of interoception contribute differently to the relationship between anxiety and timing (Garfinkel et al. 2015; Mehling et al. 2012, 2018). The interplay between anxiety and interoception was also reflected in temporal performance (Mennella et al. 2025). Although neither subjective anxiety nor interoceptive accuracy alone predicted temporal reproduction accuracy, their interaction was significant, such that the association between anxiety and accuracy was negative at low interoceptive accuracy and positive at high interoceptive accuracy. In other words, individuals with better interoceptive accuracy maintained their accuracy in reproduction, even as their anxiety levels increased, whereas those with lower interoceptive accuracy experienced a decline in accuracy. Bringing this together with the amplified anxiety response reported above, heightened interoceptive accuracy appears to intensify the subjective experience of threat while mitigating its impact on time perception, reconciling evidence that interoceptive accuracy amplifies affective responses with evidence that it protects against emotion‐related temporal distortion. This is consistent with proposals that interoception may influence how emotion translates into cognition (Di Lernia et al. 2018; Uraguchi et al. 2022).

At the neural level, two findings are supported by the ROI‐based analyses of the present data. Scream events engaged salience‐ and limbic‐related networks, and the phase (reproduction vs. encoding) contrast was present in both contexts, encompassing a broader set of regions under Safe and a more circumscribed sensorimotor–parietal set under Threat, although the condition × phase interaction was not statistically significant.

Specifically, scream delivery engaged a distributed network including the amygdala, hippocampus, insula, ventral diencephalon, and temporal cortices, highlighting the sensitivity of our paradigm as screams recruited regions central to affective salience processing (Phelps and LeDoux 2005; van Oort et al. 2017). The absence of activations in regions unrelated to emotional processing underscores the specificity of the response. Also, scream‐related brain activations were significantly stronger than non‐emotional sounds during both encoding and reproduction phases, indicating that screams drove neural reactivity beyond task demands. Comparisons between the encoding and reproduction phases revealed phase‐related differences across conditions. In the Safe condition, reproduction exceeded encoding in sensorimotor, parietal, anterior insula, mid–ACC, and cortico‐striato‐thalamic nuclei, consistent with models where reproducing temporal information adds read‐out, decision, and motor planning to encoded durations (Buhusi and Meck 2005; Coull et al. 2011). Under Threat, the same phase contrast appeared more restricted, mainly in sensorimotor and parietal areas. This difference is descriptive, as it reflects which regions survived threshold within each condition tested separately, rather than a significant condition × phase interaction, which we did not observe. The pattern is consistent with two convergent lines of evidence. First, the regions that remained under Threat are those most consistently implicated in threat‐related action readiness. There is evidence of a direct pathway linking the amygdala to precentral and postcentral motor structures (Grèzes et al. 2014), and sustained threat increases both the excitability and whole‐brain connectivity of the intraparietal sulcus (Balderston et al. 2017), a region that also emerged in our phase contrast. Second, acute threats tend to reallocate processing resources toward salience and action‐relevant systems at the cost of associative and control networks (Hermans et al. 2011, 2014; Menon and Uddin 2010; Pessoa 2009; Seeley et al. 2007). On this account, a salience response to screams would be added on top of ongoing timing activity, such that the phase effect itself persists under Threat while its spatial extent narrows toward the action‐relevant core of the reproduction network. Encoding may engage posterior insula accumulation within the timing loop, whereas reproduction may additionally recruit anterior insula and dACC for read‐out and control (Vicario et al. 2020; Wittmann et al. 2010). However, these interpretations are speculative. Our design did not include measures of interoceptive processing during the task, and the phase contrast under Threat was descriptive rather than a significant condition × phase interaction, so the insular and cingulate contributions proposed here go beyond what the present data can establish and require direct testing.

Our null effect of threat on mean accuracy can be interpreted in light of the type of task. Temporal reproduction links timing to motor output and multisensory comparison, and may be less sensitive to perceptual lengthening or shortening typically observed in verbal estimation or bisection tasks, in which emotional stimuli often affect the point of subjective equality (Droit‐Volet and Gil 2009; Droit‐Volet and Meck 2007). Indeed, reproduction and estimation of identical intervals engage partially distinct neural circuits (Bueti et al. 2008), and even variations in the reproduction method can lead to different levels of accuracy and variability (Mioni et al. 2014). A meta‐analysis also revealed that activation patterns vary systematically based on whether the response is motor or perceptual (Wiener et al. 2010). Supporting this, individuals with anxiety show different time perception depending on whether reproduction or production methods are used (Mioni et al. 2016). The motor and comparison demand of reproduction may also explain why the reproduction‐encoding contrast is localized in sensorimotor and parietal regions, and why the impact of threat on this pattern seems to focus on areas related to action planning and integration. It is still an open question whether a duration‐judgment task could uncover anxiety effects that may not be noticeable in this context, which requires further investigation in future research.

More broadly, the everyday experience of time “slowing down” during stress or “getting away” when overwhelmed (Sarigiannidis et al. 2020) may depend on how strongly people attend to and interpret their bodily signals. Our results on the moderating role of interoception suggest that bodily awareness is not uniformly helpful or harmful, but can shape whether anxiety impairs or preserves performance (Lin et al. 2025). This can inform interoception‐targeted interventions (e.g., mindfulness‐based approaches, biofeedback) that can modify anxiety symptoms (Dobrushina et al. 2024; Heim et al. 2023; Schillings et al. 2022; Sugawara et al. 2020), such as in clinical populations where altered interoception is linked to anxiety, stress‐related psychopathology, and difficulties with uncertainty (Clemente et al. 2024; Jenkinson et al. 2024).

Taken together, our behavioral and neural results are consistent with an account in which threat enhances emotional arousal that may or may not bias time estimation, depending on interoceptive ability. Unpredictable screams raised subjective anxiety and selectively engaged salience–limbic circuits without changing mean temporal accuracy. Nonetheless, interoception moderated the impact of subjective anxiety on timing, such that for individuals who strongly tracked internal signals, temporal performance was affected by anxiety. At a neural level, encoding‐reproduction contrasts highlighted cortico‐striatal and limbic contributions to timing (Buhusi and Meck 2005; Coull et al. 2011). Behavioral and neural evidence thus identifies a possible pathway through which emotions may influence time perception.

### 4.1 Limitations and Future Directions

Our study benefited from the use of the “Threat of Scream” paradigm (Beaurenaut et al. 2020), which reliably induced anxiety while maintaining ecological validity by resembling real‐world unpredictability. By combining behavioral, self‐rated, and fMRI measures, we were able to capture converging evidence on how threat‐induced anxiety shapes behavioral performance and neural dynamics. In addition to this, the integration of interoceptive accuracy provided novel insights into individual differences in the link between bodily awareness, anxiety, and temporal processing. Nevertheless, some limitations should be acknowledged. Several factors limit the findings related to individual differences. The sample size (*N* = 30) was modest for detecting differences between subjects, and seven participants completed only five heartbeat‐tracking trials instead of six due to a recording error, adding noise to the interoceptive accuracy index. Additionally, the pilot study did not include measures of interoception or trait anxiety, so it could not inform the expected size of the interaction effects. A sensitivity analysis indicated that power reached the conventional 80% level only for effects around the magnitude we observed (*b* = 0.014) and was substantially lower for smaller effects (e.g., ~45% at *b* = 0.009). The observed interaction therefore lies at the threshold of reliable detectability given our sample size. Accordingly, the interoception‐related effects should be regarded as exploratory and require replication in larger samples.

The same asymmetry applies to our neuroimaging analyses. The individual‐difference factors that shaped behavior (i.e., subjective anxiety and interoceptive accuracy) were not entered into the primary fMRI models. This reflects statistical power rather than theoretical choice. Within‐subject contrasts (phase, condition, scream) draw on thousands of trials, whereas relating between‐subject moderators to neural responses depends on the number of participants. With the present sample, such analyses are underpowered. Exploratory brain–behavior analyses relating regional activation to reproduction accuracy are reported in the Supporting Information, but none survived correction for multiple comparisons. Interoceptive sensibility (MAIA) was examined in the same way and showed no moderation of either subjective anxiety or temporal reproduction accuracy after correction for multiple comparisons. Beyond sample size, our participants were healthy young adults with low–to–moderate trait anxiety, and the imaging analyses emphasized ROIs rather than network connectivity. Future studies should recruit more diverse cohorts, test ecologically relevant settings, and systematically vary threat predictability and controllability. Methodologically, combining fMRI with physiological measures (EDA, ECG) and connectivity models could help deepen understanding of the phenomenon. Interventional work that shifts interoceptive focus (e.g., mindfulness, biofeedback) should also test whether enhancing bodily awareness alters the anxiety‐timing relationship.

## Funding

This work was supported by Tamkeen (CG012).

## Supporting information


**Figure S1:** Subjective anxiety as a function of trait anxiety and condition. Model predicted subjective anxiety plotted against trait anxiety (STAI‐Y2), separately for the Safe and Threat conditions. Subjective anxiety was higher under Threat than Safe (*b* = 8.79, *t*(26) = 3.37, *p* = 0.002), and higher trait anxiety was associated with higher subjective anxiety across conditions (*b* = 10.70, *t*(26) = 2.59, *p* = 0.015). The condition × trait anxiety interaction was not significant (*b* = −3.02, *t*(26) = −1.16, *p* = 0.25), indicating that the association between trait anxiety and subjective anxiety did not differ reliably between conditions. Estimates are from a linear mixed‐effects model with condition, trait anxiety, and their interaction as predictors, and by‐subject random intercepts and random slopes for condition.
**Table S1:** Destrieux atlas ROIs. List of regions of interest (ROIs) from the Destrieux cortical parcellation (Destrieux et al. 2010), including ROI labels with centroid coordinates (x, y, z) in MNI space (mm).
**Table S2:** Effect of interoceptive sensibility (MAIA subscales) on subjective anxiety. Results of eight linear mixed‐effects models, each testing the interaction between condition (Safe, Threat) and one MAIA subscale on subjective anxiety ratings (*N* = 28). All models included by‐subject random intercepts and random slopes for condition. Benjamini–Hochberg FDR correction was applied separately across the eight subscale main effects and the eight interaction terms.
**Table S3:** Interoceptive sensibility (MAIA subscales) and temporal reproduction accuracy. Results of eight separate linear mixed‐effects models, each testing the interaction between subjective anxiety and one MAIA subscale on accuracy of reproduction (AR), controlling for target duration (*N* = 28). All models included by‐subject random intercepts and random slopes for target duration. Benjamini–Hochberg FDR correction was applied separately across the eight subscale main effects and the eight interaction terms.
**Table S4:** ROI‐based whole‐brain results in Safe condition. For each task regressor (encoding, reproduction), the table lists brain regions of interest (ROIs) showing effects after false discovery rate (FDR) correction, along with MNI coordinates (X, Y, Z). ROIs are labeled using the Destrieux atlas nomenclature.
**Table S5:** ROI‐based whole‐brain results in Threat condition. For each task regressor (encoding, reproduction, scream), the table lists brain regions of interest (ROIs) showing effects after false discovery rate (FDR) correction, along with MNI coordinates (X, Y, Z). ROIs are labeled using the Destrieux atlas nomenclature.
**Table S6:** ROI‐based repeated‐measures ANOVA results comparing encoding vs. reproduction in Safe condition. The table reports regions showing regressor effects (encoding vs. reproduction), listing the direction of the contrast, ROI label, degrees of freedom (df), *F*‐values, uncorrected *p*‐values, and FDR‐corrected *p*‐values, together with MNI coordinates (X, Y, Z). ROIs are labeled according to the Destrieux atlas nomenclature.
**Table S7:** ROI‐based repeated‐measures ANOVA results comparing encoding vs. reproduction in Threat condition. The table reports regions showing regressor effects (encoding vs. reproduction), listing the direction of the contrast, ROI label, degrees of freedom (df), *F*‐values, uncorrected *p*‐values, and FDR‐corrected *p*‐values, together with MNI coordinates (X, Y, Z). ROIs are labeled according to the Destrieux atlas nomenclature.
**Table S8:** ROI‐based repeated‐measures ANOVA results comparing scream vs. encoding in Threat condition. The table reports regions showing regressor effects (scream vs. encoding), listing the direction of the contrast, ROI label, degrees of freedom (df), *F*‐values, uncorrected *p*‐values, and FDR‐corrected *p*‐values, together with MNI coordinates (X, Y, Z). ROIs are labeled according to the Destrieux atlas nomenclature.
**Table S9:** ROI‐based repeated‐measures ANOVA results comparing scream vs. reproduction in Threat condition. The table reports regions showing regressor effects (scream vs. reproduction), listing the direction of the contrast, ROI label, degrees of freedom (df), *F*‐values, uncorrected *p*‐values, and FDR‐corrected *p*‐values, together with MNI coordinates (X, Y, Z). ROIs are labeled according to the Destrieux atlas nomenclature.
**Table S10:** Brain‐behavior analysis: ROIs on accuracy of reproduction, based on phase (encoding, reproduction) and condition (safe/threat). For each of 15 a priori ROIs, comprising salience regions (amygdala, insular subregions, cingulate subregions) and the sensorimotor and parietal regions implicated in our phase contrast (precentral gyrus, postcentral gyrus, central sulcus, paracentral lobule, superior parietal gyrus, intraparietal sulcus), we correlated subject‐level activation during each phase (encoding, reproduction, and scream) with mean AR in the corresponding condition. Across 75 tests, five correlations emerged as significant uncorrected, but none survived FDR correction applied across the full family (*p*_FDR ≥ 0.26).
**Table S11:** Brain‐behavior analysis. For each of 15 a priori ROIs, we fitted models testing whether neural response moderates the within‐subject relationship between subjective anxiety and accuracy of reproduction (anxiety × ROI), and the full three‐way interaction additionally including interoceptive accuracy (anxiety × interoception × ROI) (75 models per specification). No interaction reached significance (two‐way: *p* ≥ 0.27, three way: *p* ≥ 0.12).

## Data Availability

The data that support the findings of this study are openly available in EmoTime at https://osf.io/8u9pt/overview.
