# Supplementary material for: The Emotional Experience of Time: Effects of Anxiety and Interoceptive Ability on Temporal Perception
Source: Hum Brain Mapp. 2026 Sep 24;47(14):e70650. doi: 10.1002/hbm.70650 (PMC13612850; doi:10.1002/hbm.70650)
Supplement: Supplementary file 1 [file HBM-47-e70650-s001.pdf]

Figure S1 - Subjective anxiety as a function of trait anxiety and condition. Model-predicted subjective anxiety plotted against trait anxiety (STAI-Y2), separately for the Safe and Threat conditions. Subjective anxiety was higher under Threat than Safe ($b = 8.79$, $t(26) = 3.37$, $p = .002$), and higher trait anxiety was associated with higher subjective anxiety across conditions ($b = 10.70$, $t(26) = 2.59$, $p = .015$). The condition × trait anxiety interaction was not significant ($b = -3.02$, $t(26) = -1.16$, $p = .25$), indicating that the association between trait anxiety and subjective anxiety did not differ reliably between conditions. Estimates are from a linear mixed-effects model with condition, trait anxiety, and their interaction as predictors, and by-subject random intercepts and random slopes for condition.

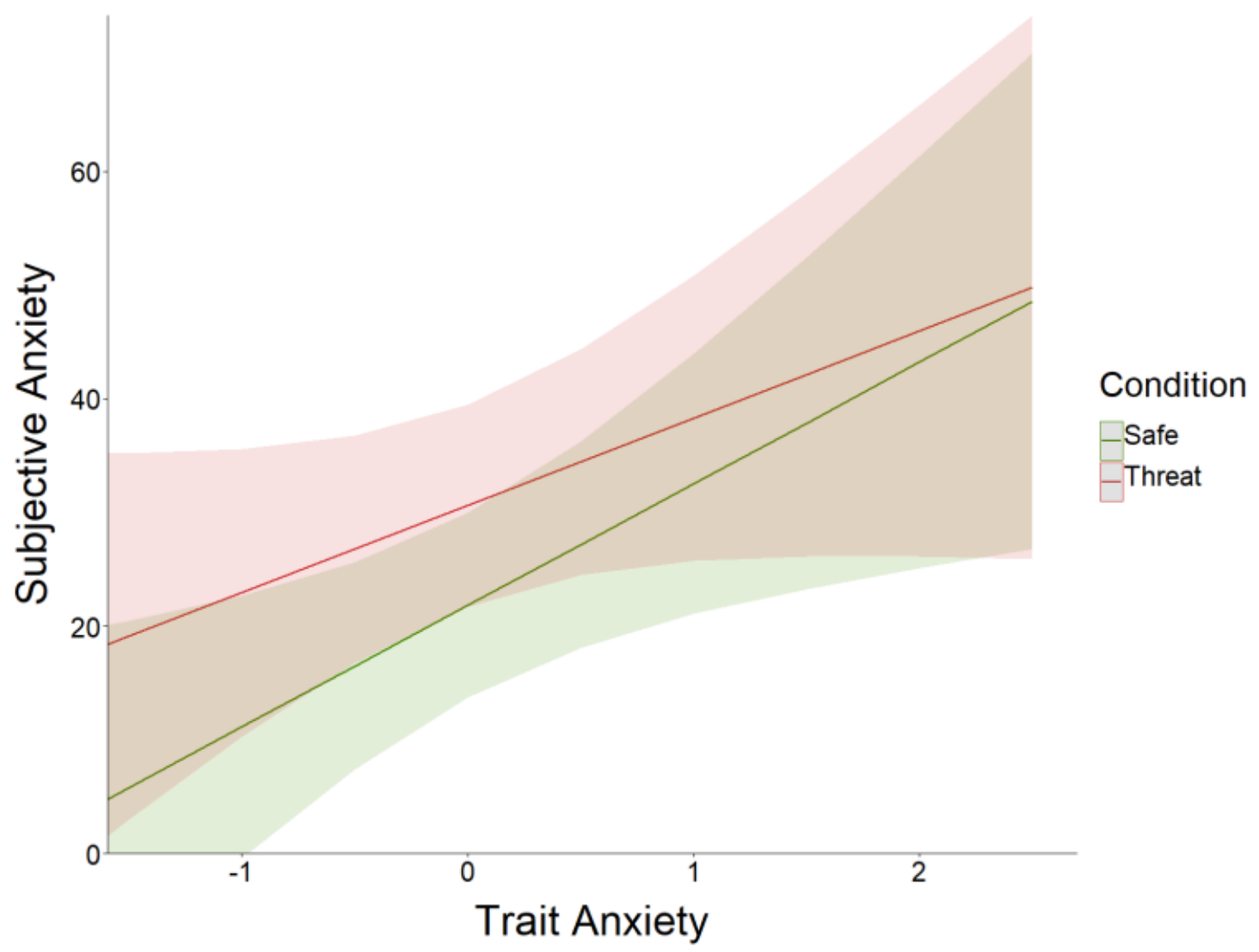

Table S1 – Destrieux atlas ROIs

List of regions of interest (ROIs) from the Destrieux cortical parcellation (Destrieux et al., 2010), including ROI labels with centroid coordinates (x, y, z) in MNI space (mm).

ROI	X	Y	Z
3rd-Ventricle	0	-11	-3
4th-Ventricle	-1	-46	-35
Brain-Stem	0	-31	-34
CC_Anterior	0	25	5
CC_Central	0	-2	22
CC_Mid_Anterior	0	13	19
CC_Mid_Posterior	0	-18	22
CC_Posterior	0	-35	17
CSF	0	-19	10
ctx_lh_G_and_S_cingul-Ant	-8	41	6
ctx_lh_G_and_S_cingul-Mid-Ant	-7	16	35
ctx_lh_G_and_S_cingul-Mid-Post	-7	-11	41
ctx_lh_G_and_S_frontomargin	-24	61	-10
ctx_lh_G_and_S_occipital_inf	-39	-83	-10
ctx_lh_G_and_S_paracentral	-8	-36	68
ctx_lh_G_and_S_subcentral	-57	-11	15
ctx_lh_G_and_S_transv_frontopol	-17	67	-3
ctx_lh_G_cingul-Post-dorsal	-3	-38	32
ctx_lh_G_cingul-Post-ventral	-8	-48	6
ctx_lh_G_cuneus	-4	-84	19
ctx_lh_G_front_inf-Opercular	-51	12	10
ctx_lh_G_front_inf-Orbital	-46	29	-10
ctx_lh_G_front_inf-Triangul	-52	31	4
ctx_lh_G_front_middle	-38	34	34
ctx_lh_G_front_sup	-9	27	45
ctx_lh_G_Ins_lg_and_S_cent_ins	-38	-7	-2
ctx_lh_G_insular_short	-38	10	-4
ctx_lh_G_occipital_middle	-39	-88	12
ctx_lh_G_occipital_sup	-15	-92	29
ctx_lh_G_oc-temp_lat-fusifor	-36	-55	-19
ctx_lh_G_oc-temp_med-Lingual	-10	-73	-6
ctx_lh_G_oc-temp_med-Parahip	-23	-16	-26
ctx_lh_G_orbital	-31	35	-19
ctx_lh_G_pariet_inf-Angular	-44	-69	41
ctx_lh_G_pariet_inf-Supramar	-60	-38	34
ctx_lh_G_parietal_sup	-26	-61	59
ctx_lh_G_postcentral	-47	-25	55
ctx_lh_G_precentral	-43	-9	53
ctx_lh_G_precuneus	-5	-60	45
ctx_lh_G_rectus	-5	36	-23
ctx_lh_G_subcallosal	-12	8	-13
ctx_lh_G_temp_sup-G_T_transv	-49	-18	8
ctx_lh_G_temp_sup-Lateral	-58	-7	-7
ctx_lh_G_temp_sup-Plan_polar	-41	8	-20
ctx_lh_G_temp_sup-Plan_tempo	-59	-38	18
ctx_lh_G_temporal_inf	-55	-40	-23

ctx_lh_G_temporal_middle	-63	-25	-13
ctx_lh_Lat_Fis-ant-Horizont	-44	32	-4
ctx_lh_Lat_Fis-ant-Vertical	-48	21	11
ctx_lh_Lat_Fis-post	-41	-34	20
ctx_lh_Pole_occipital	-16	-101	-4
ctx_lh_Pole_temporal	-33	6	-42
ctx_lh_S_calcarine	-15	-69	5
ctx_lh_S_central	-39	-19	46
ctx_lh_S_cingul-Marginalis	-13	-38	49
ctx_lh_S_circular_insula_ant	-31	22	-10
ctx_lh_S_circular_insula_inf	-41	-12	-5
ctx_lh_S_circular_insula_sup	-36	6	11
ctx_lh_S_collat_transv_ant	-41	-20	-30
ctx_lh_S_collat_transv_post	-24	-81	-7
ctx_lh_S_front_inf	-41	27	20
ctx_lh_S_front_middle	-29	46	19
ctx_lh_S_front_sup	-24	22	46
ctx_lh_S_interm_prim-Jensen	-52	-41	46
ctx_lh_S_intrapariet_and_P_trans	-32	-58	43
ctx_lh_S_oc_middle_and_Lunatus	-33	-87	8
ctx_lh_S_oc_sup_and_transversal	-27	-84	21
ctx_lh_S_occipital_ant	-41	-70	7
ctx_lh_S_oc-temp_lat	-44	-50	-14
ctx_lh_S_oc-temp_med_and_Lingual	-28	-48	-11
ctx_lh_S_orbital_lateral	-42	47	-5
ctx_lh_S_orbital_med-olfact	-13	25	-20
ctx_lh_S_orbital-H_Shaped	-26	40	-16
ctx_lh_S_parieto_occipital	-15	-68	25
ctx_lh_S_pericallosal	-6	-4	17
ctx_lh_S_postcentral	-42	-33	46
ctx_lh_S_precentral-inf-part	-45	4	30
ctx_lh_S_precentral-sup-part	-29	-10	56
ctx_lh_S_suborbital	-8	40	-14
ctx_lh_S_subparietal	-10	-49	38
ctx_lh_S_temporal_inf	-55	-34	-16
ctx_lh_S_temporal_sup	-51	-43	8
ctx_lh_S_temporal_transverse	-53	-23	5
ctx_rh_G_and_S_cingul-Ant	7	41	8
ctx_rh_G_and_S_cingul-Mid-Ant	7	17	35
ctx_rh_G_and_S_cingul-Mid-Post	7	-8	43
ctx_rh_G_and_S_frontomargin	23	62	-11
ctx_rh_G_and_S_occipital_inf	43	-76	-12
ctx_rh_G_and_S_paracentral	7	-35	69
ctx_rh_G_and_S_subcentral	57	-8	14
ctx_rh_G_and_S_transv_frontopol	17	68	0
ctx_rh_G_cingul-Post-dorsal	3	-36	34
ctx_rh_G_cingul-Post-ventral	8	-48	7
ctx_rh_G_cuneus	7	-80	21
ctx_rh_G_front_inf-Opercular	51	14	9
ctx_rh_G_front_inf-Orbital	48	35	-11
ctx_rh_G_front_inf-Triangul	52	33	5

ctx_rh_G_front_middle	38	36	33
ctx_rh_G_front_sup	10	28	47
ctx_rh_G_Ins_lg_and_S_cent_ins	39	-4	-3
ctx_rh_G_insular_short	38	12	-5
ctx_rh_G_occipital_middle	45	-79	15
ctx_rh_G_occipital_sup	20	-89	31
ctx_rh_G_oc-temp_lat-fusifor	35	-48	-21
ctx_rh_G_oc-temp_med-Lingual	10	-67	-4
ctx_rh_G_oc-temp_med-Parahip	24	-13	-29
ctx_rh_G_orbital	29	38	-19
ctx_rh_G_pariet_inf-Angular	50	-58	42
ctx_rh_G_pariet_inf-Supramar	59	-29	36
ctx_rh_G_parietal_sup	25	-60	62
ctx_rh_G_postcentral	47	-21	54
ctx_rh_G_precentral	42	-7	53
ctx_rh_G_precuneus	6	-60	47
ctx_rh_G_rectus	4	38	-24
ctx_rh_G_subcallosal	5	12	-13
ctx_rh_G_temp_sup-G_T_transv	48	-14	7
ctx_rh_G_temp_sup-Lateral	59	-4	-7
ctx_rh_G_temp_sup-Plan_polar	36	9	-20
ctx_rh_G_temp_sup-Plan_tempo	61	-28	20
ctx_rh_G_temporal_inf	55	-35	-25
ctx_rh_G_temporal_middle	62	-23	-14
ctx_rh_Lat_Fis-ant-Horizont	41	29	0
ctx_rh_Lat_Fis-ant-Vertical	48	23	10
ctx_rh_Lat_Fis-post	42	-27	20
ctx_rh_Pole_occipital	20	-95	-3
ctx_rh_Pole_temporal	32	9	-42
ctx_rh_S_calcarine	18	-65	7
ctx_rh_S_central	37	-17	47
ctx_rh_S_cingul-Marginalis	12	-37	50
ctx_rh_S_circular_insula_ant	31	23	-10
ctx_rh_S_circular_insula_inf	42	-8	-7
ctx_rh_S_circular_insula_sup	37	9	9
ctx_rh_S_collat_transv_ant	41	-18	-29
ctx_rh_S_collat_transv_post	26	-79	-8
ctx_rh_S_front_inf	40	27	23
ctx_rh_S_front_middle	30	47	19
ctx_rh_S_front_sup	23	25	45
ctx_rh_S_interm_prim-Jensen	52	-41	46
ctx_rh_S_intrapariet_and_P_trans	30	-56	47
ctx_rh_S_oc_middle_and_Lunatus	37	-79	10
ctx_rh_S_oc_sup_and_transversal	30	-79	23
ctx_rh_S_occipital_ant	46	-64	5
ctx_rh_S_oc-temp_lat	45	-47	-15
ctx_rh_S_oc-temp_med_and_Lingual	28	-48	-11
ctx_rh_S_orbital_lateral	42	44	-3
ctx_rh_S_orbital_med-olfact	12	27	-19
ctx_rh_S_orbital-H_Shaped	25	40	-16
ctx_rh_S_parieto_occipital	18	-66	28

ctx_rh_S_pericallosal	6	-7	19
ctx_rh_S_postcentral	38	-30	49
ctx_rh_S_precentral-inf-part	45	7	30
ctx_rh_S_precentral-sup-part	29	-9	56
ctx_rh_S_suborbital	5	39	-18
ctx_rh_S_subparietal	9	-48	38
ctx_rh_S_temporal_inf	55	-33	-16
ctx_rh_S_temporal_sup	51	-38	10
ctx_rh_S_temporal_transverse	53	-18	7
Left-Accumbens-area	-9	11	-8
Left-Amygdala	-24	-5	-20
Left-Caudate	-14	10	10
Left-Cerebellum-Cortex	-25	-63	-37
Left-Cerebellum-White-Matter	-20	-57	-33
Left-Cerebral-White-Matter	-29	-19	19
Left-choroid-plexus	-22	-31	7
Left-Hippocampus	-26	-23	-14
Left-Inf-Lat-Vent	-33	-20	-15
Left-Lateral-Ventricle	-15	-17	14
Left-Pallidum	-21	-5	-1
Left-Putamen	-26	1	0
Left-Thalamus-Proper	-11	-19	7
Left-VentralDC	-11	-16	-10
Left-vessel	-29	-3	-9
Optic-Chiasm	-1	1	-19
Right-Accumbens-area	8	12	-8
Right-Amygdala	23	-4	-20
Right-Caudate	13	11	9
Right-Cerebellum-Cortex	24	-63	-37
Right-Cerebellum-White-Matter	19	-56	-33
Right-Cerebral-White-Matter	29	-18	19
Right-choroid-plexus	20	-24	8
Right-Hippocampus	26	-22	-14
Right-Inf-Lat-Vent	31	-14	-18
Right-Lateral-Ventricle	14	-14	14
Right-Pallidum	21	-4	-2
Right-Putamen	26	2	0
Right-Thalamus-Proper	11	-17	7
Right-VentralDC	10	-15	-10
Right-vessel	29	-2	-10
WM-hypointensities	-4	-14	19

Table S2 - Effect of interoceptive sensibility (MAIA subscales) on subjective anxiety. Results of eight linear mixed-effects models, each testing the interaction between condition (Safe, Threat) and one MAIA subscale on subjective anxiety ratings (N = 28). All models included by-subject random intercepts and random slopes for condition. Benjamini–Hochberg FDR correction was applied separately across the eight subscale main effects and the eight interaction terms.

Subscale	*Term*	*b*	*95% CI low*	*95% CI high*	*t*	*df*	*p*	*p (FDR)*
Not-Distracting	Intercept	22.085	13.419	30.751	4.99	26	<.001	
	Condition (Threat)	8.670	3.391	13.949	3.22	26	.003	
	MAIA subscale	7.090	-1.732	15.911	1.58	26	.127	.628
	Condition × subscale	-1.005	-6.378	4.369	-0.37	26	.717	.956
Noticing	Intercept	22.047	13.084	31.011	4.82	26	<.001	
	Condition (Threat)	8.670	3.378	13.963	3.21	26	.003	
	MAIA subscale	3.608	-5.357	12.573	0.79	26	.437	.628
	Condition × subscale	0.133	-5.160	5.426	0.05	26	.961	.961
Body Listening	Intercept	21.963	12.981	30.946	4.79	26	<.001	
	Condition (Threat)	8.724	3.465	13.983	3.25	26	.003	
	MAIA subscale	3.358	-5.644	12.360	0.73	26	.471	.628
	Condition × subscale	-1.618	-6.888	3.652	-0.60	26	.553	.956
Not-Worrying	Intercept	21.964	13.102	30.827	4.86	26	<.001	
	Condition (Threat)	8.665	3.373	13.958	3.21	26	.004	
	MAIA subscale	-5.087	-14.028	3.854	-1.12	26	.275	.628
	Condition × subscale	-0.284	-5.623	5.056	-0.10	26	.918	.961
Self-Regulation	Intercept	22.051	13.083	31.018	4.82	26	<.001	

	Condition (Threat)	8.663	3.394	13.933	3.22	26	.003	
	MAIA subscale	3.565	-5.476	12.606	0.77	26	.447	.628
	Condition × subscale	1.298	-4.014	6.611	0.48	26	.636	.956
Attention Regulation	Intercept	22.082	13.024	31.140	4.78	26	<.001	
	Condition (Threat)	8.687	3.455	13.918	3.25	26	.003	
	MAIA subscale	1.228	-7.819	10.276	0.27	26	.792	.792
	Condition × subscale	2.084	-3.142	7.309	0.78	26	.442	.956
Emotional Awareness	Intercept	22.061	13.183	30.939	4.87	26	<.001	
	Condition (Threat)	8.677	3.466	13.888	3.26	26	.003	
	MAIA subscale	4.760	-3.993	13.512	1.07	26	.296	.628
	Condition × subscale	-2.373	-7.510	2.764	-0.91	26	.374	.956
Trusting	Intercept	22.082	13.071	31.093	4.80	26	<.001	
	Condition (Threat)	8.657	3.613	13.701	3.36	26	.002	
	MAIA subscale	-2.738	-11.905	6.430	-0.59	26	.563	.644
	Condition × subscale	4.243	-0.888	9.375	1.62	26	.117	.938

Table S3 - Interoceptive sensibility (MAIA subscales) and temporal reproduction accuracy. Results of eight separate linear mixed-effects models, each testing the interaction between subjective anxiety and one MAIA subscale on accuracy of reproduction (AR), controlling for target duration (N = 28). All models included by-subject random intercepts and random slopes for target duration. Benjamini–Hochberg FDR correction was applied separately across the eight subscale main effects and the eight interaction terms.

Subscale	*Term*	*b*	*95% CI low*	*95% CI high*	*t*	*df*	*p*	*p (FDR)*
Not-Distracting	Subjective anxiety	0.0020	-0.0068	0.0108	0.45	1848	.656	
	MAIA subscale	-0.0010	-0.0283	0.0263	-0.07	26	.945	.984
	Anxiety × subscale	-0.0072	-0.0167	0.0023	-1.48	1003	.140	.299
Noticing	Subjective anxiety	0.0007	-0.0079	0.0093	0.16	1657	.874	
	MAIA subscale	-0.0127	-0.0379	0.0126	-0.98	27	.334	.534
	Anxiety × subscale	-0.0025	-0.0099	0.0048	-0.68	1794	.498	.569
Body Listening	Subjective anxiety	0.0000	-0.0088	0.0089	0.01	1502	.995	
	MAIA subscale	-0.0138	-0.0390	0.0114	-1.07	26	.292	.534
	Anxiety × subscale	-0.0028	-0.0102	0.0047	-0.73	1919	.465	.569
Not-Worrying	Subjective anxiety	-0.0005	-0.0092	0.0082	-0.10	1766	.917	
	MAIA subscale	-0.0161	-0.0427	0.0106	-1.18	26	.247	.534
	Anxiety × subscale	0.0069	-0.0018	0.0156	1.55	1274	.121	.299
Self-Regulation	Subjective anxiety	0.0010	-0.0077	0.0097	0.23	1722	.819	
	MAIA subscale	-0.0042	-0.0303	0.0219	-0.31	27	.757	.984
	Anxiety × subscale	0.0020	-0.0061	0.0100	0.48	2111	.630	.630
Attention Regulation	Subjective anxiety	0.0001	-0.0086	0.0089	0.03	1707	.974	
	MAIA subscale	-0.0150	-0.0406	0.0106	-1.15	27	.260	.534

	Anxiety × subscale	0.0041	-0.0043	0.0124	0.96	1295	.338	.541
Emotional Awareness	Subjective anxiety	-0.0004	-0.0093	0.0084	-0.10	1189	.923	
	MAIA subscale	-0.0282	-0.0516	-0.0049	-2.37	27	.025	.203
	Anxiety × subscale	-0.0052	-0.0123	0.0019	-1.44	1628	.150	.299
Trusting	Subjective anxiety	-0.0021	-0.0115	0.0072	-0.45	985	.656	
	MAIA subscale	-0.0003	-0.0263	0.0258	-0.02	26	.984	.984
	Anxiety × subscale	0.0077	-0.0023	0.0178	1.51	1068	.133	.299

Table S4 – ROI-based whole-brain results in Safe condition.

For each task regressor (Encoding, Reproduction), the table lists brain regions of interest (ROIs) showing effects after false discovery rate (FDR) correction, along with MNI coordinates (X, Y, Z). ROIs are labeled using the Destrieux atlas nomenclature.

Regressor	ROI	p-value (FDR-corr)	X	Y	Z
Encoding	Right_S_temporal_transverse	1.12E-04	53	-18	7
Encoding	Right_Lat_Fis-ant-Horizont	1.49E-04	41	29	0
Encoding	Left_G_postcentral	1.68E-04	-47	-25	55
Encoding	Left_G_insular_short	1.79E-04	-38	10	-4
Encoding	Left_G_precentral	1.79E-04	-43	-9	53
Encoding	Left_Lat_Fis-post	1.79E-04	-41	-34	20
Encoding	Left_S_temporal_transverse	1.79E-04	-53	-23	5
Encoding	Right_G_insular_short	1.79E-04	38	12	-5
Encoding	Right_G_temp_sup-G_T_transv	1.91E-04	48	-14	7
Encoding	Left_S_postcentral	2.06E-04	-42	-33	46
Encoding	Left_S_precentral-sup-part	2.06E-04	-29	-10	56
Encoding	Right_S_circular_insula_sup	2.29E-04	37	9	9
Encoding	Right_Lat_Fis-ant-Vertical	2.30E-04	48	23	10
Encoding	Left_G_temp_sup-G_T_transv	2.81E-04	-49	-18	8
Encoding	Right_G_and_S_cingul-Mid-Ant	3.08E-04	7	17	35
Encoding	Right-VentralDC	3.28E-04	10	-15	-10
Encoding	Right_G_temp_sup-Plan_tempo	3.56E-04	61	-28	20
Encoding	Right_S_precentral-sup-part	3.64E-04	29	-9	56
Encoding	Right_S_precentral-inf-part	3.80E-04	45	7	30
Encoding	Left_G_temp_sup-Plan_tempo	3.97E-04	-59	-38	18
Encoding	Left_S_circular_insula_ant	3.97E-04	-31	22	-10
Encoding	Right_S_circular_insula_ant	3.97E-04	31	23	-10
Encoding	Left_S_circular_insula_sup	4.74E-04	-36	6	11
Encoding	Left-VentralDC	8.83E-04	-11	-16	-10
Encoding	Left_S_central	8.83E-04	-39	-19	46
Encoding	3rd-Ventricle	0.001015781	0	-11	-3
Encoding	Right_S_occipital_ant	0.001131438	46	-64	5
Encoding	Right_Lat_Fis-post	0.001306724	42	-27	20
Encoding	Left_G_and_S_cingul-Mid-Ant	0.00133926	-7	16	35
Encoding	Left_G_pariet_inf-Supramar	0.00133926	-60	-38	34
Encoding	Right_G_pariet_inf-Supramar	0.00133926	59	-29	36
Encoding	Right_G_front_inf-Opercular	0.00138827	51	14	9
Encoding	Left_S_precentral-inf-part	0.001439897	-45	4	30
Encoding	Right_S_oc-temp_med_and_Lingual	0.001977099	28	-48	-11
Encoding	Right_S_collat_transv_post	0.002044183	26	-79	-8
Encoding	Right_S_circular_insula_inf	0.00236074	42	-8	-7
Encoding	4th-Ventricle	0.002624254	-1	-46	-35
Encoding	Right_S_calcarine	0.003044779	18	-65	7
Encoding	Right_G_oc-temp_lat-fusifor	0.003362261	35	-48	-21

Encoding	Left_G_and_S_subcentral	0.003927355	-57	-11	15
Encoding	Left_S_circular_insula_inf	0.003927355	-41	-12	-5
Encoding	Right_G_precentral	0.0055216	42	-7	53
Encoding	Left_G_front_inf-Opercular	0.00569697	-51	12	10
Encoding	Left_S_oc_sup_and_transversal	0.005846729	-27	-84	21
Encoding	Left_G_front_sup	0.006677288	-9	27	45
Encoding	Left_S_occipital_ant	0.006677288	-41	-70	7
Encoding	Brain-Stem	0.006979516	0	-31	-34
Encoding	Left_S_calcarine	0.007492475	-15	-69	5
Encoding	Right_S_oc_sup_and_transversal	0.007810569	30	-79	23
Encoding	Right-Cerebellum-Cortex	0.007960548	24	-63	-37
Encoding	Left_S_intrapariet_and_P_trans	0.007960548	-32	-58	43
Encoding	Left_S_oc-temp_med_and_Lingual	0.007960548	-28	-48	-11
Encoding	Right_G_and_S_occipital_inf	0.008589228	43	-76	-12
Encoding	Left_G_Ins_lg_and_S_cent_ins	0.009472585	-38	-7	-2
Encoding	Right_G_temp_sup-Lateral	0.009538658	59	-4	-7
Encoding	Right_S_intrapariet_and_P_trans	0.010007873	30	-56	47
Encoding	Left_S_oc-temp_lat	0.011422317	-44	-50	-14
Encoding	Left_G_and_S_cingul-Mid-Post	0.012116818	-7	-11	41
Encoding	Right_G_front_sup	0.012116818	10	28	47
Encoding	Left_G_oc-temp_lat-fusifor	0.012684965	-36	-55	-19
Encoding	Right_G_occipital_middle	0.013018773	45	-79	15
Encoding	Right_G_Ins_lg_and_S_cent_ins	0.013487042	39	-4	-3
Encoding	Left-Cerebral-White-Matter	0.015573161	-29	-19	19
Encoding	Right-Cerebral-White-Matter	0.016139269	29	-18	19
Encoding	Right_S_front_middle	0.016139269	30	47	19
Encoding	Left-Thalamus-Proper	0.016591209	-11	-19	7
Encoding	Right-Cerebellum-White-Matter	0.016666406	19	-56	-33
Encoding	Left_S_collat_transv_post	0.016666406	-24	-81	-7
Encoding	Right_G_front_inf-Orbital	0.016666406	48	35	-11
Encoding	Right_G_oc-temp_med-Lingual	0.016666406	10	-67	-4
Encoding	Right_S_postcentral	0.016666406	38	-30	49
Encoding	Left_G_parietal_sup	0.018058063	-26	-61	59
Encoding	Left_G_occipital_middle	0.019679258	-39	-88	12
Encoding	Right_S_oc_middle_and_Lunatus	0.021516373	37	-79	10
Encoding	Left_G_subcallosal	0.022116239	-12	8	-13
Encoding	Left_Lat_Fis-ant-Vertical	0.022548854	-48	21	11
Encoding	Left_S_front_middle	0.022548854	-29	46	19
Encoding	Right_S_parieto_occipital	0.02320017	18	-66	28
Encoding	Left-Pallidum	0.023319827	-21	-5	-1
Encoding	Right_G_and_S_subcentral	0.024213403	57	-8	14
Encoding	Left_G_and_S_frontomargin	0.025047208	-24	61	-10
Encoding	Right_G_temp_sup-Plan_polar	0.02643951	36	9	-20
Encoding	Left_S_cingul-Marginalis	0.0272675	-13	-38	49
Encoding	Right_S_interm_prim-Jensen	0.0272675	52	-41	46

Encoding	Right_G_and_S_frontomargin	0.028250384	23	62	-11
Encoding	Left_S_pericallosal	0.029463771	-6	-4	17
Encoding	Left_G_and_S_occipital_inf	0.032742538	-39	-83	-10
Encoding	Left_G_cuneus	0.032742538	-4	-84	19
Encoding	Right_G_front_inf-Triangul	0.033877648	52	33	5
Encoding	Right_S_cingul-Marginalis	0.033877648	12	-37	50
Encoding	Left_G_oc-temp_med-Lingual	0.034017045	-10	-73	-6
Encoding	Left_S_collat_transv_ant	0.034144752	-41	-20	-30
Encoding	Right_G_cuneus	0.034529855	7	-80	21
Encoding	Right_G_orbital	0.035400994	29	38	-19
Encoding	Right_S_front_inf	0.035400994	40	27	23
Encoding	Left_G_temp_sup-Lateral	0.037197964	-58	-7	-7
Encoding	Right_G_front_middle	0.037484551	38	36	33
Encoding	Left_S_parieto_occipital	0.037707412	-15	-68	25
Encoding	Right_S_pericallosal	0.039204735	6	-7	19
Encoding	Left_Lat_Fis-ant-Horizont	0.041649949	-44	32	-4
Encoding	Right_G_and_S_cingul-Mid-Post	0.041724885	7	-8	43
Encoding	Right-Thalamus-Proper	0.042164304	11	-17	7
Encoding	Left_S_front_inf	0.043248718	-41	27	20
Encoding	Left_G_occipital_sup	0.045874087	-15	-92	29
Encoding	Right_G_parietal_sup	0.048167164	25	-60	62
Encoding	Right_S_oc-temp_lat	0.051203687	45	-47	-15
Encoding	Right_S_orbital_lateral	0.051203687	42	44	-3
Encoding	Right_G_and_S_cingul-Ant	0.05140624	7	41	8
Encoding	Left_S_oc_middle_and_Lunatus	0.051830673	-33	-87	8
Encoding	Left-Cerebellum-Cortex	0.061662636	-25	-63	-37
Encoding	Left-Accumbens-area	0.062628539	-9	11	-8
Encoding	Right_S_front_sup	0.063194325	23	25	45
Encoding	Right-Pallidum	0.063542057	21	-4	-2
Encoding	Left-Putamen	0.066971258	-26	1	0
Encoding	Right_G_occipital_sup	0.072094847	20	-89	31
Encoding	Right_G_oc-temp_med-Parahip	0.081905796	24	-13	-29
Encoding	Left_G_temp_sup-Plan_polar	0.082068253	-41	8	-20
Encoding	Left-Cerebellum-White-Matter	0.087284437	-20	-57	-33
Encoding	Right_S_orbital_med-olfact	0.087620894	12	27	-19
Encoding	Left_G_temporal_inf	0.090901297	-55	-40	-23
Encoding	Right_G_postcentral	0.095414045	47	-21	54
Encoding	Left_S_orbital_med-olfact	0.106457235	-13	25	-20
Encoding	Left_G_and_S_cingul-Ant	0.10832284	-8	41	6
Encoding	Left_G_front_inf-Orbital	0.10832284	-46	29	-10
Encoding	Right_G_temporal_inf	0.109036957	55	-35	-25
Encoding	Right_S_temporal_sup	0.123465132	51	-38	10
Encoding	Right_S_temporal_inf	0.148021166	55	-33	-16
Encoding	CC_Mid_Posterior	0.149399594	0	-18	22
Encoding	Right-Inf-Lat-Vent	0.149896646	31	-14	-18

Encoding	Left_G_oc-temp_med-Parahip	0.152656713	-23	-16	-26
Encoding	Right-Accumbens-area	0.187883613	8	12	-8
Encoding	Left_G_orbital	0.190214454	-31	35	-19
Encoding	Left_G_and_S_transv_frontopol	0.190950749	-17	67	-3
Encoding	Left_S_suborbital	0.194599481	-8	40	-14
Encoding	Left_S_temporal_inf	0.195969452	-55	-34	-16
Encoding	Right_Pole_occipital	0.200753273	20	-95	-3
Encoding	Right_Pole_temporal	0.200753273	32	9	-42
Encoding	Right_S_collat_transv_ant	0.200753273	41	-18	-29
Encoding	Right-Putamen	0.215584232	26	2	0
Encoding	Left_G_front_middle	0.224312761	-38	34	34
Encoding	Right-Hippocampus	0.250509542	26	-22	-14
Encoding	Left_G_rectus	0.253796632	-5	36	-23
Encoding	Right_S_orbital-H_Shaped	0.260645011	25	40	-16
Encoding	Right_G_temporal_middle	0.262516226	62	-23	-14
Encoding	Right_G_pariet_inf-Angular	0.27405392	50	-58	42
Encoding	Left_Pole_temporal	0.288611552	-33	6	-42
Encoding	Right_G_and_S_transv_frontopol	0.294950118	17	68	0
Encoding	Left-Caudate	0.29616819	-14	10	10
Encoding	CC_Anterior	0.309325262	0	25	5
Encoding	Left_S_orbital_lateral	0.315193236	-42	47	-5
Encoding	CC_Posterior	0.315510347	0	-35	17
Encoding	Left_Pole_occipital	0.320203064	-16	-101	-4
Encoding	Right-choroid-plexus	0.326683648	20	-24	8
Encoding	Left_S_temporal_sup	0.342055429	-51	-43	8
Encoding	Left_S_front_sup	0.380540893	-24	22	46
Encoding	Right-Caudate	0.404438213	13	11	9
Encoding	Left_S_orbital-H_Shaped	0.407049379	-26	40	-16
Encoding	Right_G_subcallosal	0.407049379	5	12	-13
Encoding	Right_S_suborbital	0.407049379	5	39	-18
Encoding	Right_S_central	0.426736755	37	-17	47
Encoding	Right_G_rectus	0.46210278	4	38	-24
Encoding	Left_G_temporal_middle	0.466753104	-63	-25	-13
Encoding	Right-Amygdala	0.468370604	23	-4	-20
Encoding	Right_G_cingul-Post-dorsal	0.487712395	3	-36	34
Encoding	Left-vessel	0.490942122	-29	-3	-9
Encoding	Left_G_cingul-Post-dorsal	0.492513717	-3	-38	32
Encoding	Left-Hippocampus	0.547745687	-26	-23	-14
Encoding	Right_G_cingul-Post-ventral	0.58848724	8	-48	7
Encoding	Right_G_precuneus	0.599442903	6	-60	47
Encoding	Right_G_and_S_paracentral	0.601439928	7	-35	69
Encoding	Left_G_cingul-Post-ventral	0.616785389	-8	-48	6
Encoding	CSF	0.618072703	0	-19	10
Encoding	Left_G_precuneus	0.62508709	-5	-60	45
Encoding	WM-hypointensities	0.677302288	-4	-14	19

Encoding	Optic-Chiasm	0.679478074	-1	1	-19
Encoding	Left_G_and_S_paracentral	0.679478074	-8	-36	68
Encoding	Left_S_interm_prim-Jensen	0.692195236	-52	-41	46
Encoding	Right_S_subparietal	0.714636079	9	-48	38
Encoding	Left-choroid-plexus	0.727621773	-22	-31	7
Encoding	CC_Central	0.825592886	0	-2	22
Encoding	CC_Mid_Anterior	0.825592886	0	13	19
Encoding	Left_G_pariet_inf-Angular	0.896346251	-44	-69	41
Encoding	Right-Lateral-Ventricle	0.896557336	14	-14	14
Encoding	Right-vessel	0.896557336	29	-2	-10
Encoding	Left_S_subparietal	0.900542531	-10	-49	38
Encoding	Left-Inf-Lat-Vent	0.916303008	-33	-20	-15
Encoding	Left-Lateral-Ventricle	0.932362418	-15	-17	14
Encoding	Left-Amygdala	0.932362418	-24	-5	-20
Encoding	Left_G_front_inf-Triangul	0.932362418	-52	31	4
Reproduction	Left_G_postcentral	3.71E-05	-47	-25	55
Reproduction	Left_G_precentral	3.71E-05	-43	-9	53
Reproduction	Right_Lat_Fis-ant-Horizont	3.71E-05	41	29	0
Reproduction	Right_S_temporal_transverse	3.71E-05	53	-18	7
Reproduction	Left_S_precentral-sup-part	5.14E-05	-29	-10	56
Reproduction	Right_G_insular_short	5.14E-05	38	12	-5
Reproduction	Right_S_circular_insula_sup	5.14E-05	37	9	9
Reproduction	Left_G_insular_short	5.32E-05	-38	10	-4
Reproduction	Left_S_postcentral	5.32E-05	-42	-33	46
Reproduction	Right_G_and_S_cingul-Mid-Ant	5.32E-05	7	17	35
Reproduction	Left_Lat_Fis-post	5.98E-05	-41	-34	20
Reproduction	Right_Lat_Fis-ant-Vertical	5.98E-05	48	23	10
Reproduction	Left_S_temporal_transverse	7.25E-05	-53	-23	5
Reproduction	Right_S_precentral-sup-part	7.25E-05	29	-9	56
Reproduction	Right_G_temp_sup-G_T_transv	9.74E-05	48	-14	7
Reproduction	Right_S_precentral-inf-part	9.79E-05	45	7	30
Reproduction	Right-VentralDC	1.42E-04	10	-15	-10
Reproduction	Left_S_circular_insula_sup	1.42E-04	-36	6	11
Reproduction	Right_S_circular_insula_ant	1.72E-04	31	23	-10
Reproduction	Left_G_temp_sup-G_T_transv	1.73E-04	-49	-18	8
Reproduction	Left_S_circular_insula_ant	1.80E-04	-31	22	-10
Reproduction	Left_G_temp_sup-Plan_tempo	1.89E-04	-59	-38	18
Reproduction	Right_G_temp_sup-Plan_tempo	1.89E-04	61	-28	20
Reproduction	Left_S_central	2.01E-04	-39	-19	46
Reproduction	3rd-Ventricle	2.04E-04	0	-11	-3
Reproduction	Right_G_front_inf-Opercular	2.04E-04	51	14	9
Reproduction	Left_G_and_S_cingul-Mid-Ant	2.70E-04	-7	16	35
Reproduction	Right_G_pariet_inf-Supramar	4.61E-04	59	-29	36
Reproduction	Left_G_pariet_inf-Supramar	4.69E-04	-60	-38	34
Reproduction	Left-VentralDC	4.86E-04	-11	-16	-10

Reproduction	Left_S_precentral-inf-part	7.02E-04	-45	4	30
Reproduction	Right_S_occipital_ant	0.001021335	46	-64	5
Reproduction	Right_Lat_Fis-post	0.001070884	42	-27	20
Reproduction	Left_G_front_inf-Opercular	0.001254676	-51	12	10
Reproduction	Right_S_collat_transv_post	0.001411238	26	-79	-8
Reproduction	Right_S_circular_insula_inf	0.001487767	42	-8	-7
Reproduction	Right_G_precentral	0.00162066	42	-7	53
Reproduction	Left_G_and_S_subcentral	0.001623467	-57	-11	15
Reproduction	Right_S_oc-temp_med_and_Lingual	0.001623467	28	-48	-11
Reproduction	4th-Ventricle	0.001931205	-1	-46	-35
Reproduction	Right_G_oc-temp_lat-fusifor	0.001958326	35	-48	-21
Reproduction	Left_G_front_sup	0.002279237	-9	27	45
Reproduction	Left_S_intrapariet_and_P_trans	0.002284499	-32	-58	43
Reproduction	Right_S_calcarine	0.002377098	18	-65	7
Reproduction	Left_S_circular_insula_inf	0.002576539	-41	-12	-5
Reproduction	Right_S_intrapariet_and_P_trans	0.002576539	30	-56	47
Reproduction	Right-Cerebellum-Cortex	0.002978448	24	-63	-37
Reproduction	Left_S_oc_sup_and_transversal	0.003248387	-27	-84	21
Reproduction	Left_S_occipital_ant	0.003472885	-41	-70	7
Reproduction	Right_S_front_middle	0.003587807	30	47	19
Reproduction	Right_S_oc_sup_and_transversal	0.003597498	30	-79	23
Reproduction	Right_G_front_sup	0.004175733	10	28	47
Reproduction	Right_G_and_S_occipital_inf	0.004197489	43	-76	-12
Reproduction	Brain-Stem	0.004211441	0	-31	-34
Reproduction	Left_G_and_S_cingul-Mid-Post	0.004511018	-7	-11	41
Reproduction	Left_S_calcarine	0.005409916	-15	-69	5
Reproduction	Right_G_temp_sup-Lateral	0.005409916	59	-4	-7
Reproduction	Left_G_Ins_lg_and_S_cent_ins	0.005415782	-38	-7	-2
Reproduction	Left-Thalamus-Proper	0.005882961	-11	-19	7
Reproduction	Left-Pallidum	0.00589484	-21	-5	-1
Reproduction	Left_Lat_Fis-ant-Vertical	0.00624067	-48	21	11
Reproduction	Left_S_oc-temp_med_and_Lingual	0.00624067	-28	-48	-11
Reproduction	Right-Cerebral-White-Matter	0.00717568	29	-18	19
Reproduction	Left_G_parietal_sup	0.00717568	-26	-61	59
Reproduction	Right_G_front_inf-Orbital	0.00717568	48	35	-11
Reproduction	Right_G_Ins_lg_and_S_cent_ins	0.00717568	39	-4	-3
Reproduction	Left-Cerebral-White-Matter	0.007202168	-29	-19	19
Reproduction	Right-Cerebellum-White-Matter	0.007584	19	-56	-33
Reproduction	Right_S_postcentral	0.007584	38	-30	49
Reproduction	Left_S_oc-temp_lat	0.007802344	-44	-50	-14
Reproduction	Right_G_occipital_middle	0.008161083	45	-79	15
Reproduction	Left_G_oc-temp_lat-fusifor	0.008523324	-36	-55	-19
Reproduction	Left_S_front_middle	0.009072275	-29	46	19
Reproduction	Right_S_interm_prim-Jensen	0.009072275	52	-41	46
Reproduction	Right_G_oc-temp_med-Lingual	0.00938259	10	-67	-4

Reproduction	Left_S_collat_transv_post	0.009916417	-24	-81	-7
Reproduction	Right_G_front_middle	0.011046513	38	36	33
Reproduction	Left_G_subcallosal	0.011646094	-12	8	-13
Reproduction	Right_G_and_S_subcentral	0.012252902	57	-8	14
Reproduction	Right_G_front_inf-Triangul	0.012545168	52	33	5
Reproduction	Right_S_front_inf	0.014690805	40	27	23
Reproduction	Left_G_occipital_middle	0.014780427	-39	-88	12
Reproduction	Right_G_and_S_frontomargin	0.014780427	23	62	-11
Reproduction	Left-Putamen	0.015710085	-26	1	0
Reproduction	Left_G_and_S_frontomargin	0.016299358	-24	61	-10
Reproduction	Right_S_oc_middle_and_Lunatus	0.016299358	37	-79	10
Reproduction	Right_G_temp_sup-Plan_polar	0.017673002	36	9	-20
Reproduction	Right-Thalamus-Proper	0.017728787	11	-17	7
Reproduction	Right_S_orbital_lateral	0.017728787	42	44	-3
Reproduction	Right_S_parieto_occipital	0.017728787	18	-66	28
Reproduction	Left_S_cingul-Marginalis	0.018281271	-13	-38	49
Reproduction	Left_S_front_inf	0.018281271	-41	27	20
Reproduction	Right_G_orbital	0.018281271	29	38	-19
Reproduction	Left_G_oc-temp_med-Lingual	0.018691752	-10	-73	-6
Reproduction	Left_G_and_S_occipital_inf	0.019128898	-39	-83	-10
Reproduction	Left_S_pericallosal	0.019128898	-6	-4	17
Reproduction	Left_G_cuneus	0.01964694	-4	-84	19
Reproduction	Right-Pallidum	0.021595604	21	-4	-2
Reproduction	Right_G_and_S_cingul-Mid-Post	0.021734628	7	-8	43
Reproduction	Right_S_pericallosal	0.022446671	6	-7	19
Reproduction	Left_Lat_Fis-ant-Horizont	0.023902602	-44	32	-4
Reproduction	Right_G_and_S_cingul-Ant	0.023902602	7	41	8
Reproduction	Right_G_cuneus	0.024141258	7	-80	21
Reproduction	Left-Accumbens-area	0.02569915	-9	11	-8
Reproduction	Left_G_temp_sup-Lateral	0.02571282	-58	-7	-7
Reproduction	Right_G_parietal_sup	0.025881218	25	-60	62
Reproduction	Left-Cerebellum-Cortex	0.026001936	-25	-63	-37
Reproduction	Right_S_cingul-Marginalis	0.026228965	12	-37	50
Reproduction	Right_S_orbital_med-olfact	0.026228965	12	27	-19
Reproduction	Right_S_oc-temp_lat	0.02997658	45	-47	-15
Reproduction	Left_G_occipital_sup	0.033161969	-15	-92	29
Reproduction	Left_S_collat_transv_ant	0.035529637	-41	-20	-30
Reproduction	Left_S_parieto_occipital	0.037650835	-15	-68	25
Reproduction	Right_S_front_sup	0.045408615	23	25	45
Reproduction	Left-Cerebellum-White-Matter	0.047840962	-20	-57	-33
Reproduction	Right-Accumbens-area	0.047840962	8	12	-8
Reproduction	Left_S_oc_middle_and_Lunatus	0.049027443	-33	-87	8
Reproduction	Right_G_occipital_sup	0.051704737	20	-89	31
Reproduction	Right_G_oc-temp_med-Parahip	0.068302707	24	-13	-29
Reproduction	Right-Putamen	0.068557418	26	2	0

Reproduction	Left_S_orbital_med-olfact	0.069554287	-13	25	-20
Reproduction	Right_G_temporal_inf	0.069554287	55	-35	-25
Reproduction	CC_Mid_Posterior	0.070982308	0	-18	22
Reproduction	Left_G_front_inf-Orbital	0.074327156	-46	29	-10
Reproduction	Left_G_temporal_inf	0.074327156	-55	-40	-23
Reproduction	Right_G_postcentral	0.074327156	47	-21	54
Reproduction	Right_S_temporal_sup	0.074327156	51	-38	10
Reproduction	Left_G_temp_sup-Plan_polar	0.074982987	-41	8	-20
Reproduction	Left_G_and_S_cingul-Ant	0.080356815	-8	41	6
Reproduction	Right_S_temporal_inf	0.102210166	55	-33	-16
Reproduction	Left-Caudate	0.106105312	-14	10	10
Reproduction	Right-Caudate	0.106465157	13	11	9
Reproduction	Left_G_front_middle	0.130926077	-38	34	34
Reproduction	Left_G_orbital	0.133206958	-31	35	-19
Reproduction	Left_G_oc-temp_med-Parahip	0.14160848	-23	-16	-26
Reproduction	Right-Inf-Lat-Vent	0.148337044	31	-14	-18
Reproduction	Right_G_pariet_inf-Angular	0.159989381	50	-58	42
Reproduction	Right_S_orbital-H_Shaped	0.164475488	25	40	-16
Reproduction	Left_S_suborbital	0.167427328	-8	40	-14
Reproduction	Right_S_collat_transv_ant	0.170265699	41	-18	-29
Reproduction	Right-choroid-plexus	0.171063348	20	-24	8
Reproduction	Right_G_temporal_middle	0.176701656	62	-23	-14
Reproduction	Left_S_temporal_inf	0.177100674	-55	-34	-16
Reproduction	CC_Posterior	0.179564527	0	-35	17
Reproduction	Right_Pole_occipital	0.184257681	20	-95	-3
Reproduction	CC_Anterior	0.191026508	0	25	5
Reproduction	Left_G_rectus	0.193518004	-5	36	-23
Reproduction	Right_G_subcallosal	0.199216615	5	12	-13
Reproduction	Left_S_orbital_lateral	0.208087157	-42	47	-5
Reproduction	Right_Pole_temporal	0.214341799	32	9	-42
Reproduction	Right-Hippocampus	0.218960691	26	-22	-14
Reproduction	Left_G_and_S_transv_frontopol	0.231160222	-17	67	-3
Reproduction	Right_G_and_S_transv_frontopol	0.231160222	17	68	0
Reproduction	Left-vessel	0.255449025	-29	-3	-9
Reproduction	Left_S_orbital-H_Shaped	0.256099578	-26	40	-16
Reproduction	Right_S_suborbital	0.256653959	5	39	-18
Reproduction	Left_Pole_temporal	0.283729832	-33	6	-42
Reproduction	Left_S_temporal_sup	0.331066296	-51	-43	8
Reproduction	Right_G_rectus	0.331066296	4	38	-24
Reproduction	Left_S_front_sup	0.333690669	-24	22	46
Reproduction	Left_Pole_occipital	0.349374014	-16	-101	-4
Reproduction	CSF	0.365609908	0	-19	10
Reproduction	Right_S_central	0.392703478	37	-17	47
Reproduction	Right_G_cingul-Post-dorsal	0.413037276	3	-36	34
Reproduction	Left_G_temporal_middle	0.418944726	-63	-25	-13

Reproduction	Right-Amygdala	0.436143215	23	-4	-20
Reproduction	Left_G_cingul-Post-dorsal	0.497339423	-3	-38	32
Reproduction	Right-Lateral-Ventricle	0.51577808	14	-14	14
Reproduction	Left-Hippocampus	0.543463471	-26	-23	-14
Reproduction	Right_G_cingul-Post-ventral	0.544209536	8	-48	7
Reproduction	Right_G_and_S_paracentral	0.569808836	7	-35	69
Reproduction	Right_G_precuneus	0.572800297	6	-60	47
Reproduction	Left-choroid-plexus	0.583266937	-22	-31	7
Reproduction	Left_G_and_S_paracentral	0.601676971	-8	-36	68
Reproduction	Left_G_cingul-Post-ventral	0.601676971	-8	-48	6
Reproduction	Right_S_subparietal	0.625963085	9	-48	38
Reproduction	Optic-Chiasm	0.665612575	-1	1	-19
Reproduction	Left_G_precuneus	0.665779513	-5	-60	45
Reproduction	Left_G_front_inf-Triangul	0.674486684	-52	31	4
Reproduction	Left-Lateral-Ventricle	0.696322343	-15	-17	14
Reproduction	Left_S_interm_prim-Jensen	0.696598876	-52	-41	46
Reproduction	CC_Central	0.766980718	0	-2	22
Reproduction	CC_Mid_Anterior	0.881032794	0	13	19
Reproduction	Right-vessel	0.897560217	29	-2	-10
Reproduction	Left_S_subparietal	0.897560217	-10	-49	38
Reproduction	Left_G_pariet_inf-Angular	0.912810929	-44	-69	41
Reproduction	Left-Amygdala	0.93160128	-24	-5	-20
Reproduction	Left-Inf-Lat-Vent	0.936519323	-33	-20	-15
Reproduction	WM-hypointensities	0.999964623	-4	-14	19

Table S5 – ROI-based whole-brain results in Threat condition.

For each task regressor (Encoding, Reproduction, Scream), the table lists brain regions of interest (ROIs) showing effects after false discovery rate (FDR) correction, along with MNI coordinates (X, Y, Z). ROIs are labeled using the Destrieux atlas nomenclature.

Regressor	ROI	p-value (FDR corr)	X	Y	Z
Encoding	Left_G_postcentral	1.07E-06	-47	-25	55
Encoding	Left_S_precentral-sup-part	1.07E-06	-29	-10	56
Encoding	Left_G_precentral	1.33E-06	-43	-9	53
Encoding	Left_S_postcentral	1.90E-06	-42	-33	46
Encoding	Left_G_temp_sup-G_T_transv	2.33E-06	-49	-18	8
Encoding	Right_G_and_S_cingul-Mid-Ant	2.33E-06	7	17	35
Encoding	Right_S_temporal_transverse	2.33E-06	53	-18	7
Encoding	Left_Lat_Fis-post	2.77E-06	-41	-34	20
Encoding	Right_Lat_Fis-ant-Horizont	7.51E-06	41	29	0
Encoding	Left_G_and_S_cingul-Mid-Ant	8.18E-06	-7	16	35
Encoding	Right_G_temp_sup-G_T_transv	8.39E-06	48	-14	7
Encoding	Left_S_circular_insula_sup	9.05E-06	-36	6	11
Encoding	Left_G_insular_short	1.04E-05	-38	10	-4
Encoding	Left_S_temporal_transverse	1.61E-05	-53	-23	5
Encoding	Right_G_insular_short	3.78E-05	38	12	-5
Encoding	Right_S_circular_insula_sup	4.45E-05	37	9	9
Encoding	Right_S_precentral-sup-part	5.39E-05	29	-9	56
Encoding	Left_S_central	1.27E-04	-39	-19	46
Encoding	Right_S_circular_insula_ant	1.27E-04	31	23	-10
Encoding	Left_G_temp_sup-Plan_tempo	1.38E-04	-59	-38	18
Encoding	Right_G_temp_sup-Plan_tempo	1.51E-04	61	-28	20
Encoding	Right_Lat_Fis-ant-Vertical	1.51E-04	48	23	10
Encoding	Right_S_precentral-inf-part	1.93E-04	45	7	30
Encoding	Left_S_circular_insula_inf	9.96E-04	-41	-12	-5
Encoding	Left_S_circular_insula_ant	0.001139919	-31	22	-10
Encoding	Left_S_precentral-inf-part	0.001139919	-45	4	30
Encoding	Left_G_pariet_inf-Supramar	0.001187888	-60	-38	34
Encoding	Right_S_oc_sup_and_transversal	0.001549711	30	-79	23
Encoding	Right_Lat_Fis-post	0.001634552	42	-27	20
Encoding	Left_G_and_S_occipital_inf	0.00227901	-39	-83	-10
Encoding	Left_G_and_S_subcentral	0.002362815	-57	-11	15
Encoding	Right_G_oc-temp_lat-fusifor	0.002362815	35	-48	-21
Encoding	3rd-Ventricle	0.002478053	0	-11	-3
Encoding	Right_S_oc_middle_and_Lunatus	0.002478053	37	-79	10
Encoding	Right_G_precentral	0.00251663	42	-7	53
Encoding	Left-Cerebral-White-Matter	0.002551632	-29	-19	19
Encoding	Right_G_front_inf-Opercular	0.002551632	51	14	9
Encoding	Left_G_front_inf-Opercular	0.002837262	-51	12	10
Encoding	Left_S_oc_sup_and_transversal	0.003512248	-27	-84	21

Encoding	Left_S_intrapariet_and_P_trans	0.003834088	-32	-58	43
Encoding	Left-Thalamus-Proper	0.004103602	-11	-19	7
Encoding	Right_S_oc-temp_med_and_Lingual	0.00418664	28	-48	-11
Encoding	Brain-Stem	0.004539704	0	-31	-34
Encoding	Left-VentralDC	0.004539704	-11	-16	-10
Encoding	Right-VentralDC	0.004539704	10	-15	-10
Encoding	Left_G_and_S_cingul-Mid-Post	0.004539704	-7	-11	41
Encoding	Left_G_Ins_lg_and_S_cent_ins	0.004539704	-38	-7	-2
Encoding	Left_S_occipital_ant	0.004539704	-41	-70	7
Encoding	Right_S_circular_insula_inf	0.004539704	42	-8	-7
Encoding	Right_S_occipital_ant	0.004539704	46	-64	5
Encoding	4th-Ventricle	0.005385533	-1	-46	-35
Encoding	Right_G_pariet_inf-Supramar	0.005850942	59	-29	36
Encoding	Right-Cerebral-White-Matter	0.006426403	29	-18	19
Encoding	Right_G_temp_sup-Lateral	0.007217212	59	-4	-7
Encoding	Left_Lat_Fis-ant-Vertical	0.007549368	-48	21	11
Encoding	Right_G_and_S_occipital_inf	0.007737632	43	-76	-12
Encoding	Left_G_parietal_sup	0.007810865	-26	-61	59
Encoding	Right_S_front_middle	0.008038319	30	47	19
Encoding	Left_Lat_Fis-ant-Horizont	0.00868886	-44	32	-4
Encoding	Right_G_occipital_middle	0.008769186	45	-79	15
Encoding	Right_S_pericallosal	0.008769186	6	-7	19
Encoding	Left_S_pericallosal	0.008774273	-6	-4	17
Encoding	Right_S_collat_transv_post	0.008774273	26	-79	-8
Encoding	Left_S_oc_middle_and_Lunatus	0.009847411	-33	-87	8
Encoding	Right_S_calcarine	0.009847411	18	-65	7
Encoding	Right_G_and_S_subcentral	0.010386198	57	-8	14
Encoding	Left_S_calcarine	0.010507302	-15	-69	5
Encoding	Left_G_oc-temp_lat-fusifor	0.010512086	-36	-55	-19
Encoding	Right_S_orbital_med-olfact	0.012359768	12	27	-19
Encoding	Right-Cerebellum-White-Matter	0.013188827	19	-56	-33
Encoding	Right_S_intrapariet_and_P_trans	0.01333893	30	-56	47
Encoding	Left_G_front_sup	0.013344178	-9	27	45
Encoding	Left_S_collat_transv_post	0.013797223	-24	-81	-7
Encoding	Right_G_front_sup	0.016155085	10	28	47
Encoding	Left_S_oc-temp_med_and_Lingual	0.018997896	-28	-48	-11
Encoding	Left_S_cingul-Marginalis	0.019478738	-13	-38	49
Encoding	Right_G_temp_sup-Plan_polar	0.019721314	36	9	-20
Encoding	Right-Thalamus-Proper	0.020720223	11	-17	7
Encoding	Right_G_oc-temp_med-Lingual	0.020720223	10	-67	-4
Encoding	Left_S_interm_prim-Jensen	0.024383997	-52	-41	46
Encoding	Left_G_occipital_middle	0.024458449	-39	-88	12
Encoding	Right-Cerebellum-Cortex	0.024917294	24	-63	-37
Encoding	Right_G_and_S_cingul-Mid-Post	0.025300103	7	-8	43
Encoding	Right_G_occipital_sup	0.025463982	20	-89	31

Encoding	Right_G_and_S_cingul-Ant	0.02552898	7	41	8
Encoding	Left_G_temp_sup-Lateral	0.026327586	-58	-7	-7
Encoding	Left_S_oc-temp_lat	0.026363461	-44	-50	-14
Encoding	Right_S_postcentral	0.029915357	38	-30	49
Encoding	Left_G_oc-temp_med-Lingual	0.031421225	-10	-73	-6
Encoding	Right_S_front_inf	0.034317495	40	27	23
Encoding	Left_G_occipital_sup	0.037199188	-15	-92	29
Encoding	Left_G_pariet_inf-Angular	0.038172315	-44	-69	41
Encoding	Left-Accumbens-area	0.03861476	-9	11	-8
Encoding	Right_S_oc-temp_lat	0.052999668	45	-47	-15
Encoding	Right_G_Ins_lg_and_S_cent_ins	0.063359269	39	-4	-3
Encoding	Left-Pallidum	0.07459007	-21	-5	-1
Encoding	Right_S_cingul-Marginalis	0.076507254	12	-37	50
Encoding	Right_S_parieto_occipital	0.076507254	18	-66	28
Encoding	Right_G_front_inf-Triangul	0.079803916	52	33	5
Encoding	Right-Accumbens-area	0.081844852	8	12	-8
Encoding	Right_S_orbital-H_Shaped	0.088899897	25	40	-16
Encoding	CC_Central	0.09383724	0	-2	22
Encoding	Left_G_cuneus	0.09427347	-4	-84	19
Encoding	Right_G_front_inf-Orbital	0.100378045	48	35	-11
Encoding	Right_G_cuneus	0.106955267	7	-80	21
Encoding	Left_S_subparietal	0.112911179	-10	-49	38
Encoding	Left_Pole_temporal	0.119296399	-33	6	-42
Encoding	Left_G_temp_sup-Plan_polar	0.120888889	-41	8	-20
Encoding	Left_S_front_middle	0.122863091	-29	46	19
Encoding	Right_S_collat_transv_ant	0.123933709	41	-18	-29
Encoding	CC_Mid_Posterior	0.140771621	0	-18	22
Encoding	Left-Caudate	0.141539827	-14	10	10
Encoding	Right_S_orbital_lateral	0.150575412	42	44	-3
Encoding	Left_G_and_S_cingul-Ant	0.158409164	-8	41	6
Encoding	Right_G_oc-temp_med-Parahip	0.160488024	24	-13	-29
Encoding	Left-Cerebellum-Cortex	0.167938932	-25	-63	-37
Encoding	Right_G_orbital	0.172872298	29	38	-19
Encoding	Right-vessel	0.173396331	29	-2	-10
Encoding	Left-Cerebellum-White-Matter	0.174542816	-20	-57	-33
Encoding	Left_S_front_inf	0.195654284	-41	27	20
Encoding	Left_S_orbital_med-olfact	0.195654284	-13	25	-20
Encoding	Left_S_orbital-H_Shaped	0.195654284	-26	40	-16
Encoding	Right_G_parietal_sup	0.195654284	25	-60	62
Encoding	Left_S_parieto_occipital	0.22714459	-15	-68	25
Encoding	CSF	0.231119274	0	-19	10
Encoding	Right-Caudate	0.234096889	13	11	9
Encoding	CC_Anterior	0.257838777	0	25	5
Encoding	Left_G_subcallosal	0.268373417	-12	8	-13
Encoding	Right-Pallidum	0.274650202	21	-4	-2

Encoding	Right_G_postcentral	0.304126651	47	-21	54
Encoding	Left_G_and_S_transv_frontopol	0.308373607	-17	67	-3
Encoding	Right_G_front_middle	0.308373607	38	36	33
Encoding	Right-choroid-plexus	0.312300891	20	-24	8
Encoding	Left_G_oc-temp_med-Parahip	0.312300891	-23	-16	-26
Encoding	Right_Pole_occipital	0.312300891	20	-95	-3
Encoding	CC_Posterior	0.317781225	0	-35	17
Encoding	Right_S_interm_prim-Jensen	0.338371435	52	-41	46
Encoding	CC_Mid_Anterior	0.342899896	0	13	19
Encoding	Right-Lateral-Ventricle	0.347294132	14	-14	14
Encoding	Left-Putamen	0.34988049	-26	1	0
Encoding	Right_G_temporal_inf	0.364246379	55	-35	-25
Encoding	Left_Pole_occipital	0.391565859	-16	-101	-4
Encoding	Right-Hippocampus	0.393260054	26	-22	-14
Encoding	Left_G_precuneus	0.393260054	-5	-60	45
Encoding	Right_G_and_S_transv_frontopol	0.393260054	17	68	0
Encoding	Right_Pole_temporal	0.405079187	32	9	-42
Encoding	Right_S_temporal_sup	0.428853471	51	-38	10
Encoding	Right_S_temporal_inf	0.434488232	55	-33	-16
Encoding	Right_G_subcallosal	0.482535036	5	12	-13
Encoding	Left_G_and_S_frontomargin	0.506846452	-24	61	-10
Encoding	Right_G_cingul-Post-ventral	0.5085953	8	-48	7
Encoding	Left_S_temporal_sup	0.517962012	-51	-43	8
Encoding	Right_S_subparietal	0.533805552	9	-48	38
Encoding	Left-Lateral-Ventricle	0.546047679	-15	-17	14
Encoding	Left-vessel	0.546047679	-29	-3	-9
Encoding	Left_G_temporal_middle	0.546047679	-63	-25	-13
Encoding	Left-Hippocampus	0.596553095	-26	-23	-14
Encoding	Right_S_front_sup	0.632227598	23	25	45
Encoding	Left_G_rectus	0.647703198	-5	36	-23
Encoding	Right_G_precuneus	0.647703198	6	-60	47
Encoding	Right_G_and_S_frontomargin	0.661483831	23	62	-11
Encoding	Left_S_collat_transv_ant	0.678037892	-41	-20	-30
Encoding	Left-choroid-plexus	0.680479049	-22	-31	7
Encoding	Optic-Chiasm	0.725326914	-1	1	-19
Encoding	Left-Inf-Lat-Vent	0.728799351	-33	-20	-15
Encoding	Left_G_orbital	0.732767168	-31	35	-19
Encoding	Right_G_pariet_inf-Angular	0.741644984	50	-58	42
Encoding	Left_G_cingul-Post-ventral	0.774040782	-8	-48	6
Encoding	Left_S_suborbital	0.774040782	-8	40	-14
Encoding	Right_G_cingul-Post-dorsal	0.774040782	3	-36	34
Encoding	Left_S_orbital_lateral	0.785328972	-42	47	-5
Encoding	Left_G_and_S_paracentral	0.79940793	-8	-36	68
Encoding	Right-Putamen	0.801034856	26	2	0
Encoding	Left_S_front_sup	0.80337867	-24	22	46

Encoding	Right_G_rectus	0.80337867	4	38	-24
Encoding	Right_S_suborbital	0.80337867	5	39	-18
Encoding	Left_G_front_inf-Triangul	0.826948079	-52	31	4
Encoding	Left_G_cingul-Post-dorsal	0.849431819	-3	-38	32
Encoding	Left_G_front_middle	0.85407446	-38	34	34
Encoding	WM-hypointensities	0.881043542	-4	-14	19
Encoding	Right-Inf-Lat-Vent	0.885407401	31	-14	-18
Encoding	Left_G_front_inf-Orbital	0.893317463	-46	29	-10
Encoding	Right_S_central	0.895253948	37	-17	47
Encoding	Left_S_temporal_inf	0.9067558	-55	-34	-16
Encoding	Left-Amygdala	0.944014071	-24	-5	-20
Encoding	Right_G_and_S_paracentral	0.971434769	7	-35	69
Encoding	Right-Amygdala	0.976440732	23	-4	-20
Encoding	Right_G_temporal_middle	0.989863949	62	-23	-14
Encoding	Left_G_temporal_inf	0.995857337	-55	-40	-23
Reproduction	Left_G_postcentral	4.24E-08	-47	-25	55
Reproduction	Left_G_precentral	6.60E-08	-43	-9	53
Reproduction	Left_S_postcentral	7.37E-08	-42	-33	46
Reproduction	Right_G_and_S_cingul-Mid-Ant	2.47E-07	7	17	35
Reproduction	Left_G_temp_sup-G_T_transv	8.31E-07	-49	-18	8
Reproduction	Left_G_and_S_cingul-Mid-Ant	8.32E-07	-7	16	35
Reproduction	Right_S_temporal_transverse	9.34E-07	53	-18	7
Reproduction	Right_Lat_Fis-ant-Horizont	1.07E-06	41	29	0
Reproduction	Left_Lat_Fis-post	1.56E-06	-41	-34	20
Reproduction	Left_S_circular_insula_sup	1.56E-06	-36	6	11
Reproduction	Left_S_precentral-sup-part	2.13E-06	-29	-10	56
Reproduction	Left_G_insular_short	3.22E-06	-38	10	-4
Reproduction	Right_G_temp_sup-G_T_transv	3.32E-06	48	-14	7
Reproduction	Right_G_insular_short	3.91E-06	38	12	-5
Reproduction	Right_S_circular_insula_sup	3.91E-06	37	9	9
Reproduction	Left_S_temporal_transverse	4.44E-06	-53	-23	5
Reproduction	Right_S_precentral-sup-part	9.84E-06	29	-9	56
Reproduction	Right_Lat_Fis-ant-Vertical	1.71E-05	48	23	10
Reproduction	Right_S_circular_insula_ant	1.75E-05	31	23	-10
Reproduction	Left_S_central	2.30E-05	-39	-19	46
Reproduction	Right_S_precentral-inf-part	4.84E-05	45	7	30
Reproduction	Left_G_temp_sup-Plan_tempo	5.14E-05	-59	-38	18
Reproduction	Right_G_temp_sup-Plan_tempo	6.01E-05	61	-28	20
Reproduction	Left_G_pariet_inf-Supramar	2.15E-04	-60	-38	34
Reproduction	Right_G_front_inf-Opercular	3.40E-04	51	14	9
Reproduction	Left_G_front_inf-Opercular	4.32E-04	-51	12	10
Reproduction	Right_G_precentral	4.33E-04	42	-7	53
Reproduction	Left_G_and_S_subcentral	4.88E-04	-57	-11	15
Reproduction	3rd-Ventricle	5.08E-04	0	-11	-3
Reproduction	Left_S_precentral-inf-part	5.87E-04	-45	4	30

Reproduction	Left_G_and_S_occipital_inf	5.92E-04	-39	-83	-10
Reproduction	Right_Lat_Fis-post	7.26E-04	42	-27	20
Reproduction	Left-Cerebral-White-Matter	0.001155739	-29	-19	19
Reproduction	Right-VentralDC	0.001155739	10	-15	-10
Reproduction	Left_G_and_S_cingul-Mid-Post	0.001155739	-7	-11	41
Reproduction	Left_Lat_Fis-ant-Vertical	0.001155739	-48	21	11
Reproduction	Left_S_circular_insula_ant	0.001155739	-31	22	-10
Reproduction	Left_S_circular_insula_inf	0.001155739	-41	-12	-5
Reproduction	Right_G_oc-temp_lat-fusifor	0.001155739	35	-48	-21
Reproduction	Right_S_oc_sup_and_transversal	0.001166856	30	-79	23
Reproduction	Right_G_pariet_inf-Supramar	0.001184211	59	-29	36
Reproduction	Left_S_intrapariet_and_P_trans	0.001389954	-32	-58	43
Reproduction	Right_S_front_middle	0.00187554	30	47	19
Reproduction	Right_S_oc-temp_med_and_Lingual	0.00204088	28	-48	-11
Reproduction	Left-VentralDC	0.002052955	-11	-16	-10
Reproduction	Left-Thalamus-Proper	0.002178745	-11	-19	7
Reproduction	Right_S_occipital_ant	0.002178745	46	-64	5
Reproduction	Left_G_Ins_lg_and_S_cent_ins	0.002193973	-38	-7	-2
Reproduction	Left_S_occipital_ant	0.002193973	-41	-70	7
Reproduction	Right_G_front_sup	0.002193973	10	28	47
Reproduction	Left_G_parietal_sup	0.002202096	-26	-61	59
Reproduction	Right-Cerebral-White-Matter	0.002567803	29	-18	19
Reproduction	Left_G_front_sup	0.002567803	-9	27	45
Reproduction	Brain-Stem	0.002589113	0	-31	-34
Reproduction	Right_S_oc_middle_and_Lunatus	0.002589113	37	-79	10
Reproduction	Right_S_orbital_med-olfact	0.002589113	12	27	-19
Reproduction	Left_S_oc_sup_and_transversal	0.002720782	-27	-84	21
Reproduction	Left_Lat_Fis-ant-Horizont	0.002745965	-44	32	-4
Reproduction	Right_S_intrapariet_and_P_trans	0.002955194	30	-56	47
Reproduction	Right_G_and_S_occipital_inf	0.003069039	43	-76	-12
Reproduction	Right_G_and_S_subcentral	0.003143985	57	-8	14
Reproduction	Right_S_pericallosal	0.003143985	6	-7	19
Reproduction	Right_S_circular_insula_inf	0.003526614	42	-8	-7
Reproduction	Right_G_temp_sup-Lateral	0.003706784	59	-4	-7
Reproduction	Left_G_oc-temp_lat-fusifor	0.00413778	-36	-55	-19
Reproduction	Left_S_pericallosal	0.004995177	-6	-4	17
Reproduction	Right_S_calcarine	0.005041484	18	-65	7
Reproduction	Left_S_calcarine	0.005536016	-15	-69	5
Reproduction	Right_S_postcentral	0.005838492	38	-30	49
Reproduction	Right_G_occipital_middle	0.005900773	45	-79	15
Reproduction	Right-Cerebellum-White-Matter	0.006534661	19	-56	-33
Reproduction	Right_G_and_S_cingul-Mid-Post	0.006534661	7	-8	43
Reproduction	Right_G_and_S_cingul-Ant	0.006933909	7	41	8
Reproduction	Right-Thalamus-Proper	0.007023048	11	-17	7
Reproduction	Left_S_oc-temp_lat	0.007023048	-44	-50	-14

Reproduction	Right_S_collat_transv_post	0.008041152	26	-79	-8
Reproduction	Right_G_oc-temp_med-Lingual	0.008398175	10	-67	-4
Reproduction	Left_S_cingul-Marginalis	0.009387	-13	-38	49
Reproduction	Left-Accumbens-area	0.011308737	-9	11	-8
Reproduction	Left_S_oc-temp_med_and_Lingual	0.011308737	-28	-48	-11
Reproduction	Left_G_oc-temp_med-Lingual	0.011748235	-10	-73	-6
Reproduction	Left_S_oc_middle_and_Lunatus	0.011748235	-33	-87	8
Reproduction	Right-Cerebellum-Cortex	0.011784705	24	-63	-37
Reproduction	Left_S_collat_transv_post	0.011873099	-24	-81	-7
Reproduction	Right_S_front_inf	0.011881397	40	27	23
Reproduction	Left_G_temp_sup-Lateral	0.012154834	-58	-7	-7
Reproduction	Right_G_temp_sup-Plan_polar	0.013208389	36	9	-20
Reproduction	Right-Accumbens-area	0.014602834	8	12	-8
Reproduction	Right_G_front_inf-Triangul	0.016041904	52	33	5
Reproduction	Left_G_occipital_middle	0.018193644	-39	-88	12
Reproduction	Right_S_oc-temp_lat	0.021824316	45	-47	-15
Reproduction	Right_G_Ins_lg_and_S_cent_ins	0.024582245	39	-4	-3
Reproduction	Right_S_orbital-H_Shaped	0.026134469	25	40	-16
Reproduction	Left-Pallidum	0.02652497	-21	-5	-1
Reproduction	Right_G_occipital_sup	0.028359188	20	-89	31
Reproduction	4th-Ventricle	0.029187045	-1	-46	-35
Reproduction	Right_G_front_inf-Orbital	0.029187045	48	35	-11
Reproduction	Right_S_cingul-Marginalis	0.030671429	12	-37	50
Reproduction	Right_S_orbital_lateral	0.034300894	42	44	-3
Reproduction	Left-Caudate	0.03476033	-14	10	10
Reproduction	Right_G_orbital	0.043136934	29	38	-19
Reproduction	Right_S_collat_transv_ant	0.043136934	41	-18	-29
Reproduction	Left_G_occipital_sup	0.043203848	-15	-92	29
Reproduction	CC_Central	0.044295406	0	-2	22
Reproduction	Right-Caudate	0.045441966	13	11	9
Reproduction	Left_G_cuneus	0.048475214	-4	-84	19
Reproduction	Right_S_parieto_occipital	0.053233733	18	-66	28
Reproduction	CC_Mid_Posterior	0.054533444	0	-18	22
Reproduction	Left_G_temp_sup-Plan_polar	0.056869204	-41	8	-20
Reproduction	Right_G_cuneus	0.057627913	7	-80	21
Reproduction	Right_G_front_middle	0.064539966	38	36	33
Reproduction	Left_S_front_middle	0.066038649	-29	46	19
Reproduction	Left-Cerebellum-White-Matter	0.070462254	-20	-57	-33
Reproduction	Left-Cerebellum-Cortex	0.071300205	-25	-63	-37
Reproduction	Left_S_orbital_med-olfact	0.071300205	-13	25	-20
Reproduction	Left_G_and_S_cingul-Ant	0.075348525	-8	41	6
Reproduction	Left_Pole_temporal	0.075348525	-33	6	-42
Reproduction	Right_G_parietal_sup	0.078346083	25	-60	62
Reproduction	Right_S_interm_prim-Jensen	0.080078313	52	-41	46
Reproduction	Left-Putamen	0.082113686	-26	1	0

Reproduction	Right-Pallidum	0.082113686	21	-4	-2
Reproduction	Right_G_oc-temp_med-Parahip	0.082113686	24	-13	-29
Reproduction	CSF	0.083741287	0	-19	10
Reproduction	Left_G_subcallosal	0.083741287	-12	8	-13
Reproduction	Left_S_front_inf	0.086654994	-41	27	20
Reproduction	Left_S_orbital-H_Shaped	0.098982881	-26	40	-16
Reproduction	Left_G_pariet_inf-Angular	0.124913753	-44	-69	41
Reproduction	Right-Lateral-Ventricle	0.156541269	14	-14	14
Reproduction	Right-choroid-plexus	0.174194163	20	-24	8
Reproduction	Right_G_temporal_inf	0.17479143	55	-35	-25
Reproduction	Right_S_temporal_sup	0.178144264	51	-38	10
Reproduction	Left_S_parieto_occipital	0.17837863	-15	-68	25
Reproduction	CC_Anterior	0.187685027	0	25	5
Reproduction	CC_Posterior	0.198270687	0	-35	17
Reproduction	Right_G_postcentral	0.201917908	47	-21	54
Reproduction	Right_G_subcallosal	0.213422996	5	12	-13
Reproduction	Right-Hippocampus	0.216639588	26	-22	-14
Reproduction	Left_G_oc-temp_med-Parahip	0.216639588	-23	-16	-26
Reproduction	Left_S_subparietal	0.216639588	-10	-49	38
Reproduction	Left_S_interm_prim-Jensen	0.222902128	-52	-41	46
Reproduction	Right_Pole_occipital	0.248977796	20	-95	-3
Reproduction	Right_S_temporal_inf	0.257661592	55	-33	-16
Reproduction	Right_Pole_temporal	0.261728706	32	9	-42
Reproduction	Left_G_and_S_transv_frontopol	0.317920384	-17	67	-3
Reproduction	CC_Mid_Anterior	0.321402204	0	13	19
Reproduction	Left_Pole_occipital	0.365787263	-16	-101	-4
Reproduction	Right-Putamen	0.390392279	26	2	0
Reproduction	Left_G_and_S_frontomargin	0.399128798	-24	61	-10
Reproduction	Right-vessel	0.40081919	29	-2	-10
Reproduction	Right_S_front_sup	0.402285553	23	25	45
Reproduction	Left-choroid-plexus	0.415289483	-22	-31	7
Reproduction	Left-Hippocampus	0.417913583	-26	-23	-14
Reproduction	Right_G_and_S_frontomargin	0.417913583	23	62	-11
Reproduction	Left_G_front_inf-Orbital	0.46306206	-46	29	-10
Reproduction	Left_G_precuneus	0.463243673	-5	-60	45
Reproduction	Left_G_rectus	0.465261035	-5	36	-23
Reproduction	Left-Lateral-Ventricle	0.468058176	-15	-17	14
Reproduction	Left_G_and_S_paracentral	0.504634954	-8	-36	68
Reproduction	Right_G_rectus	0.511240268	4	38	-24
Reproduction	Right_S_suborbital	0.511240268	5	39	-18
Reproduction	Right_G_temporal_middle	0.52401803	62	-23	-14
Reproduction	Right_G_cingul-Post-ventral	0.529037305	8	-48	7
Reproduction	Right-Inf-Lat-Vent	0.535400192	31	-14	-18
Reproduction	Left-Inf-Lat-Vent	0.557493396	-33	-20	-15
Reproduction	Right-Amygdala	0.578186635	23	-4	-20

Reproduction	Left_S_suborbital	0.578186635	-8	40	-14
Reproduction	Right_G_and_S_transv_frontopol	0.588269036	17	68	0
Reproduction	Left_S_temporal_inf	0.667487512	-55	-34	-16
Reproduction	Left_G_front_inf-Triangul	0.676747824	-52	31	4
Reproduction	Left_S_front_sup	0.678652762	-24	22	46
Reproduction	Left-vessel	0.680061387	-29	-3	-9
Reproduction	Left_G_temporal_inf	0.680061387	-55	-40	-23
Reproduction	Right_S_subparietal	0.695365913	9	-48	38
Reproduction	Optic-Chiasm	0.728164119	-1	1	-19
Reproduction	Right_G_cingul-Post-dorsal	0.728164119	3	-36	34
Reproduction	Left_S_orbital_lateral	0.733788516	-42	47	-5
Reproduction	Left-Amygdala	0.750667079	-24	-5	-20
Reproduction	WM-hypointensities	0.794609708	-4	-14	19
Reproduction	Left_G_front_middle	0.803681513	-38	34	34
Reproduction	Right_G_precuneus	0.803681513	6	-60	47
Reproduction	Left_G_orbital	0.806603521	-31	35	-19
Reproduction	Right_G_and_S_paracentral	0.807688467	7	-35	69
Reproduction	Left_G_cingul-Post-ventral	0.856542284	-8	-48	6
Reproduction	Left_G_temporal_middle	0.864465026	-63	-25	-13
Reproduction	Left_S_collat_transv_ant	0.877343295	-41	-20	-30
Reproduction	Left_S_temporal_sup	0.935035344	-51	-43	8
Reproduction	Right_G_pariet_inf-Angular	0.945288376	50	-58	42
Reproduction	Left_G_cingul-Post-dorsal	0.960459257	-3	-38	32
Reproduction	Right_S_central	0.983999911	37	-17	47
Scream	Left_G_insular_short	7.02E-09	-38	10	-4
Scream	Left_S_circular_insula_ant	7.02E-09	-31	22	-10
Scream	Left_S_circular_insula_inf	7.02E-09	-41	-12	-5
Scream	Left_S_temporal_transverse	7.02E-09	-53	-23	5
Scream	Right_G_front_inf-Triangul	7.02E-09	52	33	5
Scream	Right_G_temp_sup-Plan_tempo	7.02E-09	61	-28	20
Scream	Right_S_circular_insula_inf	7.02E-09	42	-8	-7
Scream	Right_G_temp_sup-Lateral	7.27E-09	59	-4	-7
Scream	Right_S_circular_insula_ant	1.34E-08	31	23	-10
Scream	Right_S_temporal_sup	1.34E-08	51	-38	10
Scream	Right_S_temporal_transverse	1.34E-08	53	-18	7
Scream	Right_G_insular_short	1.62E-08	38	12	-5
Scream	Left_G_temp_sup-Plan_tempo	1.96E-08	-59	-38	18
Scream	Left_G_temp_sup-Lateral	3.63E-08	-58	-7	-7
Scream	Right_G_front_inf-Opercular	9.92E-08	51	14	9
Scream	Right_Lat_Fis-ant-Horizont	9.92E-08	41	29	0
Scream	Right_G_temp_sup-Plan_polar	1.57E-07	36	9	-20
Scream	Right_G_temp_sup-G_T_transv	1.58E-07	48	-14	7
Scream	Left_Lat_Fis-ant-Horizont	2.34E-07	-44	32	-4
Scream	Left_G_temp_sup-G_T_transv	2.85E-07	-49	-18	8
Scream	Left_G_Ins_lg_and_S_cent_ins	3.62E-07	-38	-7	-2

Scream	Right_Lat_Fis-ant-Vertical	3.87E-07	48	23	10
Scream	Right-VentralDC	4.66E-07	10	-15	-10
Scream	Right_G_Ins_lg_and_S_cent_ins	5.90E-07	39	-4	-3
Scream	Left_Lat_Fis-ant-Vertical	5.99E-07	-48	21	11
Scream	Left_G_temp_sup-Plan_polar	1.09E-06	-41	8	-20
Scream	Right-Amygdala	1.98E-06	23	-4	-20
Scream	Left_G_front_inf-Opercular	6.79E-06	-51	12	10
Scream	Left-Amygdala	7.92E-06	-24	-5	-20
Scream	Right_G_front_inf-Orbital	1.42E-05	48	35	-11
Scream	Left_G_front_inf-Triangul	1.50E-05	-52	31	4
Scream	Right_G_temporal_middle	1.89E-05	62	-23	-14
Scream	Left_Lat_Fis-post	3.20E-05	-41	-34	20
Scream	Right_G_front_sup	3.20E-05	10	28	47
Scream	Left-Hippocampus	3.23E-05	-26	-23	-14
Scream	Left_G_and_S_cingul-Mid-Ant	3.23E-05	-7	16	35
Scream	Left-VentralDC	3.53E-05	-11	-16	-10
Scream	Left_S_temporal_sup	4.58E-05	-51	-43	8
Scream	Left_G_front_inf-Orbital	4.68E-05	-46	29	-10
Scream	Right_G_and_S_cingul-Ant	4.76E-05	7	41	8
Scream	Left_S_pericallosal	5.63E-05	-6	-4	17
Scream	Right_S_pericallosal	5.63E-05	6	-7	19
Scream	Left_G_and_S_cingul-Ant	6.09E-05	-8	41	6
Scream	Right-Inf-Lat-Vent	6.79E-05	31	-14	-18
Scream	Right_G_and_S_cingul-Mid-Ant	8.35E-05	7	17	35
Scream	Right_S_precentral-inf-part	8.87E-05	45	7	30
Scream	Right-Hippocampus	9.12E-05	26	-22	-14
Scream	Right_S_circular_insula_sup	1.02E-04	37	9	9
Scream	Left-Inf-Lat-Vent	2.62E-04	-33	-20	-15
Scream	Right_S_front_inf	2.67E-04	40	27	23
Scream	Left_G_and_S_cingul-Mid-Post	2.74E-04	-7	-11	41
Scream	Right_Lat_Fis-post	3.18E-04	42	-27	20
Scream	Left_S_circular_insula_sup	3.27E-04	-36	6	11
Scream	Left_G_pariet_inf-Supramar	3.58E-04	-60	-38	34
Scream	Right_G_and_S_cingul-Mid-Post	4.16E-04	7	-8	43
Scream	Right-Thalamus-Proper	5.75E-04	11	-17	7
Scream	Left_S_parieto_occipital	6.30E-04	-15	-68	25
Scream	Left_S_subparietal	9.47E-04	-10	-49	38
Scream	Right_S_parieto_occipital	0.001089738	18	-66	28
Scream	Left_G_subcallosal	0.001116183	-12	8	-13
Scream	Right_S_interm_prim-Jensen	0.001116183	52	-41	46
Scream	3rd-Ventricle	0.001127956	0	-11	-3
Scream	Left_S_interm_prim-Jensen	0.001223008	-52	-41	46
Scream	Left-Pallidum	0.001318209	-21	-5	-1
Scream	Left_G_temporal_middle	0.001495436	-63	-25	-13
Scream	Left-Thalamus-Proper	0.001729091	-11	-19	7

Scream	Right_G_pariet_inf-Supramar	0.001789212	59	-29	36
Scream	Right_G_subcallosal	0.002025375	5	12	-13
Scream	Right_S_subparietal	0.002051221	9	-48	38
Scream	Left_S_orbital_med-olfact	0.002709709	-13	25	-20
Scream	Left_S_cingul-Marginalis	0.003161972	-13	-38	49
Scream	Right_S_orbital_med-olfact	0.003195652	12	27	-19
Scream	Left_G_front_sup	0.003410165	-9	27	45
Scream	Left-Accumbens-area	0.00368321	-9	11	-8
Scream	CC_Central	0.004264653	0	-2	22
Scream	Right_S_cingul-Marginalis	0.004459525	12	-37	50
Scream	Right_S_orbital-H_Shaped	0.005700385	25	40	-16
Scream	Right_G_and_S_occipital_inf	0.006018261	43	-76	-12
Scream	Right_G_orbital	0.006316265	29	38	-19
Scream	Left_G_oc-temp_med-Parahip	0.007035158	-23	-16	-26
Scream	Left_G_orbital	0.00839027	-31	35	-19
Scream	Optic-Chiasm	0.0086827	-1	1	-19
Scream	Right_G_postcentral	0.009877765	47	-21	54
Scream	Right_S_oc_middle_and_Lunatus	0.009932567	37	-79	10
Scream	Left_S_front_inf	0.01090931	-41	27	20
Scream	Right_G_cingul-Post-dorsal	0.011450581	3	-36	34
Scream	Brain-Stem	0.011908257	0	-31	-34
Scream	Left_S_collat_transv_ant	0.012180998	-41	-20	-30
Scream	Right_G_cingul-Post-ventral	0.013240084	8	-48	7
Scream	Right-Pallidum	0.013975103	21	-4	-2
Scream	Left_G_cingul-Post-ventral	0.015578334	-8	-48	6
Scream	Right_G_pariet_inf-Angular	0.0157451	50	-58	42
Scream	Right_S_orbital_lateral	0.016550901	42	44	-3
Scream	Left-vessel	0.017460363	-29	-3	-9
Scream	Left_S_orbital-H_Shaped	0.01760849	-26	40	-16
Scream	Right_G_and_S_subcentral	0.017816862	57	-8	14
Scream	Right_S_postcentral	0.01830102	38	-30	49
Scream	Right_G_and_S_transv_frontopol	0.019202218	17	68	0
Scream	CSF	0.020746157	0	-19	10
Scream	Left_G_cingul-Post-dorsal	0.021655667	-3	-38	32
Scream	Left_S_precentral-inf-part	0.023722747	-45	4	30
Scream	Right-Accumbens-area	0.024041731	8	12	-8
Scream	Right_S_oc_sup_and_transversal	0.028141418	30	-79	23
Scream	Right-Caudate	0.0288401	13	11	9
Scream	Left_G_and_S_transv_frontopol	0.02892395	-17	67	-3
Scream	Left-Caudate	0.035198409	-14	10	10
Scream	Left_G_precuneus	0.035198409	-5	-60	45
Scream	Left_S_calcarine	0.038265826	-15	-69	5
Scream	Left-Putamen	0.040492144	-26	1	0
Scream	Left_S_suborbital	0.040492144	-8	40	-14
Scream	Right-vessel	0.04061639	29	-2	-10

Scream	Right_G_and_S_frontomargin	0.041731142	23	62	-11
Scream	Right_G_precuneus	0.043778259	6	-60	47
Scream	Left-Cerebellum-White-Matter	0.049484419	-20	-57	-33
Scream	Left-Cerebral-White-Matter	0.052972738	-29	-19	19
Scream	Right-Cerebral-White-Matter	0.056247421	29	-18	19
Scream	Right-Putamen	0.056247421	26	2	0
Scream	Left_G_and_S_subcentral	0.056247421	-57	-11	15
Scream	Right_S_occipital_ant	0.056247421	46	-64	5
Scream	Right_S_front_middle	0.062016665	30	47	19
Scream	Right_Pole_temporal	0.063570513	32	9	-42
Scream	Left_S_occipital_ant	0.066879515	-41	-70	7
Scream	Left_S_oc-temp_med_and_Lingual	0.074692971	-28	-48	-11
Scream	Left_G_cuneus	0.076407659	-4	-84	19
Scream	CC_Mid_Posterior	0.07726033	0	-18	22
Scream	Left-Cerebellum-Cortex	0.079195542	-25	-63	-37
Scream	Left_G_postcentral	0.079195542	-47	-25	55
Scream	Right_S_calcarine	0.080790875	18	-65	7
Scream	Left_S_oc_middle_and_Lunatus	0.082184167	-33	-87	8
Scream	Left_Pole_temporal	0.083202883	-33	6	-42
Scream	Right_S_collat_transv_post	0.084805418	26	-79	-8
Scream	Left_G_and_S_occipital_inf	0.089054117	-39	-83	-10
Scream	Right_G_oc-temp_med-Parahip	0.090411571	24	-13	-29
Scream	Left_S_front_middle	0.091925218	-29	46	19
Scream	Right_G_front_middle	0.091925218	38	36	33
Scream	Right_S_front_sup	0.091925218	23	25	45
Scream	Left_G_pariet_inf-Angular	0.092778968	-44	-69	41
Scream	CC_Anterior	0.09512064	0	25	5
Scream	Left_S_postcentral	0.102151263	-42	-33	46
Scream	Right_G_temporal_inf	0.109168744	55	-35	-25
Scream	Left_G_front_middle	0.109584148	-38	34	34
Scream	Right_G_rectus	0.109584148	4	38	-24
Scream	Right_S_suborbital	0.110988059	5	39	-18
Scream	Left_G_and_S_frontomargin	0.119076232	-24	61	-10
Scream	Right_G_occipital_middle	0.119919736	45	-79	15
Scream	Right_S_temporal_inf	0.120829713	55	-33	-16
Scream	Left_G_rectus	0.123513503	-5	36	-23
Scream	CC_Mid_Anterior	0.123762463	0	13	19
Scream	Left_G_temporal_inf	0.167944091	-55	-40	-23
Scream	Right_G_cuneus	0.168327649	7	-80	21
Scream	Right-Cerebellum-White-Matter	0.17080153	19	-56	-33
Scream	Left_S_front_sup	0.17080153	-24	22	46
Scream	Left_S_oc_sup_and_transversal	0.17080153	-27	-84	21
Scream	Right_S_collat_transv_ant	0.17080153	41	-18	-29
Scream	4th-Ventricle	0.18472416	-1	-46	-35
Scream	CC_Posterior	0.18472416	0	-35	17

Scream	Left_S_oc-temp_lat	0.18472416	-44	-50	-14
Scream	Left_G_oc-temp_med-Lingual	0.222450678	-10	-73	-6
Scream	Right_G_oc-temp_med-Lingual	0.247226939	10	-67	-4
Scream	Left_S_orbital_lateral	0.248403349	-42	47	-5
Scream	Right-Cerebellum-Cortex	0.249465658	24	-63	-37
Scream	Right_Pole_occipital	0.276408687	20	-95	-3
Scream	Right-choroid-plexus	0.333233897	20	-24	8
Scream	Left_Pole_occipital	0.334825563	-16	-101	-4
Scream	Right_G_oc-temp_lat-fusifor	0.334825563	35	-48	-21
Scream	Right_S_intrapariet_and_P_trans	0.339088165	30	-56	47
Scream	Right_S_central	0.352537708	37	-17	47
Scream	WM-hypointensities	0.377858988	-4	-14	19
Scream	Left_S_central	0.417689775	-39	-19	46
Scream	Left_G_occipital_middle	0.436302307	-39	-88	12
Scream	Right_G_parietal_sup	0.458007834	25	-60	62
Scream	Right_S_oc-temp_lat	0.464672708	45	-47	-15
Scream	Right_S_oc-temp_med_and_Lingual	0.472761316	28	-48	-11
Scream	Left_S_collat_transv_post	0.591567293	-24	-81	-7
Scream	Right_G_precentral	0.632139544	42	-7	53
Scream	Right_G_occipital_sup	0.700061826	20	-89	31
Scream	Right_S_precentral-sup-part	0.702173418	29	-9	56
Scream	Left-choroid-plexus	0.703861849	-22	-31	7
Scream	Left_S_temporal_inf	0.717431314	-55	-34	-16
Scream	Left_S_intrapariet_and_P_trans	0.728183481	-32	-58	43
Scream	Left_G_precentral	0.80278854	-43	-9	53
Scream	Left-Lateral-Ventricle	0.823484122	-15	-17	14
Scream	Left_G_parietal_sup	0.852957671	-26	-61	59
Scream	Right_G_and_S_paracentral	0.852957671	7	-35	69
Scream	Right-Lateral-Ventricle	0.856868765	14	-14	14
Scream	Left_S_precentral-sup-part	0.903864628	-29	-10	56
Scream	Left_G_oc-temp_lat-fusifor	0.933060952	-36	-55	-19
Scream	Left_G_and_S_paracentral	0.93778565	-8	-36	68
Scream	Left_G_occipital_sup	0.99333929	-15	-92	29

Table S6 – ROI-based repeated-measures ANOVA results comparing Encoding vs. Reproduction in Safe condition.

The table reports regions showing Regressor effects (Encoding vs. Reproduction), listing the direction of the contrast, ROI label, degrees of freedom (df), F-values, uncorrected p-values, and FDR-corrected p-values, together with MNI coordinates (X, Y, Z). ROIs are labeled according to the Destrieux atlas nomenclature.

**Contrast**	**ROI**	**df**	**F-value**	**p-value**	**p-value (FDR-corr)**	**X**	**Y**	**Z**
Encoding < Reproduction	Left_S_precentral-sup-part	29	103.756	4.34E-11	8.38E-09	-29	-10	56
Encoding < Reproduction	Right_G_and_S_cingul-Mid-Ant	29	96.893	9.43E-11	9.10E-09	7	17	35
Encoding < Reproduction	Left_G_precentral	29	83.417	4.95E-10	3.18E-08	-43	-9	53
Encoding < Reproduction	Right_S_circular_insula_sup	29	77.423	1.11E-09	5.36E-08	37	9	9
Encoding < Reproduction	Right_G_front_inf-Opercular	29	73.644	1.88E-09	7.26E-08	51	14	9
Encoding < Reproduction	Left_S_postcentral	29	72.15	2.33E-09	7.49E-08	-42	-33	46
Encoding < Reproduction	Left_G_and_S_cingul-Mid-Ant	29	65.115	6.72E-09	1.65E-07	-7	16	35
Encoding < Reproduction	Right_Lat_Fis-ant-Horizont	29	65.026	6.82E-09	1.65E-07	41	29	0
Encoding < Reproduction	Left_G_postcentral	29	63.188	9.12E-09	1.96E-07	-47	-25	55
Encoding < Reproduction	Right_S_precentral-inf-part	29	59.819	1.58E-08	3.05E-07	45	7	30
Encoding < Reproduction	Left_S_central	29	57.36	2.38E-08	4.18E-07	-39	-19	46
Encoding < Reproduction	Left_S_circular_insula_sup	29	53.3	4.85E-08	7.80E-07	-36	6	11
Encoding < Reproduction	Left-Pallidum	29	52.013	6.12E-08	9.09E-07	-21	-5	-1
Encoding < Reproduction	Right_S_precentral-sup-part	29	48.763	1.12E-07	1.54E-06	29	-9	56
Encoding < Reproduction	Left-Putamen	29	48.156	1.26E-07	1.62E-06	-26	1	0
Encoding < Reproduction	Left_G_front_inf-Opercular	29	47.239	1.50E-07	1.64E-06	-51	12	10
Encoding < Reproduction	Left_G_pariet_inf-Supramar	29	47.134	1.53E-07	1.64E-06	-60	-38	34
Encoding < Reproduction	Left_S_intrapariet_and_P_trans	29	47.482	1.43E-07	1.64E-06	-32	-58	43
Encoding < Reproduction	Right_Lat_Fis-ant-Vertical	29	46.27	1.81E-07	1.84E-06	48	23	10
Encoding < Reproduction	Right_G_insular_short	29	43.561	3.12E-07	3.01E-06	38	12	-5
Encoding < Reproduction	Right_G_pariet_inf-Supramar	29	40.698	5.65E-07	5.19E-06	59	-29	36
Encoding < Reproduction	Right-Caudate	29	40.087	6.44E-07	5.65E-06	13	11	9
Encoding < Reproduction	Right_S_circular_insula_ant	29	38.483	9.13E-07	7.34E-06	31	23	-10

Encoding < Reproduction	Right_S_intrapariet_and_P_trans	29	38.658	8.78E-07	7.34E-06	30	-56	47
Encoding < Reproduction	Right_G_front_middle	29	38.277	9.55E-07	7.37E-06	38	36	33
Encoding < Reproduction	Right_G_precentral	29	37.283	1.19E-06	8.58E-06	42	-7	53
Encoding < Reproduction	Right_S_interm_prim-Jensen	29	37.258	1.20E-06	8.58E-06	52	-41	46
Encoding < Reproduction	Right-Cerebellum-Cortex	29	36.624	1.38E-06	9.51E-06	24	-63	-37
Encoding < Reproduction	Left-Thalamus-Proper	29	36.372	1.46E-06	9.72E-06	-11	-19	7
Encoding < Reproduction	Left_G_insular_short	29	34.871	2.07E-06	1.33E-05	-38	10	-4
Encoding < Reproduction	Left_S_circular_insula_ant	29	33.849	2.63E-06	1.64E-05	-31	22	-10
Encoding < Reproduction	Left_S_precentral-inf-part	29	33.315	2.98E-06	1.80E-05	-45	4	30
Encoding < Reproduction	Right-Pallidum	29	32.626	3.52E-06	2.06E-05	21	-4	-2
Encoding < Reproduction	Right-Putamen	29	32.452	3.67E-06	2.08E-05	26	2	0
Encoding < Reproduction	Right_G_front_sup	29	32.156	3.95E-06	2.18E-05	10	28	47
Encoding < Reproduction	Right_S_orbital_med-olfact	29	31.798	4.31E-06	2.31E-05	12	27	-19
Encoding < Reproduction	Right-Cerebellum-White-Matter	29	31.597	4.52E-06	2.36E-05	19	-56	-33
Encoding < Reproduction	Right-Thalamus-Proper	29	30.863	5.43E-06	2.76E-05	11	-17	7
Encoding < Reproduction	Left-Caudate	29	30.407	6.08E-06	2.86E-05	-14	10	10
Encoding < Reproduction	Left_G_front_sup	29	30.537	5.89E-06	2.86E-05	-9	27	45
Encoding < Reproduction	Left_G_temp_sup-Plan_tempo	29	30.449	6.02E-06	2.86E-05	-59	-38	18
Encoding < Reproduction	Left_G_and_S_subcentral	29	29.43	7.79E-06	3.58E-05	-57	-11	15
Encoding < Reproduction	Left_G_parietal_sup	29	29.343	7.97E-06	3.58E-05	-26	-61	59
Encoding < Reproduction	Right_S_front_middle	29	29.203	8.26E-06	3.62E-05	30	47	19
Encoding < Reproduction	Right-Accumbens-area	29	29.048	8.59E-06	3.68E-05	8	12	-8
Encoding < Reproduction	Left_G_and_S_cingul-Mid-Post	29	28.191	1.07E-05	4.49E-05	-7	-11	41
Encoding < Reproduction	Left-Cerebellum-Cortex	29	28.074	1.11E-05	4.56E-05	-25	-63	-37
Encoding < Reproduction	Right-VentralDC	29	26.627	1.63E-05	6.55E-05	10	-15	-10
Encoding < Reproduction	Left_S_front_inf	29	26.138	1.86E-05	7.33E-05	-41	27	20
Encoding < Reproduction	Right_S_orbital_lateral	29	25.5	2.21E-05	8.53E-05	42	44	-3
Encoding < Reproduction	Right_G_front_inf-Triangul	29	24.88	2.62E-05	9.91E-05	52	33	5
Encoding < Reproduction	3rd-Ventricle	29	24.073	3.29E-05	1.22E-04	0	-11	-3

Encoding < Reproduction	Right_S_front_inf	29	23.563	3.80E-05	1.38E-04	40	27	23
Encoding < Reproduction	Right-Cerebral-White-Matter	29	23.28	4.13E-05	1.48E-04	29	-18	19
Encoding < Reproduction	Left_Lat_Fis-ant-Vertical	29	22.684	4.90E-05	1.72E-04	-48	21	11
Encoding < Reproduction	Left_S_temporal_transverse	29	22.303	5.48E-05	1.89E-04	-53	-23	5
Encoding < Reproduction	Left-VentralDC	29	22.01	5.98E-05	2.02E-04	-11	-16	-10
Encoding < Reproduction	Right_G_front_inf-Orbital	29	20.898	8.33E-05	2.77E-04	48	35	-11
Encoding < Reproduction	Right_S_postcentral	29	20.837	8.49E-05	2.78E-04	38	-30	49
Encoding < Reproduction	Right-Lateral-Ventricle	29	20.494	9.42E-05	3.03E-04	14	-14	14
Encoding < Reproduction	Right_S_oc_sup_and_transversal	29	20.376	9.77E-05	3.09E-04	30	-79	23
Encoding < Reproduction	Left-Cerebral-White-Matter	29	20.279	1.01E-04	3.14E-04	-29	-19	19
Encoding < Reproduction	Left_Lat_Fis-post	29	19.818	1.16E-04	3.55E-04	-41	-34	20
Encoding < Reproduction	Right_G_temp_sup-Plan_tempo	29	19.635	1.23E-04	3.71E-04	61	-28	20
Encoding < Reproduction	Left-Cerebellum-White-Matter	29	19.159	1.43E-04	4.24E-04	-20	-57	-33
Encoding < Reproduction	Right_G_temp_sup-G_T_transv	29	19.113	1.45E-04	4.24E-04	48	-14	7
Encoding < Reproduction	Left_S_front_middle	29	18.831	1.58E-04	4.48E-04	-29	46	19
Encoding < Reproduction	Right_S_temporal_transverse	29	18.824	1.58E-04	4.48E-04	53	-18	7
Encoding < Reproduction	Left_G_temp_sup-G_T_transv	29	18.387	1.82E-04	5.09E-04	-49	-18	8
Encoding < Reproduction	Right_G_oc-temp_lat-fusifor	29	18.136	1.97E-04	5.43E-04	35	-48	-21
Encoding < Reproduction	CSF	29	18.059	2.02E-04	5.49E-04	0	-19	10
Encoding < Reproduction	Right_G_temp_sup-Lateral	29	17.99	2.07E-04	5.55E-04	59	-4	-7
Encoding < Reproduction	Left-vessel	29	17.838	2.17E-04	5.74E-04	-29	-3	-9
Encoding < Reproduction	Right_G_and_S_subcentral	29	17.525	2.41E-04	6.29E-04	57	-8	14
Encoding < Reproduction	Right_G_and_S_cingul-Ant	29	16.903	2.96E-04	7.62E-04	7	41	8
Encoding < Reproduction	Right_G_and_S_cingul-Mid-Post	29	15.657	4.50E-04	0.001142763	7	-8	43
Encoding < Reproduction	Left_S_oc_sup_and_transversal	29	15.525	4.70E-04	0.001178052	-27	-84	21
Encoding < Reproduction	Right_G_orbital	29	14.717	6.23E-04	0.001541526	29	38	-19
Encoding < Reproduction	Right_G_and_S_occipital_inf	29	14.222	7.42E-04	0.001812734	43	-76	-12
Encoding < Reproduction	Left_G_front_middle	29	14.048	7.89E-04	0.001903463	-38	34	34
Encoding < Reproduction	Right-choroid-plexus	29	14.012	7.99E-04	0.00190379	20	-24	8

Encoding < Reproduction	Brain-Stem	29	13.844	8.49E-04	0.001998256	0	-31	-34
Encoding < Reproduction	Left-Accumbens-area	29	12.606	0.001	0.002144444	-9	11	-8
Encoding < Reproduction	CC_Mid_Posterior	29	12.726	0.001	0.002144444	0	-18	22
Encoding < Reproduction	Left_G_oc-temp_med-Lingual	29	13.197	0.001	0.002144444	-10	-73	-6
Encoding < Reproduction	Left_Lat_Fis-ant-Horizont	29	12.864	0.001	0.002144444	-44	32	-4
Encoding < Reproduction	Right_G_and_S_frontomargin	29	12.653	0.001	0.002144444	23	62	-11
Encoding < Reproduction	Right_G_oc-temp_med-Lingual	29	12.408	0.001	0.002144444	10	-67	-4
Encoding < Reproduction	Right_G_temp_sup-Plan_polar	29	12.36	0.001	0.002144444	36	9	-20
Encoding < Reproduction	Right_S_circular_insula_inf	29	13.505	9.60E-04	0.002144444	42	-8	-7
Encoding < Reproduction	WM-hypointensities	29	11.695	0.002	0.004063158	-4	-14	19
Encoding < Reproduction	CC_Central	29	11.201	0.002	0.004063158	0	-2	22
Encoding < Reproduction	CC_Anterior	29	11.793	0.002	0.004063158	0	25	5
Encoding < Reproduction	Left_G_Ins_lg_and_S_cent_ins	29	12.12	0.002	0.004063158	-38	-7	-2
Encoding < Reproduction	Right_G_Ins_lg_and_S_cent_ins	29	11.895	0.002	0.004063158	39	-4	-3
Encoding < Reproduction	Left-Lateral-Ventricle	29	10.888	0.003	0.005732673	-15	-17	14
Encoding < Reproduction	Left_S_collat_transv_post	29	10.625	0.003	0.005732673	-24	-81	-7
Encoding < Reproduction	Right_S_collat_transv_post	29	10.386	0.003	0.005732673	26	-79	-8
Encoding < Reproduction	Right_S_oc-temp_lat	29	10.549	0.003	0.005732673	45	-47	-15
Encoding < Reproduction	Right_S_pericallosal	29	10.504	0.003	0.005732673	6	-7	19
Encoding < Reproduction	Right_S_temporal_sup	29	10.226	0.003	0.005732673	51	-38	10
Encoding < Reproduction	CC_Posterior	29	9.684	0.004	0.007423077	0	-35	17
Encoding < Reproduction	Right_G_pariet_inf-Angular	29	9.659	0.004	0.007423077	50	-58	42
Encoding < Reproduction	Right_Lat_Fis-post	29	9.582	0.004	0.007423077	42	-27	20
Encoding < Reproduction	Left_G_and_S_frontomargin	29	9.482	0.005	0.008693694	-24	61	-10
Encoding < Reproduction	Left_G_and_S_occipital_inf	29	9.111	0.005	0.008693694	-39	-83	-10
Encoding < Reproduction	Left_G_oc-temp_lat-fusifor	29	9.182	0.005	0.008693694	-36	-55	-19
Encoding < Reproduction	Left_S_circular_insula_inf	29	9.147	0.005	0.008693694	-41	-12	-5
Encoding < Reproduction	Left_S_occipital_ant	29	9.444	0.005	0.008693694	-41	-70	7
Encoding < Reproduction	Right_G_parietal_sup	29	9.252	0.005	0.008693694	25	-60	62

Encoding < Reproduction	Right_S_orbital-H_Shaped	29	9.12	0.005	0.008693694	25	40	-16
Encoding < Reproduction	Left_G_subcallosal	29	8.795	0.006	0.010157895	-12	8	-13
Encoding < Reproduction	Right_G_subcallosal	29	8.898	0.006	0.010157895	5	12	-13
Encoding < Reproduction	Right_S_oc-temp_med_and_Lingual	29	8.737	0.006	0.010157895	28	-48	-11
Encoding < Reproduction	Left_G_front_inf-Triangul	29	8.093	0.008	0.013426087	-52	31	4
Encoding < Reproduction	Left_G_temp_sup-Lateral	29	7.776	0.009	0.014846154	-58	-7	-7
Encoding < Reproduction	Right_G_temporal_inf	29	7.917	0.009	0.014846154	55	-35	-25
Encoding < Reproduction	Left_S_calcarine	29	7.637	0.01	0.016218487	-15	-69	5
Encoding < Reproduction	Left_S_pericallosal	29	7.596	0.01	0.016218487	-6	-4	17
Encoding < Reproduction	Left_G_cuneus	29	7.377	0.011	0.017691667	-4	-84	19
Encoding < Reproduction	Right_S_calcarine	29	7.023	0.013	0.020565574	18	-65	7
Encoding < Reproduction	Right_S_occipital_ant	29	7.085	0.013	0.020565574	46	-64	5
Encoding < Reproduction	Right_G_temporal_middle	29	6.903	0.014	0.02196748	62	-23	-14
Encoding < Reproduction	Right_G_occipital_middle	29	6.676	0.015	0.023346774	45	-79	15
Encoding < Reproduction	Left_S_orbital-H_Shaped	29	6.374	0.017	0.026248	-26	40	-16
Encoding < Reproduction	Left_S_cingul-Marginalis	29	6.251	0.018	0.027354331	-13	-38	49
Encoding < Reproduction	Left_S_oc-temp_lat	29	6.269	0.018	0.027354331	-44	-50	-14
Encoding < Reproduction	Left_S_oc-temp_med_and_Lingual	29	6.229	0.019	0.028426357	-28	-48	-11
Encoding < Reproduction	Right_G_occipital_sup	29	6.215	0.019	0.028426357	20	-89	31
Encoding < Reproduction	Left_S_orbital_med-olfact	29	6.067	0.02	0.029692308	-13	25	-20
Encoding < Reproduction	Right_G_cuneus	29	5.88	0.022	0.032412214	7	-80	21
Encoding < Reproduction	Left_G_orbital	29	5.698	0.024	0.035090909	-31	35	-19
Encoding < Reproduction	Right_S_suborbital	29	5.499	0.026	0.037729323	5	39	-18
Encoding < Reproduction	Left_S_orbital_lateral	29	5.409	0.027	0.03888806	-42	47	-5
Encoding < Reproduction	Left_G_occipital_sup	29	5.289	0.029	0.041154412	-15	-92	29
Encoding < Reproduction	Right_S_temporal_inf	29	5.267	0.029	0.041154412	55	-33	-16
Encoding < Reproduction	Left_G_front_inf-Orbital	29	5.213	0.03	0.042262774	-46	29	-10
Encoding < Reproduction	Right_S_front_sup	29	5.168	0.031	0.043355072	23	25	45
Encoding < Reproduction	Right_S_parieto_occipital	29	5.006	0.033	0.045820144	18	-66	28

Encoding < Reproduction	Left_G_and_S_cingul-Ant	29	4.778	0.037	0.051007143	-8	41	6
Encoding < Reproduction	CC_Mid_Anterior	29	4.569	0.041	0.055725352	0	13	19
Encoding < Reproduction	Right_S_oc_middle_and_Lunatus	29	4.541	0.042	0.056685315	37	-79	10
Encoding < Reproduction	Left_G_occipital_middle	29	4.48	0.043	0.057631944	-39	-88	12
Encoding < Reproduction	Right_G_postcentral	29	4.389	0.045	0.059896552	47	-21	54
Encoding < Reproduction	Right_G_and_S_transv_frontopol	29	3.944	0.057	0.075349315	17	68	0
Encoding < Reproduction	Right_G_oc-temp_med-Parahip	29	3.73	0.063	0.082714286	24	-13	-29
Encoding < Reproduction	Right_S_cingul-Marginalis	29	3.242	0.082	0.106932432	12	-37	50
Encoding < Reproduction	Right_G_rectus	29	3.067	0.09	0.116577181	4	38	-24
Encoding < Reproduction	Left-choroid-plexus	29	2.809	0.105	0.134205298	-22	-31	7
Encoding < Reproduction	4th-Ventricle	29	2.789	0.106	0.134592105	-1	-46	-35
Encoding < Reproduction	Left_G_rectus	29	2.598	0.118	0.147883117	-5	36	-23
Encoding < Reproduction	Left_G_temp_sup-Plan_polar	29	2.591	0.118	0.147883117	-41	8	-20
Encoding < Reproduction	Left_S_oc_middle_and_Lunatus	29	2.548	0.121	0.150664516	-33	-87	8
Encoding < Reproduction	Left_G_temporal_inf	29	2.473	0.127	0.157121795	-55	-40	-23
Encoding < Reproduction	Right_S_collat_transv_ant	29	2.258	0.144	0.177019108	41	-18	-29
Encoding < Reproduction	Right-Hippocampus	29	1.718	0.2	0.242767296	26	-22	-14
Encoding < Reproduction	Right-Inf-Lat-Vent	29	1.59	0.217	0.26175625	31	-14	-18
Encoding < Reproduction	Right_G_cingul-Post-dorsal	29	1.554	0.222	0.266124224	3	-36	34
Encoding < Reproduction	Left_G_oc-temp_med-Parahip	29	1.502	0.23	0.272331288	-23	-16	-26
Encoding < Reproduction	Left_S_collat_transv_ant	29	1.502	0.23	0.272331288	-41	-20	-30
Encoding < Reproduction	Left_S_parieto_occipital	29	1.369	0.252	0.294763636	-15	-68	25
Encoding < Reproduction	Left_G_and_S_paracentral	29	1.099	0.303	0.350173653	-8	-36	68
Encoding < Reproduction	Right_S_subparietal	29	1.098	0.303	0.350173653	9	-48	38
Encoding < Reproduction	Left_S_front_sup	29	1.024	0.32	0.367619048	-24	22	46
Encoding < Reproduction	Left_S_suborbital	29	0.981	0.33	0.375782353	-8	40	-14
Encoding < Reproduction	Right_S_central	29	0.977	0.331	0.375782353	37	-17	47
Encoding < Reproduction	Right_Pole_occipital	29	0.871	0.358	0.40405848	20	-95	-3
Encoding < Reproduction	Right-Amygdala	29	0.811	0.375	0.420784884	23	-4	-20

Encoding < Reproduction	Left_S_temporal_inf	29	0.778	0.385	0.429508671	-55	-34	-16
Encoding < Reproduction	Left_G_temporal_middle	29	0.71	0.406	0.44776	-63	-25	-13
Encoding < Reproduction	Right_G_cingul-Post-ventral	29	0.537	0.469	0.514301136	8	-48	7
Encoding < Reproduction	Left_S_temporal_sup	29	0.524	0.475	0.517937853	-51	-43	8
Encoding < Reproduction	Right_G_and_S_paracentral	29	0.311	0.581	0.629960674	7	-35	69
Encoding < Reproduction	Left-Hippocampus	29	0.259	0.615	0.663100559	-26	-23	-14
Encoding < Reproduction	Left_Pole_temporal	29	0.245	0.624	0.669066667	-33	6	-42
Encoding < Reproduction	Right_G_precuneus	29	0.2	0.658	0.701624309	6	-60	47
Encoding < Reproduction	Left_G_cingul-Post-dorsal	29	0.182	0.673	0.713675824	-3	-38	32
Encoding < Reproduction	Optic-Chiasm	29	0.176	0.678	0.71504918	-1	1	-19
Encoding < Reproduction	Left_G_cingul-Post-ventral	29	0.091	0.765	0.798081081	-8	-48	6
Encoding < Reproduction	Right_Pole_temporal	29	0.061	0.807	0.832893048	32	9	-42
Encoding < Reproduction	Left_G_pariet_inf-Angular	29	0.056	0.814	0.835648936	-44	-69	41
Encoding < Reproduction	Left-Amygdala	29	0.03	0.865	0.882721053	-24	-5	-20
Encoding < Reproduction	Left_Pole_occipital	29	0.003	0.96	0.965	-16	-101	-4
Encoding > Reproduction	Left_G_and_S_transv_frontopol	29	0.139	0.712	0.746826087	-17	67	-3
Encoding > Reproduction	Left-Inf-Lat-Vent	29	0.064	0.802	0.832182796	-33	-20	-15
Encoding > Reproduction	Left_G_precuneus	29	0.028	0.869	0.882721053	-5	-60	45
Encoding > Reproduction	Left_S_subparietal	29	0.02	0.889	0.898308901	-10	-49	38
Encoding > Reproduction	Left_S_interm_prim-Jensen	29	4.81E-04	0.983	0.983	-52	-41	46

Table S7 – ROI-based repeated-measures ANOVA results comparing Encoding vs. Reproduction in Threat condition.

The table reports regions showing Regressor effects (Encoding vs. Reproduction), listing the direction of the contrast, ROI label, degrees of freedom (df), F-values, uncorrected p-values, and FDR-corrected p-values, together with MNI coordinates (X, Y, Z). ROIs are labeled according to the Destrieux atlas nomenclature.

Contrast	ROI	df	F-value	p-value	p-value (FDR-corr)	X	Y	Z
Encoding < Reproduction	Left_G_postcentral	29	63.886	8.16E-09	1.57E-06	-47	-25	55
Encoding < Reproduction	Left_S_central	29	34.597	2.20E-06	1.98E-04	-39	-19	46
Encoding < Reproduction	Left_S_postcentral	29	33.18	3.08E-06	1.98E-04	-42	-33	46
Encoding < Reproduction	Right_S_intrapariet_and_P_trans	29	29.639	7.39E-06	3.57E-04	30	-56	47
Encoding < Reproduction	Left_G_precentral	29	22.89	4.62E-05	0.00178332	-43	-9	53
Encoding < Reproduction	Right_S_interm_prim-Jensen	29	20.852	8.45E-05	0.002718083	52	-41	46
Encoding < Reproduction	Right_S_precentral-inf-part	29	19.791	1.17E-04	0.003225857	45	7	30
Encoding < Reproduction	Left_S_intrapariet_and_P_trans	29	18.025	2.05E-04	0.004945625	-32	-58	43
Encoding < Reproduction	Right_G_oc-temp_lat-fusifor	29	16.71	3.15E-04	0.006755	35	-48	-21
Encoding < Reproduction	Right_G_precentral	29	16.28	3.64E-04	0.0070252	42	-7	53
Encoding < Reproduction	Right-Lateral-Ventricle	29	15.031	5.58E-04	0.009790364	14	-14	14
Encoding < Reproduction	Right_G_front_inf-Opercular	29	14.624	6.44E-04	0.010157895	51	14	9
Encoding < Reproduction	Right_G_and_S_cingul-Mid-Ant	29	13.983	8.08E-04	0.010157895	7	17	35
Encoding < Reproduction	Right_G_front_middle	29	13.698	8.95E-04	0.010157895	38	36	33
Encoding < Reproduction	Left_G_and_S_occipital_inf	29	12.476	0.001	0.010157895	-39	-83	-10
Encoding < Reproduction	Left_G_and_S_cingul-Mid-Ant	29	12.601	0.001	0.010157895	-7	16	35
Encoding < Reproduction	Right_G_and_S_occipital_inf	29	13.107	0.001	0.010157895	43	-76	-12
Encoding < Reproduction	Right_S_circular_insula_sup	29	13.165	0.001	0.010157895	37	9	9
Encoding < Reproduction	Right_S_postcentral	29	12.472	0.001	0.010157895	38	-30	49
Encoding < Reproduction	Left_S_circular_insula_sup	29	11.236	0.002	0.017545455	-36	6	11
Encoding < Reproduction	Left_S_occipital_ant	29	11.604	0.002	0.017545455	-41	-70	7
Encoding < Reproduction	Right_S_front_middle	29	12.167	0.002	0.017545455	30	47	19
Encoding < Reproduction	Right_G_insular_short	29	10.955	0.003	0.02316	38	12	-5

Encoding < Reproduction	Right_G_pariet_inf-Supramar	29	10.147	0.003	0.02316	59	-29	36
Encoding < Reproduction	Right_S_oc-temp_lat	29	10.641	0.003	0.02316	45	-47	-15
Encoding < Reproduction	Right_S_precentral-sup-part	29	9.795	0.004	0.029692308	29	-9	56
Encoding < Reproduction	Left-Putamen	29	9.478	0.005	0.03015625	-26	1	0
Encoding < Reproduction	CC_Mid_Posterior	29	9.004	0.005	0.03015625	0	-18	22
Encoding < Reproduction	CC_Central	29	9.473	0.005	0.03015625	0	-2	22
Encoding < Reproduction	Left_Lat_Fis-ant-Vertical	29	9.285	0.005	0.03015625	-48	21	11
Encoding < Reproduction	Right_G_oc-temp_med-Lingual	29	9.158	0.005	0.03015625	10	-67	-4
Encoding < Reproduction	Right_S_oc-temp_med_and_Lingual	29	9.241	0.005	0.03015625	28	-48	-11
Encoding < Reproduction	Left_G_front_inf-Opercular	29	8.946	0.006	0.032166667	-51	12	10
Encoding < Reproduction	Left_G_oc-temp_lat-fusifor	29	8.925	0.006	0.032166667	-36	-55	-19
Encoding < Reproduction	Right_S_oc_sup_and_transversal	29	8.922	0.006	0.032166667	30	-79	23
Encoding < Reproduction	Right_S_temporal_transverse	29	8.682	0.006	0.032166667	53	-18	7
Encoding < Reproduction	Left_G_and_S_subcentral	29	8.273	0.007	0.036513514	-57	-11	15
Encoding < Reproduction	Left_G_parietal_sup	29	8.084	0.008	0.039589744	-26	-61	59
Encoding < Reproduction	Left_G_temp_sup-G_T_transv	29	8.193	0.008	0.039589744	-49	-18	8
Encoding < Reproduction	Left_S_precentral-sup-part	29	7.851	0.009	0.042365854	-29	-10	56
Encoding < Reproduction	Right_S_front_inf	29	7.882	0.009	0.042365854	40	27	23
Encoding < Reproduction	Right-Pallidum	29	7.54	0.01	0.042888889	21	-4	-2
Encoding < Reproduction	Left_G_pariet_inf-Supramar	29	7.712	0.01	0.042888889	-60	-38	34
Encoding < Reproduction	Right_G_front_sup	29	7.546	0.01	0.042888889	10	28	47
Encoding < Reproduction	Right_Lat_Fis-ant-Horizont	29	7.53	0.01	0.042888889	41	29	0
Encoding < Reproduction	3rd-Ventricle	29	7.398	0.011	0.044229167	0	-11	-3
Encoding < Reproduction	Left_S_precentral-inf-part	29	7.333	0.011	0.044229167	-45	4	30
Encoding < Reproduction	Right_S_orbital-H_Shaped	29	7.469	0.011	0.044229167	25	40	-16
Encoding < Reproduction	Right_S_occipital_ant	29	7.22	0.012	0.047265306	46	-64	5
Encoding < Reproduction	Right_G_temp_sup-G_T_transv	29	7.053	0.013	0.05018	48	-14	7
Encoding < Reproduction	Right-Cerebral-White-Matter	29	6.823	0.014	0.051961538	29	-18	19
Encoding < Reproduction	Left_G_front_sup	29	6.805	0.014	0.051961538	-9	27	45

Encoding < Reproduction	Right_G_front_inf-Triangul	29	6.641	0.015	0.053611111	52	33	5
Encoding < Reproduction	Right_S_orbital_med-olfact	29	6.727	0.015	0.053611111	12	27	-19
Encoding < Reproduction	Left_G_insular_short	29	6.561	0.016	0.055142857	-38	10	-4
Encoding < Reproduction	Right_G_occipital_middle	29	6.497	0.016	0.055142857	45	-79	15
Encoding < Reproduction	Left-Pallidum	29	6.466	0.017	0.055610169	-21	-5	-1
Encoding < Reproduction	Right-Caudate	29	6.378	0.017	0.055610169	13	11	9
Encoding < Reproduction	Left_G_oc-temp_med-Lingual	29	6.363	0.017	0.055610169	-10	-73	-6
Encoding < Reproduction	Left-Cerebral-White-Matter	29	6.328	0.018	0.05695082	-29	-19	19
Encoding < Reproduction	Right_G_parietal_sup	29	6.287	0.018	0.05695082	25	-60	62
Encoding < Reproduction	Right-Accumbens-area	29	6.041	0.02	0.061269841	8	12	-8
Encoding < Reproduction	Left_G_and_S_cingul-Mid-Post	29	6.113	0.02	0.061269841	-7	-11	41
Encoding < Reproduction	Right-Putamen	29	5.956	0.021	0.063328125	26	2	0
Encoding < Reproduction	Left-vessel	29	5.899	0.022	0.064333333	-29	-3	-9
Encoding < Reproduction	Right-Cerebellum-White-Matter	29	5.864	0.022	0.064333333	19	-56	-33
Encoding < Reproduction	Right_G_and_S_subcentral	29	5.755	0.023	0.065279412	57	-8	14
Encoding < Reproduction	Right_S_calcarine	29	5.728	0.023	0.065279412	18	-65	7
Encoding < Reproduction	Right-VentralDC	29	5.636	0.024	0.067130435	10	-15	-10
Encoding < Reproduction	Left-Accumbens-area	29	5.616	0.025	0.067957746	-9	11	-8
Encoding < Reproduction	Right_S_circular_insula_ant	29	5.621	0.025	0.067957746	31	23	-10
Encoding < Reproduction	Right-Cerebellum-Cortex	29	5.532	0.026	0.069694444	24	-63	-37
Encoding < Reproduction	Left-Caudate	29	5.381	0.028	0.074027397	-14	10	10
Encoding < Reproduction	Left_S_temporal_transverse	29	5.256	0.029	0.074230769	-53	-23	5
Encoding < Reproduction	Brain-Stem	29	5.215	0.03	0.074230769	0	-31	-34
Encoding < Reproduction	Right_G_temp_sup-Plan_tempo	29	5.185	0.03	0.074230769	61	-28	20
Encoding < Reproduction	Right_Lat_Fis-ant-Vertical	29	5.179	0.03	0.074230769	48	23	10
Encoding < Reproduction	Right_S_pericallosal	29	5.229	0.03	0.074230769	6	-7	19
Encoding < Reproduction	Right_G_and_S_cingul-Ant	29	5.048	0.032	0.078177215	7	41	8
Encoding < Reproduction	Left_S_oc_sup_and_transversal	29	4.934	0.034	0.082025	-27	-84	21
Encoding < Reproduction	Left_S_front_inf	29	4.895	0.035	0.082378049	-41	27	20

Encoding < Reproduction	Left_S_oc-temp_med_and_Lingual	29	4.881	0.035	0.082378049	-28	-48	-11
Encoding < Reproduction	Right_G_orbital	29	4.731	0.038	0.088361446	29	38	-19
Encoding < Reproduction	Left-Cerebellum-White-Matter	29	4.692	0.039	0.089607143	-20	-57	-33
Encoding < Reproduction	Left-Cerebellum-Cortex	29	4.606	0.04	0.090823529	-25	-63	-37
Encoding < Reproduction	Left_S_calcarine	29	4.464	0.043	0.095390805	-15	-69	5
Encoding < Reproduction	Right_S_orbital_lateral	29	4.469	0.043	0.095390805	42	44	-3
Encoding < Reproduction	Left_G_cuneus	29	4.376	0.045	0.098693182	-4	-84	19
Encoding < Reproduction	Left_G_temp_sup-Plan_tempo	29	4.356	0.046	0.099752809	-59	-38	18
Encoding < Reproduction	Left_Lat_Fis-post	29	4.263	0.048	0.102933333	-41	-34	20
Encoding < Reproduction	Left_S_collat_transv_post	29	4.216	0.049	0.103923077	-24	-81	-7
Encoding < Reproduction	Right_G_and_S_cingul-Mid-Post	29	4.171	0.05	0.104891304	7	-8	43
Encoding < Reproduction	Left_G_occipital_middle	29	4.081	0.053	0.108819149	-39	-88	12
Encoding < Reproduction	Left_S_front_middle	29	4.001	0.055	0.111736842	-29	46	19
Encoding < Reproduction	Right_G_postcentral	29	3.951	0.056	0.112583333	47	-21	54
Encoding < Reproduction	Right-Thalamus-Proper	29	3.909	0.058	0.11422449	11	-17	7
Encoding < Reproduction	Left_Lat_Fis-ant-Horizont	29	3.887	0.058	0.11422449	-44	32	-4
Encoding < Reproduction	Right-choroid-plexus	29	3.839	0.06	0.116969697	20	-24	8
Encoding < Reproduction	Left-VentralDC	29	3.682	0.065	0.123305556	-11	-16	-10
Encoding < Reproduction	Right_S_collat_transv_ant	29	3.647	0.066	0.123305556	41	-18	-29
Encoding < Reproduction	Right_S_collat_transv_post	29	3.628	0.067	0.123305556	26	-79	-8
Encoding < Reproduction	Left_S_orbital_med-olfact	29	3.589	0.068	0.123305556	-13	25	-20
Encoding < Reproduction	Right_G_front_inf-Orbital	29	3.584	0.068	0.123305556	48	35	-11
Encoding < Reproduction	CC_Posterior	29	3.574	0.069	0.123305556	0	-35	17
Encoding < Reproduction	Left_G_subcallosal	29	3.565	0.069	0.123305556	-12	8	-13
Encoding < Reproduction	Left_S_oc-temp_lat	29	3.559	0.069	0.123305556	-44	-50	-14
Encoding < Reproduction	Right_G_cuneus	29	3.56	0.069	0.123305556	7	-80	21
Encoding < Reproduction	Right_G_temp_sup-Lateral	29	3.533	0.07	0.123944954	59	-4	-7
Encoding < Reproduction	Right_G_temporal_inf	29	3.49	0.072	0.126327273	55	-35	-25
Encoding < Reproduction	Left_S_orbital-H_Shaped	29	3.137	0.087	0.149919643	-26	40	-16

Encoding < Reproduction	Right_S_oc_middle_and_Lunatus	29	3.145	0.087	0.149919643	37	-79	10
Encoding < Reproduction	Left-Thalamus-Proper	29	3.066	0.091	0.155424779	-11	-19	7
Encoding < Reproduction	Left_G_temp_sup-Lateral	29	2.998	0.094	0.158358974	-58	-7	-7
Encoding < Reproduction	Left_S_pericallosal	29	2.983	0.095	0.158358974	-6	-4	17
Encoding < Reproduction	CSF	29	2.969	0.096	0.158358974	0	-19	10
Encoding < Reproduction	Right_G_oc-temp_med-Parahip	29	2.967	0.096	0.158358974	24	-13	-29
Encoding < Reproduction	Left_S_circular_insula_ant	29	2.914	0.098	0.160288136	-31	22	-10
Encoding < Reproduction	Left_G_and_S_cingul-Ant	29	2.798	0.105	0.170294118	-8	41	6
Encoding < Reproduction	Left-choroid-plexus	29	2.78	0.106	0.170483333	-22	-31	7
Encoding < Reproduction	Right_S_cingul-Marginalis	29	2.736	0.109	0.173859504	12	-37	50
Encoding < Reproduction	Left_G_Ins_lg_and_S_cent_ins	29	2.706	0.111	0.175598361	-38	-7	-2
Encoding < Reproduction	Right_Lat_Fis-post	29	2.589	0.118	0.185154472	42	-27	20
Encoding < Reproduction	Right_S_suborbital	29	2.557	0.121	0.186873016	5	39	-18
Encoding < Reproduction	Left_G_front_inf-Triangul	29	2.542	0.122	0.186873016	-52	31	4
Encoding < Reproduction	Right_S_temporal_sup	29	2.531	0.122	0.186873016	51	-38	10
Encoding < Reproduction	CC_Anterior	29	2.527	0.123	0.18692126	0	25	5
Encoding < Reproduction	Left_S_cingul-Marginalis	29	2.468	0.127	0.191492188	-13	-38	49
Encoding < Reproduction	Left_S_collat_transv_ant	29	2.437	0.129	0.193	-41	-20	-30
Encoding < Reproduction	Left_S_orbital_lateral	29	2.371	0.134	0.198938462	-42	47	-5
Encoding < Reproduction	Right_G_subcallosal	29	2.356	0.136	0.200310606	5	12	-13
Encoding < Reproduction	Left_G_oc-temp_med-Parahip	29	2.345	0.137	0.200310606	-23	-16	-26
Encoding < Reproduction	Right_S_front_sup	29	2.306	0.14	0.203157895	23	25	45
Encoding < Reproduction	Left_G_front_middle	29	2.241	0.145	0.205678832	-38	34	34
Encoding < Reproduction	Right_G_Ins_lg_and_S_cent_ins	29	2.246	0.145	0.205678832	39	-4	-3
Encoding < Reproduction	Right_G_pariet_inf-Angular	29	2.238	0.145	0.205678832	50	-58	42
Encoding < Reproduction	Right_G_temp_sup-Plan_polar	29	2.237	0.146	0.205678832	36	9	-20
Encoding < Reproduction	Right_G_occipital_sup	29	2.212	0.148	0.206985507	20	-89	31
Encoding < Reproduction	Right_S_temporal_inf	29	2.19	0.15	0.208273381	55	-33	-16
Encoding < Reproduction	Right_Pole_temporal	29	2.165	0.152	0.209542857	32	9	-42

Encoding < Reproduction	Right_S_circular_insula_inf	29	2.094	0.159	0.217638298	42	-8	-7
Encoding < Reproduction	Right_Pole_occipital	29	1.96	0.172	0.233774648	20	-95	-3
Encoding < Reproduction	Left_G_front_inf-Orbital	29	1.845	0.185	0.245517007	-46	29	-10
Encoding < Reproduction	Optic-Chiasm	29	1.83	0.187	0.245517007	-1	1	-19
Encoding < Reproduction	Left_S_circular_insula_inf	29	1.822	0.187	0.245517007	-41	-12	-5
Encoding < Reproduction	Left_G_temp_sup-Plan_polar	29	1.808	0.189	0.245753333	-41	8	-20
Encoding < Reproduction	Right-Inf-Lat-Vent	29	1.799	0.19	0.245753333	31	-14	-18
Encoding < Reproduction	Right-Hippocampus	29	1.795	0.191	0.245753333	26	-22	-14
Encoding < Reproduction	Right_G_temporal_middle	29	1.729	0.199	0.254350993	62	-23	-14
Encoding < Reproduction	Right_S_parieto_occipital	29	1.682	0.205	0.260296053	18	-66	28
Encoding < Reproduction	Left_G_temporal_inf	29	1.641	0.21	0.264901961	-55	-40	-23
Encoding < Reproduction	Right_G_rectus	29	1.577	0.219	0.274461039	4	38	-24
Encoding < Reproduction	Left_G_orbital	29	1.515	0.228	0.283896774	-31	35	-19
Encoding < Reproduction	Left_Pole_temporal	29	1.491	0.232	0.287025641	-33	6	-42
Encoding < Reproduction	Left_G_and_S_paracentral	29	1.44	0.24	0.293164557	-8	-36	68
Encoding < Reproduction	Left_G_rectus	29	1.438	0.24	0.293164557	-5	36	-23
Encoding < Reproduction	Left_S_suborbital	29	1.343	0.256	0.310742138	-8	40	-14
Encoding < Reproduction	Right_G_and_S_frontomargin	29	1.154	0.292	0.352225	23	62	-11
Encoding < Reproduction	Left_S_temporal_inf	29	1.109	0.301	0.355587879	-55	-34	-16
Encoding < Reproduction	Right-Amygdala	29	1.095	0.304	0.355587879	23	-4	-20
Encoding < Reproduction	Left_G_and_S_frontomargin	29	1.097	0.304	0.355587879	-24	61	-10
Encoding < Reproduction	Left_S_parieto_occipital	29	1.097	0.304	0.355587879	-15	-68	25
Encoding < Reproduction	Left_S_oc_middle_and_Lunatus	29	0.997	0.326	0.379024096	-33	-87	8
Encoding < Reproduction	Left_G_occipital_sup	29	0.955	0.337	0.389467066	-15	-92	29
Encoding < Reproduction	Left-Hippocampus	29	0.913	0.347	0.398636905	-26	-23	-14
Encoding < Reproduction	Right_G_and_S_paracentral	29	0.765	0.389	0.444242604	7	-35	69
Encoding < Reproduction	Left_S_temporal_sup	29	0.699	0.41	0.465470588	-51	-43	8
Encoding < Reproduction	Left-Amygdala	29	0.686	0.414	0.467263158	-24	-5	-20
Encoding < Reproduction	Left-Inf-Lat-Vent	29	0.605	0.443	0.49532948	-33	-20	-15

Encoding < Reproduction	Left-Lateral-Ventricle	29	0.603	0.444	0.49532948	-15	-17	14
Encoding < Reproduction	CC_Mid_Anterior	29	0.528	0.473	0.524649425	0	13	19
Encoding < Reproduction	WM-hypointensities	29	0.509	0.481	0.530474286	-4	-14	19
Encoding < Reproduction	Left_G_temporal_middle	29	0.493	0.488	0.535136364	-63	-25	-13
Encoding < Reproduction	Left_S_front_sup	29	0.487	0.491	0.535384181	-24	22	46
Encoding < Reproduction	Left_S_interm_prim-Jensen	29	0.41	0.527	0.565061111	-52	-41	46
Encoding < Reproduction	Right_S_central	29	0.359	0.554	0.590729282	37	-17	47
Encoding < Reproduction	Left_Pole_occipital	29	0.255	0.617	0.650715847	-16	-101	-4
Encoding < Reproduction	Right_S_subparietal	29	0.211	0.649	0.680744565	9	-48	38
Encoding < Reproduction	Right_G_and_S_transv_frontopol	29	0.187	0.668	0.696886486	17	68	0
Encoding < Reproduction	Right_G_precuneus	29	0.144	0.707	0.733607527	6	-60	47
Encoding < Reproduction	Right_G_cingul-Post-dorsal	29	0.089	0.768	0.788425532	3	-36	34
Encoding < Reproduction	Left_G_pariet_inf-Angular	29	0.07	0.794	0.810804233	-44	-69	41
Encoding < Reproduction	Right_G_cingul-Post-ventral	29	0.052	0.822	0.834978947	8	-48	7
Encoding < Reproduction	Left_G_cingul-Post-ventral	29	0.046	0.832	0.840712042	-8	-48	6
Encoding < Reproduction	Left_S_subparietal	29	0.01	0.922	0.922	-10	-49	38
Encoding > Reproduction	Left_G_and_S_transv_frontopol	29	0.444	0.511	0.55096648	-17	67	-3
Encoding > Reproduction	Left_G_cingul-Post-dorsal	29	0.306	0.584	0.619296703	-3	-38	32
Encoding > Reproduction	4th-Ventricle	29	0.119	0.733	0.756518717	-1	-46	-35
Encoding > Reproduction	Left_G_precuneus	29	0.041	0.841	0.845380208	-5	-60	45

Table S8 – ROI-based repeated-measures ANOVA results comparing Scream vs. Encoding in Threat condition.

The table reports regions showing Regressor effects (Scream vs. Encoding), listing the direction of the contrast, ROI label, degrees of freedom (df), F-values, uncorrected p-values, and FDR-corrected p-values, together with MNI coordinates (X, Y, Z). ROIs are labeled according to the Destrieux atlas nomenclature.

Constrast	ROI	df	F-value	p-value	p-value (FDR-corr)	X	Y	Z
Scream > Encoding	Right_G_front_inf-Triangul	29	45.42	2.14E-07	3.90E-05	52	33	5
Scream > Encoding	Right_S_temporal_sup	29	42.297	4.04E-07	3.90E-05	51	-38	10
Scream > Encoding	Left_G_temp_sup-Plan_polar	29	34.654	2.17E-06	1.40E-04	-41	8	-20
Scream > Encoding	Right_G_temp_sup-Lateral	29	33.433	2.90E-06	1.40E-04	59	-4	-7
Scream > Encoding	Right-Amygdala	29	31.407	4.74E-06	1.79E-04	23	-4	-20
Scream > Encoding	Left_G_front_inf-Triangul	29	30.347	6.17E-06	1.79E-04	-52	31	4
Scream > Encoding	Left_G_temp_sup-Lateral	29	29.623	7.42E-06	1.79E-04	-58	-7	-7
Scream > Encoding	Right_S_circular_insula_inf	29	28.761	9.25E-06	1.98E-04	42	-8	-7
Scream > Encoding	Left_S_circular_insula_inf	29	27.86	1.17E-05	2.26E-04	-41	-12	-5
Scream > Encoding	Right_G_temporal_middle	29	24.472	2.94E-05	4.73E-04	62	-23	-14
Scream > Encoding	Left_G_front_inf-Orbital	29	23.263	4.15E-05	5.34E-04	-46	29	-10
Scream > Encoding	Left_Lat_Fis-ant-Vertical	29	22.73	4.84E-05	5.84E-04	-48	21	11
Scream > Encoding	Left_Lat_Fis-ant-Horizont	29	21.825	6.31E-05	7.00E-04	-44	32	-4
Scream > Encoding	Left_S_interm_prim-Jensen	29	21.71	6.53E-05	7.00E-04	-52	-41	46
Scream > Encoding	Right_G_temp_sup-Plan_polar	29	20.991	8.10E-05	8.23E-04	36	9	-20
Scream > Encoding	Left_S_temporal_sup	29	20.59	9.15E-05	8.83E-04	-51	-43	8
Scream > Encoding	Left-Amygdala	29	19.582	1.25E-04	0.00114881	-24	-5	-20
Scream > Encoding	Right_G_front_inf-Orbital	29	17.407	2.50E-04	0.002097826	48	35	-11
Scream > Encoding	Left_S_circular_insula_ant	29	16.416	3.48E-04	0.00273288	-31	22	-10
Scream > Encoding	Right-VentralDC	29	15.919	4.11E-04	0.002832964	10	-15	-10
Scream > Encoding	Left_S_subparietal	29	15.948	4.07E-04	0.002832964	-10	-49	38
Scream > Encoding	Right_G_Ins_lg_and_S_cent_ins	29	16.025	3.97E-04	0.002832964	39	-4	-3
Scream > Encoding	Left-Hippocampus	29	15.095	5.46E-04	0.00339929	-26	-23	-14

Scream > Encoding	Right_Lat_Fis-ant-Horizont	29	15.176	5.31E-04	0.00339929	41	29	0
Scream > Encoding	Left_G_insular_short	29	13.471	9.71E-04	0.005078947	-38	10	-4
Scream > Encoding	Left_G_temporal_middle	29	12.391	0.001	0.005078947	-63	-25	-13
Scream > Encoding	Left-VentralDC	29	11.786	0.002	0.008391304	-11	-16	-10
Scream > Encoding	Right-Hippocampus	29	12.203	0.002	0.008391304	26	-22	-14
Scream > Encoding	Left_G_and_S_cingul-Ant	29	11.714	0.002	0.008391304	-8	41	6
Scream > Encoding	Left_G_Ins_lg_and_S_cent_ins	29	11.855	0.002	0.008391304	-38	-7	-2
Scream > Encoding	Right_G_front_inf-Opercular	29	12.023	0.002	0.008391304	51	14	9
Scream > Encoding	Left_G_front_inf-Opercular	29	10.911	0.003	0.010924528	-51	12	10
Scream > Encoding	Left_G_orbital	29	10.569	0.003	0.010924528	-31	35	-19
Scream > Encoding	Left_G_temp_sup-Plan_tempo	29	10.25	0.003	0.010924528	-59	-38	18
Scream > Encoding	Right_G_front_sup	29	10.643	0.003	0.010924528	10	28	47
Scream > Encoding	Right-Inf-Lat-Vent	29	9.616	0.004	0.013084746	31	-14	-18
Scream > Encoding	Left_S_pericallosal	29	9.541	0.004	0.013084746	-6	-4	17
Scream > Encoding	Right_G_and_S_transv_frontopol	29	9.682	0.004	0.013084746	17	68	0
Scream > Encoding	Right_S_subparietal	29	9.64	0.004	0.013084746	9	-48	38
Scream > Encoding	Left-Inf-Lat-Vent	29	9.047	0.005	0.015564516	-33	-20	-15
Scream > Encoding	Optic-Chiasm	29	9.253	0.005	0.015564516	-1	1	-19
Scream > Encoding	Left_G_subcallosal	29	9.115	0.005	0.015564516	-12	8	-13
Scream > Encoding	Right_G_and_S_cingul-Ant	29	8.45	0.007	0.020164179	7	41	8
Scream > Encoding	Left_G_cingul-Post-ventral	29	8.065	0.008	0.022376812	-8	-48	6
Scream > Encoding	Left_G_pariet_inf-Angular	29	8.044	0.008	0.022376812	-44	-69	41
Scream > Encoding	Right_S_interm_prim-Jensen	29	7.579	0.01	0.027571429	52	-41	46
Scream > Encoding	Right_G_pariet_inf-Angular	29	7.367	0.011	0.029486111	50	-58	42
Scream > Encoding	Right_Lat_Fis-ant-Vertical	29	7.266	0.012	0.03088	48	23	10
Scream > Encoding	Right_S_circular_insula_ant	29	7.197	0.012	0.03088	31	23	-10
Scream > Encoding	Right_S_pericallosal	29	7.151	0.012	0.03088	6	-7	19
Scream > Encoding	Left_S_orbital_med-olfact	29	6.742	0.015	0.038092105	-13	25	-20
Scream > Encoding	Left_G_and_S_transv_frontopol	29	6.488	0.016	0.039589744	-17	67	-3

Scream > Encoding	Right_S_front_inf	29	6.488	0.016	0.039589744	40	27	23
Scream > Encoding	Left_G_precuneus	29	6.454	0.017	0.0410125	-5	-60	45
Scream > Encoding	Left-Pallidum	29	6.211	0.019	0.044719512	-21	-5	-1
Scream > Encoding	Right_G_insular_short	29	6.111	0.02	0.046506024	38	12	-5
Scream > Encoding	Left_S_collat_transv_ant	29	5.654	0.024	0.055142857	-41	-20	-30
Scream > Encoding	Right_G_temp_sup-Plan_tempo	29	5.621	0.025	0.056104651	61	-28	20
Scream > Encoding	Right_G_subcallosal	29	5.18	0.03	0.066551724	5	12	-13
Scream > Encoding	Left_S_parieto_occipital	29	5.059	0.032	0.070181818	-15	-68	25
Scream > Encoding	Right_S_orbital-H_Shaped	29	5.013	0.033	0.071561798	25	40	-16
Scream > Encoding	Right_G_cingul-Post-ventral	29	4.876	0.035	0.074230769	8	-48	7
Scream > Encoding	Right_G_precuneus	29	4.891	0.035	0.074230769	6	-60	47
Scream > Encoding	Left_G_front_middle	29	4.308	0.047	0.0965	-38	34	34
Scream > Encoding	Right_G_cingul-Post-dorsal	29	4.28	0.048	0.097515789	3	-36	34
Scream > Encoding	CC_Central	29	4.217	0.049	0.098510417	0	-2	22
Scream > Encoding	Right_G_orbital	29	4.112	0.052	0.103463918	29	38	-19
Scream > Encoding	CSF	29	3.951	0.056	0.109171717	0	-19	10
Scream > Encoding	Right_G_and_S_cingul-Mid-Post	29	3.96	0.056	0.109171717	7	-8	43
Scream > Encoding	Left_S_front_inf	29	3.771	0.062	0.117313725	-41	27	20
Scream > Encoding	Left_S_orbital-H_Shaped	29	3.735	0.063	0.118048544	-26	40	-16
Scream > Encoding	Left_G_oc-temp_med-Parahip	29	3.629	0.067	0.124336538	-23	-16	-26
Scream > Encoding	Right_S_orbital_med-olfact	29	3.554	0.069	0.126828571	12	27	-19
Scream > Encoding	Right-Thalamus-Proper	29	3.423	0.074	0.133476636	11	-17	7
Scream > Encoding	Left_G_cingul-Post-dorsal	29	3.383	0.076	0.135814815	-3	-38	32
Scream > Encoding	Right-Pallidum	29	3.359	0.077	0.13633945	21	-4	-2
Scream > Encoding	Right-Putamen	29	3.212	0.084	0.146054054	26	2	0
Scream > Encoding	Left-Thalamus-Proper	29	2.672	0.113	0.193	-11	-19	7
Scream > Encoding	Left_G_front_sup	29	2.651	0.114	0.193	-9	27	45
Scream > Encoding	Left_S_cingul-Marginalis	29	2.676	0.113	0.193	-13	-38	49
Scream > Encoding	Right-Caudate	29	2.609	0.117	0.196356522	13	11	9

Scream > Encoding	Left-Putamen	29	2.515	0.124	0.204449153	-26	1	0
Scream > Encoding	Left_S_suborbital	29	2.372	0.134	0.217327731	-8	40	-14
Scream > Encoding	Left_G_and_S_cingul-Mid-Post	29	2.303	0.14	0.225166667	-7	-11	41
Scream > Encoding	Left-Accumbens-area	29	2.211	0.148	0.230354839	-9	11	-8
Scream > Encoding	Right_S_orbital_lateral	29	2.242	0.145	0.230354839	42	44	-3
Scream > Encoding	Right_S_parieto_occipital	29	2.092	0.159	0.243547619	18	-66	28
Scream > Encoding	Right_S_front_sup	29	2.063	0.162	0.246188976	23	25	45
Scream > Encoding	Left-Caudate	29	1.989	0.169	0.254341085	-14	10	10
Scream > Encoding	Right_G_and_S_frontomargin	29	1.984	0.17	0.254341085	23	62	-11
Scream > Encoding	Right_S_suborbital	29	1.895	0.179	0.265746154	5	39	-18
Scream > Encoding	Right_S_cingul-Marginalis	29	1.852	0.184	0.271083969	12	-37	50
Scream > Encoding	Left_S_orbital_lateral	29	1.815	0.188	0.274878788	-42	47	-5
Scream > Encoding	Right_G_front_middle	29	1.787	0.192	0.278616541	38	36	33
Scream > Encoding	Left_G_temporal_inf	29	1.635	0.211	0.299433824	-55	-40	-23
Scream > Encoding	Right_Pole_temporal	29	1.646	0.21	0.299433824	32	9	-42
Scream > Encoding	Left_G_pariet_inf-Supramar	29	1.608	0.215	0.302883212	-60	-38	34
Scream > Encoding	Left_S_front_sup	29	1.431	0.241	0.337050725	-24	22	46
Scream > Encoding	Left-Cerebellum-White-Matter	29	1.399	0.246	0.339128571	-20	-57	-33
Scream > Encoding	Right_S_precentral-inf-part	29	1.404	0.246	0.339128571	45	7	30
Scream > Encoding	Right_G_rectus	29	1.135	0.295	0.400950704	4	38	-24
Scream > Encoding	Right_S_temporal_inf	29	1.138	0.295	0.400950704	55	-33	-16
Scream > Encoding	Right_G_temporal_inf	29	0.985	0.329	0.444034965	55	-35	-25
Scream > Encoding	Left-Cerebellum-Cortex	29	0.958	0.336	0.447227586	-25	-63	-37
Scream > Encoding	Left_G_and_S_frontomargin	29	0.958	0.336	0.447227586	-24	61	-10
Scream > Encoding	Right-Accumbens-area	29	0.779	0.385	0.50547619	8	12	-8
Scream > Encoding	3rd-Ventricle	29	0.707	0.407	0.523673333	0	-11	-3
Scream > Encoding	CC_Mid_Posterior	29	0.72	0.403	0.523673333	0	-18	22
Scream > Encoding	Left_Pole_temporal	29	0.707	0.407	0.523673333	-33	6	-42
Scream > Encoding	Left_G_and_S_cingul-Mid-Ant	29	0.63	0.434	0.551065789	-7	16	35

Scream > Encoding	CC_Posterior	29	0.57	0.457	0.576477124	0	-35	17
Scream > Encoding	Left_S_temporal_transverse	29	0.552	0.464	0.581506494	-53	-23	5
Scream > Encoding	CC_Mid_Anterior	29	0.499	0.486	0.605148387	0	13	19
Scream > Encoding	Left_S_front_middle	29	0.418	0.523	0.63885443	-29	46	19
Scream > Encoding	Right_S_collat_transv_ant	29	0.411	0.527	0.639691824	41	-18	-29
Scream > Encoding	Left_G_temp_sup-G_T_transv	29	0.384	0.54	0.651375	-49	-18	8
Scream > Encoding	Left_G_rectus	29	0.351	0.558	0.668906832	-5	36	-23
Scream > Encoding	Right_G_pariet_inf-Supramar	29	0.289	0.595	0.704509202	59	-29	36
Scream > Encoding	CC_Anterior	29	0.241	0.627	0.728981928	0	25	5
Scream > Encoding	Right_G_oc-temp_med-Parahip	29	0.229	0.636	0.735017964	24	-13	-29
Scream > Encoding	Right_G_and_S_subcentral	29	0.216	0.646	0.738881657	57	-8	14
Scream > Encoding	Right_G_and_S_cingul-Mid-Ant	29	0.214	0.647	0.738881657	7	17	35
Scream > Encoding	Right-choroid-plexus	29	0.205	0.654	0.742482353	20	-24	8
Scream > Encoding	Brain-Stem	29	0.198	0.66	0.743947674	0	-31	-34
Scream > Encoding	Right_Lat_Fis-post	29	0.173	0.68	0.758612717	42	-27	20
Scream > Encoding	Right_S_front_middle	29	0.131	0.72	0.794057143	30	47	19
Scream > Encoding	Left_G_cuneus	29	0.093	0.763	0.827297753	-4	-84	19
Scream > Encoding	Right_S_circular_insula_sup	29	0.088	0.768	0.828067039	37	9	9
Scream > Encoding	Right_G_temp_sup-G_T_transv	29	0.069	0.794	0.851344444	48	-14	7
Scream > Encoding	Left_S_temporal_inf	29	0.06	0.808	0.861569061	-55	-34	-16
Scream > Encoding	Right_G_and_S_paracentral	29	0.05	0.824	0.864304348	7	-35	69
Scream > Encoding	Left-Cerebral-White-Matter	29	0.013	0.911	0.934526316	-29	-19	19
Scream > Encoding	Left_Lat_Fis-post	29	0.01	0.92	0.934526316	-41	-34	20
Scream > Encoding	Left-choroid-plexus	29	0.009	0.925	0.934685864	-22	-31	7
Scream < Encoding	Left_G_postcentral	29	30.135	6.51E-06	1.79E-04	-47	-25	55
Scream < Encoding	Right_S_oc_middle_and_Lunatus	29	27.161	1.41E-05	2.47E-04	37	-79	10
Scream < Encoding	Right_S_oc_sup_and_transversal	29	23.835	3.52E-05	5.23E-04	30	-79	23
Scream < Encoding	Left_S_postcentral	29	23.514	3.86E-05	5.32E-04	-42	-33	46
Scream < Encoding	Right_G_and_S_occipital_inf	29	19.437	1.31E-04	0.001149227	43	-76	-12

Scream < Encoding	Left_S_precentral-sup-part	29	16.362	3.54E-04	0.00273288	-29	-10	56
Scream < Encoding	Left_S_occipital_ant	29	15.184	5.29E-04	0.00339929	-41	-70	7
Scream < Encoding	Right_S_occipital_ant	29	14.582	6.53E-04	0.003938406	46	-64	5
Scream < Encoding	Left_S_central	29	12.432	0.001	0.005078947	-39	-19	46
Scream < Encoding	Left_S_oc_sup_and_transversal	29	13.591	0.00093	0.005078947	-27	-84	21
Scream < Encoding	Right_S_collat_transv_post	29	12.637	0.001	0.005078947	26	-79	-8
Scream < Encoding	Left_G_and_S_occipital_inf	29	12.141	0.002	0.008391304	-39	-83	-10
Scream < Encoding	Left_G_precentral	29	12.068	0.002	0.008391304	-43	-9	53
Scream < Encoding	Left_S_oc_middle_and_Lunatus	29	11.832	0.002	0.008391304	-33	-87	8
Scream < Encoding	Right_G_occipital_middle	29	10.242	0.003	0.010924528	45	-79	15
Scream < Encoding	Right_S_postcentral	29	10.308	0.003	0.010924528	38	-30	49
Scream < Encoding	Right_G_oc-temp_lat-fusifor	29	9.591	0.004	0.013084746	35	-48	-21
Scream < Encoding	Left_S_intrapariet_and_P_trans	29	8.992	0.006	0.01809375	-32	-58	43
Scream < Encoding	Right_S_precentral-sup-part	29	8.624	0.006	0.01809375	29	-9	56
Scream < Encoding	Right_S_intrapariet_and_P_trans	29	8.573	0.007	0.020164179	30	-56	47
Scream < Encoding	Right_G_postcentral	29	7.428	0.011	0.029486111	47	-21	54
Scream < Encoding	Left_S_oc-temp_lat	29	6.389	0.017	0.0410125	-44	-50	-14
Scream < Encoding	Left_S_collat_transv_post	29	6.332	0.018	0.042888889	-24	-81	-7
Scream < Encoding	Right_S_oc-temp_lat	29	4.6	0.04	0.083913043	45	-47	-15
Scream < Encoding	Left_G_occipital_middle	29	4.57	0.041	0.085086022	-39	-88	12
Scream < Encoding	Right_G_occipital_sup	29	3.83	0.06	0.1158	20	-89	31
Scream < Encoding	Right_Pole_occipital	29	3.805	0.061	0.116564356	20	-95	-3
Scream < Encoding	Right_S_oc-temp_med_and_Lingual	29	3.441	0.074	0.133476636	28	-48	-11
Scream < Encoding	Left_G_parietal_sup	29	3.291	0.08	0.140363636	-26	-61	59
Scream < Encoding	Left_G_occipital_sup	29	2.575	0.119	0.197991379	-15	-92	29
Scream < Encoding	Left_Pole_occipital	29	2.49	0.125	0.204449153	-16	-101	-4
Scream < Encoding	Left_G_oc-temp_lat-fusifor	29	2.217	0.147	0.230354839	-36	-55	-19
Scream < Encoding	Right_G_precentral	29	2.213	0.148	0.230354839	42	-7	53
Scream < Encoding	Right_G_parietal_sup	29	2.132	0.155	0.23932	25	-60	62

Scream < Encoding	WM-hypointensities	29	0.917	0.346	0.457383562	-4	-14	19
Scream < Encoding	4th-Ventricle	29	0.679	0.416	0.531708609	-1	-46	-35
Scream < Encoding	Right_S_calcarine	29	0.441	0.512	0.633435897	18	-65	7
Scream < Encoding	Right_S_central	29	0.418	0.523	0.63885443	37	-17	47
Scream < Encoding	Right_G_oc-temp_med-Lingual	29	0.318	0.577	0.68741358	10	-67	-4
Scream < Encoding	Right-Lateral-Ventricle	29	0.251	0.62	0.725212121	14	-14	14
Scream < Encoding	Left_G_and_S_subcentral	29	0.255	0.617	0.725212121	-57	-11	15
Scream < Encoding	Right-Cerebellum-Cortex	29	0.193	0.663	0.743947674	24	-63	-37
Scream < Encoding	Right-Cerebellum-White-Matter	29	0.156	0.695	0.770890805	19	-56	-33
Scream < Encoding	Left_G_oc-temp_med-Lingual	29	0.121	0.731	0.801607955	-10	-73	-6
Scream < Encoding	Left-Lateral-Ventricle	29	0.095	0.76	0.827297753	-15	-17	14
Scream < Encoding	Left_G_and_S_paracentral	29	0.054	0.817	0.862699454	-8	-36	68
Scream < Encoding	Left_S_calcarine	29	0.054	0.818	0.862699454	-15	-69	5
Scream < Encoding	Right_S_temporal_transverse	29	0.033	0.857	0.894059459	53	-18	7
Scream < Encoding	Left_S_oc-temp_med_and_Lingual	29	0.017	0.898	0.931795699	-28	-48	-11
Scream < Encoding	Left_S_circular_insula_sup	29	0.01	0.919	0.934526316	-36	6	11
Scream < Encoding	Left_S_precentral-inf-part	29	0.013	0.912	0.934526316	-45	4	30
Scream < Encoding	Right_G_cuneus	29	0.004	0.951	0.955953125	7	-80	21
Scream < Encoding	Right-Cerebral-White-Matter	29	0.001	0.974	0.974	29	-18	19

Table S9 – ROI-based repeated-measures ANOVA results comparing Scream vs. Reproduction in Threat condition.

The table reports regions showing Regressor effects (Scream vs. Reproduction), listing the direction of the contrast, ROI label, degrees of freedom (df), F-values, uncorrected p-values, and FDR-corrected p-values, together with MNI coordinates (X, Y, Z). ROIs are labeled according to the Destrieux atlas nomenclature.

Contrast	ROI	df	F-value	p-value	p-value (FDR-corr)	X	Y	Z
Scream > Reproduction	Right_G_front_inf-Triangul	29	28.191	1.07E-05	6.22E-04	52	33	5
Scream > Reproduction	Right_S_temporal_sup	29	22.984	4.49E-05	0.0012159	51	-38	10
Scream > Reproduction	Left_G_temp_sup-Plan_polar	29	21.945	6.09E-05	0.001305967	-41	8	-20
Scream > Reproduction	Left_G_front_inf-Triangul	29	21.021	8.03E-05	0.00154979	-52	31	4
Scream > Reproduction	Right_G_temp_sup-Lateral	29	18.351	1.84E-04	0.002564143	59	-4	-7
Scream > Reproduction	Left_G_temp_sup-Lateral	29	17.831	2.18E-04	0.002804933	-58	-7	-7
Scream > Reproduction	Right-Amygdala	29	17.313	2.58E-04	0.002974471	23	-4	-20
Scream > Reproduction	Left_G_front_inf-Orbital	29	15.597	4.59E-04	0.004662474	-46	29	-10
Scream > Reproduction	Right_G_temporal_middle	29	14.997	5.65E-04	0.005192619	62	-23	-14
Scream > Reproduction	Left_S_interm_prim-Jensen	29	13.832	8.53E-04	0.006859542	-52	-41	46
Scream > Reproduction	Left_Lat_Fis-ant-Vertical	29	12.99	0.001	0.006892857	-48	21	11
Scream > Reproduction	Left_S_temporal_sup	29	12.897	0.001	0.006892857	-51	-43	8
Scream > Reproduction	Left-Amygdala	29	11.855	0.002	0.011352941	-24	-5	-20
Scream > Reproduction	Left_S_subparietal	29	12.131	0.002	0.011352941	-10	-49	38
Scream > Reproduction	Left-Hippocampus	29	10.664	0.003	0.015236842	-26	-23	-14
Scream > Reproduction	Left_Lat_Fis-ant-Horizont	29	10.584	0.003	0.015236842	-44	32	-4
Scream > Reproduction	Left_S_circular_insula_inf	29	10.485	0.003	0.015236842	-41	-12	-5
Scream > Reproduction	Right_S_circular_insula_inf	29	10.847	0.003	0.015236842	42	-8	-7
Scream > Reproduction	Right_G_temp_sup-Plan_polar	29	10.1	0.004	0.019794872	36	9	-20
Scream > Reproduction	Right_G_front_inf-Orbital	29	9.144	0.005	0.024125	48	35	-11
Scream > Reproduction	Left_G_temporal_middle	29	8.553	0.007	0.031418605	-63	-25	-13
Scream > Reproduction	Right-VentralDC	29	8.104	0.008	0.034311111	10	-15	-10
Scream > Reproduction	Right_G_Ins_lg_and_S_cent_ins	29	8.033	0.008	0.034311111	39	-4	-3

Scream > Reproduction	Right-Hippocampus	29	7.563	0.01	0.040208333	26	-22	-14
Scream > Reproduction	Left_G_and_S_cingul-Ant	29	7.451	0.011	0.041627451	-8	41	6
Scream > Reproduction	Left_G_cingul-Post-ventral	29	7.286	0.011	0.041627451	-8	-48	6
Scream > Reproduction	Left_G_orbital	29	7.331	0.011	0.041627451	-31	35	-19
Scream > Reproduction	Right_G_and_S_transv_frontopol	29	7.25	0.012	0.043698113	17	68	0
Scream > Reproduction	Right_S_subparietal	29	7.139	0.012	0.043698113	9	-48	38
Scream > Reproduction	Left_G_pariet_inf-Angular	29	6.44	0.017	0.056568966	-44	-69	41
Scream > Reproduction	Left_S_pericallosal	29	6.351	0.017	0.056568966	-6	-4	17
Scream > Reproduction	Left-VentralDC	29	5.923	0.021	0.068694915	-11	-16	-10
Scream > Reproduction	Left_G_Ins_lg_and_S_cent_ins	29	5.888	0.022	0.070766667	-38	-7	-2
Scream > Reproduction	Left-Inf-Lat-Vent	29	5.737	0.023	0.072770492	-33	-20	-15
Scream > Reproduction	Left_G_and_S_transv_frontopol	29	5.703	0.024	0.07352381	-17	67	-3
Scream > Reproduction	Right-Inf-Lat-Vent	29	5.538	0.026	0.0772	31	-14	-18
Scream > Reproduction	Left_G_precuneus	29	5.507	0.026	0.0772	-5	-60	45
Scream > Reproduction	Right_Lat_Fis-ant-Horizont	29	5.193	0.03	0.08641791	41	29	0
Scream > Reproduction	Left_G_subcallosal	29	5.048	0.032	0.088228571	-12	8	-13
Scream > Reproduction	Right_G_front_sup	29	5.102	0.032	0.088228571	10	28	47
Scream > Reproduction	Right_G_pariet_inf-Angular	29	5.1	0.032	0.088228571	50	-58	42
Scream > Reproduction	Left_G_temp_sup-Plan_tempo	29	5.009	0.033	0.089704225	-59	-38	18
Scream > Reproduction	Right_G_front_inf-Opercular	29	4.93	0.034	0.089890411	51	14	9
Scream > Reproduction	Left_G_insular_short	29	4.341	0.046	0.113820513	-38	10	-4
Scream > Reproduction	Right_G_cingul-Post-ventral	29	4.213	0.049	0.1182125	8	-48	7
Scream > Reproduction	Right_S_pericallosal	29	4.174	0.05	0.119135802	6	-7	19
Scream > Reproduction	Right_G_and_S_cingul-Ant	29	4.135	0.051	0.120036585	7	41	8
Scream > Reproduction	Optic-Chiasm	29	4.086	0.053	0.123240964	-1	1	-19
Scream > Reproduction	Left_S_circular_insula_ant	29	3.816	0.06	0.136235294	-31	22	-10
Scream > Reproduction	Right_S_front_inf	29	3.847	0.06	0.136235294	40	27	23
Scream > Reproduction	Left_G_front_inf-Opercular	29	3.717	0.064	0.143627907	-51	12	10
Scream > Reproduction	Right_G_precuneus	29	3.596	0.068	0.150850575	6	-60	47

Scream > Reproduction	Left_S_collat_transv_ant	29	3.567	0.069	0.151329545	-41	-20	-30
Scream > Reproduction	Right_S_interm_prim-Jensen	29	3.546	0.07	0.151797753	52	-41	46
Scream > Reproduction	Right_G_cingul-Post-dorsal	29	3.416	0.075	0.160833333	3	-36	34
Scream > Reproduction	Left_S_parieto_occipital	29	3.313	0.079	0.165728261	-15	-68	25
Scream > Reproduction	Left_G_cingul-Post-dorsal	29	3.16	0.086	0.176574468	-3	-38	32
Scream > Reproduction	Left_S_orbital_med-olfact	29	2.919	0.098	0.19903125	-13	25	-20
Scream > Reproduction	Right_S_orbital-H_Shaped	29	2.816	0.104	0.206927835	25	40	-16
Scream > Reproduction	Left-Pallidum	29	2.589	0.118	0.230040404	-21	-5	-1
Scream > Reproduction	Left_G_front_middle	29	2.567	0.12	0.2316	-38	34	34
Scream > Reproduction	CC_Central	29	2.498	0.125	0.238861386	0	-2	22
Scream > Reproduction	Left_G_oc-temp_med-Parahip	29	2.427	0.13	0.245980392	-23	-16	-26
Scream > Reproduction	Left_S_orbital-H_Shaped	29	2.343	0.137	0.256708738	-26	40	-16
Scream > Reproduction	Right_G_temp_sup-Plan_tempo	29	2.119	0.156	0.2895	61	-28	20
Scream > Reproduction	Right_G_orbital	29	2.046	0.163	0.299609524	29	38	-19
Scream > Reproduction	CSF	29	1.976	0.17	0.309528302	0	-19	10
Scream > Reproduction	Right_S_circular_insula_ant	29	1.714	0.201	0.359194444	31	23	-10
Scream > Reproduction	Right_G_subcallosal	29	1.659	0.208	0.368293578	5	12	-13
Scream > Reproduction	Right_Lat_Fis-ant-Vertical	29	1.608	0.215	0.377227273	48	23	10
Scream > Reproduction	Left_S_front_inf	29	1.576	0.219	0.380783784	-41	27	20
Scream > Reproduction	Left_S_suborbital	29	1.527	0.226	0.386	-8	40	-14
Scream > Reproduction	Right_G_and_S_cingul-Mid-Post	29	1.528	0.226	0.386	7	-8	43
Scream > Reproduction	Right-Thalamus-Proper	29	1.377	0.25	0.415948276	11	-17	7
Scream > Reproduction	Right_G_insular_short	29	1.402	0.246	0.415948276	38	12	-5
Scream > Reproduction	Right-Putamen	29	1.264	0.27	0.441610169	26	2	0
Scream > Reproduction	Right_S_parieto_occipital	29	1.232	0.276	0.447630252	18	-66	28
Scream > Reproduction	Right-Pallidum	29	1.194	0.284	0.456766667	21	-4	-2
Scream > Reproduction	Right_Pole_temporal	29	1.043	0.316	0.49183871	32	9	-42
Scream > Reproduction	Right_S_front_sup	29	1.051	0.314	0.49183871	23	25	45
Scream > Reproduction	Right_S_orbital_med-olfact	29	1.061	0.312	0.49183871	12	27	-19

Scream > Reproduction	Right_S_suborbital	29	0.947	0.339	0.515173228	5	39	-18
Scream > Reproduction	Left_S_cingul-Marginalis	29	0.931	0.343	0.517179688	-13	-38	49
Scream > Reproduction	Left_G_temporal_inf	29	0.852	0.364	0.5404	-55	-40	-23
Scream > Reproduction	Left_S_front_sup	29	0.854	0.363	0.5404	-24	22	46
Scream > Reproduction	Left-Thalamus-Proper	29	0.746	0.395	0.577537879	-11	-19	7
Scream > Reproduction	Left_G_front_sup	29	0.73	0.4	0.580451128	-9	27	45
Scream > Reproduction	Left_S_orbital_lateral	29	0.708	0.407	0.586201493	-42	47	-5
Scream > Reproduction	Left-Accumbens-area	29	0.652	0.426	0.604173913	-9	11	-8
Scream > Reproduction	Right-Caudate	29	0.635	0.432	0.604173913	13	11	9
Scream > Reproduction	Left-Putamen	29	0.599	0.445	0.613464286	-26	1	0
Scream > Reproduction	Right_G_and_S_frontomargin	29	0.574	0.455	0.618415493	23	62	-11
Scream > Reproduction	Right_S_temporal_inf	29	0.556	0.462	0.619208333	55	-33	-16
Scream > Reproduction	Left_G_and_S_cingul-Mid-Post	29	0.547	0.466	0.620262069	-7	-11	41
Scream > Reproduction	Right_S_orbital_lateral	29	0.521	0.476	0.629232877	42	44	-3
Scream > Reproduction	Left-Cerebellum-White-Matter	29	0.509	0.481	0.631517007	-20	-57	-33
Scream > Reproduction	Left-Caudate	29	0.441	0.512	0.648379085	-14	10	10
Scream > Reproduction	Left_G_and_S_frontomargin	29	0.437	0.514	0.648379085	-24	61	-10
Scream > Reproduction	Right_S_cingul-Marginalis	29	0.44	0.512	0.648379085	12	-37	50
Scream > Reproduction	Right_G_rectus	29	0.424	0.52	0.651688312	4	38	-24
Scream > Reproduction	Right_G_front_middle	29	0.414	0.525	0.653709677	38	36	33
Scream > Reproduction	CC_Mid_Anterior	29	0.332	0.569	0.690672956	0	13	19
Scream > Reproduction	Left-Cerebellum-Cortex	29	0.306	0.584	0.70445	-25	-63	-37
Scream > Reproduction	Right_G_temporal_inf	29	0.298	0.59	0.706475309	55	-35	-25
Scream > Reproduction	Left_Pole_temporal	29	0.283	0.599	0.709245399	-33	6	-42
Scream > Reproduction	CC_Posterior	29	0.262	0.612	0.712704819	0	-35	17
Scream > Reproduction	CC_Mid_Posterior	29	0.264	0.611	0.712704819	0	-18	22
Scream > Reproduction	Right_S_precentral-inf-part	29	0.213	0.648	0.740023669	45	7	30
Scream > Reproduction	CC_Anterior	29	0.093	0.762	0.840377143	0	25	5
Scream > Reproduction	Left_G_rectus	29	0.094	0.762	0.840377143	-5	36	-23

Scream > Reproduction	Left_G_pariet_inf-Supramar	29	0.07	0.793	0.869596591	-60	-38	34
Scream > Reproduction	Right-choroid-plexus	29	0.043	0.837	0.910786517	20	-24	8
Scream > Reproduction	3rd-Ventricle	29	0.029	0.867	0.919896739	0	-11	-3
Scream > Reproduction	Left_S_front_middle	29	0.025	0.875	0.919896739	-29	46	19
Scream > Reproduction	Right_S_collat_transv_ant	29	0.032	0.858	0.919896739	41	-18	-29
Scream > Reproduction	Right_G_oc-temp_med-Parahip	29	0.009	0.923	0.957736559	24	-13	-29
Scream > Reproduction	Brain-Stem	29	0.004	0.948	0.969084656	0	-31	-34
Scream > Reproduction	Right-Accumbens-area	29	0.004	0.949	0.969084656	8	12	-8
Scream > Reproduction	Left_G_temp_sup-G_T_transv	29	7.06E-04	0.979	0.989251309	-49	-18	8
Scream > Reproduction	Left_S_temporal_transverse	29	0.001	0.974	0.989251309	-53	-23	5
Scream > Reproduction	Right_G_and_S_paracentral	29	3.35E-04	0.986	0.991135417	7	-35	69
Scream > Reproduction	Left_S_temporal_inf	29	5.84E-05	0.994	0.994	-55	-34	-16
Scream < Reproduction	Left_G_postcentral	29	42.036	4.27E-07	8.24E-05	-47	-25	55
Scream < Reproduction	Left_S_postcentral	29	35.215	1.91E-06	1.84E-04	-42	-33	46
Scream < Reproduction	Right_S_oc_middle_and_Lunatus	29	27.478	1.29E-05	6.22E-04	37	-79	10
Scream < Reproduction	Right_S_oc_sup_and_transversal	29	25.861	2.00E-05	7.72E-04	30	-79	23
Scream < Reproduction	Left_S_precentral-sup-part	29	24.285	3.10E-05	9.97E-04	-29	-10	56
Scream < Reproduction	Right_G_and_S_occipital_inf	29	22.589	5.04E-05	0.0012159	43	-76	-12
Scream < Reproduction	Left_G_precentral	29	19.771	1.18E-04	0.002070364	-43	-9	53
Scream < Reproduction	Left_S_occipital_ant	29	18.705	1.65E-04	0.002564143	-41	-70	7
Scream < Reproduction	Right_S_occipital_ant	29	18.328	1.86E-04	0.002564143	46	-64	5
Scream < Reproduction	Left_S_central	29	17.266	2.62E-04	0.002974471	-39	-19	46
Scream < Reproduction	Left_G_and_S_occipital_inf	29	16.007	3.99E-04	0.004278167	-39	-83	-10
Scream < Reproduction	Left_S_oc_sup_and_transversal	29	15.007	5.63E-04	0.005192619	-27	-84	21
Scream < Reproduction	Right_S_precentral-sup-part	29	14.171	7.55E-04	0.006623409	29	-9	56
Scream < Reproduction	Right_S_postcentral	29	13.882	8.38E-04	0.006859542	38	-30	49
Scream < Reproduction	Left_S_intrapariet_and_P_trans	29	12.314	0.001	0.006892857	-32	-58	43
Scream < Reproduction	Right_S_collat_transv_post	29	13.606	9.25E-04	0.006892857	26	-79	-8
Scream < Reproduction	Left_S_oc_middle_and_Lunatus	29	12.042	0.002	0.011352941	-33	-87	8

Scream < Reproduction	Right_G_occipital_middle	29	12.113	0.002	0.011352941	45	-79	15
Scream < Reproduction	Right_G_oc-temp_lat-fusifor	29	11.963	0.002	0.011352941	35	-48	-21
Scream < Reproduction	Right_S_intrapariet_and_P_trans	29	11.634	0.002	0.011352941	30	-56	47
Scream < Reproduction	Left_S_oc-temp_lat	29	8.591	0.007	0.031418605	-44	-50	-14
Scream < Reproduction	Left_S_collat_transv_post	29	7.802	0.009	0.036957447	-24	-81	-7
Scream < Reproduction	Right_G_postcentral	29	7.868	0.009	0.036957447	47	-21	54
Scream < Reproduction	Right_S_oc-temp_lat	29	6.613	0.016	0.055142857	45	-47	-15
Scream < Reproduction	Left_G_occipital_middle	29	5.699	0.024	0.07352381	-39	-88	12
Scream < Reproduction	Left_G_parietal_sup	29	5.401	0.027	0.078954545	-26	-61	59
Scream < Reproduction	Right_S_oc-temp_med_and_Lingual	29	4.927	0.034	0.089890411	28	-48	-11
Scream < Reproduction	Right_G_occipital_sup	29	4.429	0.044	0.111736842	20	-89	31
Scream < Reproduction	Right_G_precentral	29	4.397	0.045	0.112792208	42	-7	53
Scream < Reproduction	Right_Pole_occipital	29	4.248	0.048	0.117265823	20	-95	-3
Scream < Reproduction	Left_G_oc-temp_lat-fusifor	29	3.347	0.078	0.165428571	-36	-55	-19
Scream < Reproduction	Right_G_parietal_sup	29	3.205	0.084	0.174322581	25	-60	62
Scream < Reproduction	Left_G_occipital_sup	29	2.907	0.099	0.19903125	-15	-92	29
Scream < Reproduction	Left_Pole_occipital	29	2.757	0.108	0.212693878	-16	-101	-4
Scream < Reproduction	Left_G_and_S_subcentral	29	1.718	0.2	0.359194444	-57	-11	15
Scream < Reproduction	Left_S_circular_insula_sup	29	1.379	0.25	0.415948276	-36	6	11
Scream < Reproduction	Right_S_calcarine	29	1.108	0.301	0.480107438	18	-65	7
Scream < Reproduction	WM-hypointensities	29	1.014	0.322	0.497168	-4	-14	19
Scream < Reproduction	Right_G_oc-temp_med-Lingual	29	0.997	0.326	0.499349206	10	-67	-4
Scream < Reproduction	Right-Cerebellum-Cortex	29	0.781	0.384	0.565740458	24	-63	-37
Scream < Reproduction	Right-Lateral-Ventricle	29	0.635	0.432	0.604173913	14	-14	14
Scream < Reproduction	Left_G_oc-temp_med-Lingual	29	0.635	0.432	0.604173913	-10	-73	-6
Scream < Reproduction	Right-Cerebellum-White-Matter	29	0.618	0.438	0.608158273	19	-56	-33
Scream < Reproduction	Right_S_circular_insula_sup	29	0.575	0.454	0.618415493	37	9	9
Scream < Reproduction	Right_G_and_S_cingul-Mid-Ant	29	0.561	0.46	0.619208333	7	17	35
Scream < Reproduction	Right_S_central	29	0.493	0.488	0.636378378	37	-17	47

Scream < Reproduction	Left_S_precentral-inf-part	29	0.443	0.511	0.648379085	-45	4	30
Scream < Reproduction	Right_S_temporal_transverse	29	0.446	0.509	0.648379085	53	-18	7
Scream < Reproduction	Left_S_calcarine	29	0.394	0.535	0.661891026	-15	-69	5
Scream < Reproduction	4th-Ventricle	29	0.38	0.543	0.667509554	-1	-46	-35
Scream < Reproduction	Right-Cerebral-White-Matter	29	0.349	0.559	0.682829114	29	-18	19
Scream < Reproduction	Left_Lat_Fis-post	29	0.292	0.593	0.706475309	-41	-34	20
Scream < Reproduction	Left_G_and_S_paracentral	29	0.262	0.613	0.712704819	-8	-36	68
Scream < Reproduction	Left-Cerebral-White-Matter	29	0.232	0.634	0.732706587	-29	-19	19
Scream < Reproduction	Left_S_oc-temp_med_and_Lingual	29	0.217	0.645	0.740023669	-28	-48	-11
Scream < Reproduction	Right_S_front_middle	29	0.197	0.661	0.750429412	30	47	19
Scream < Reproduction	Left_G_and_S_cingul-Mid-Ant	29	0.179	0.675	0.761842105	-7	16	35
Scream < Reproduction	Right_G_cuneus	29	0.165	0.687	0.770877907	7	-80	21
Scream < Reproduction	Left-Lateral-Ventricle	29	0.161	0.691	0.770884393	-15	-17	14
Scream < Reproduction	Right_G_temp_sup-G_T_transv	29	0.042	0.84	0.910786517	48	-14	7
Scream < Reproduction	Left-choroid-plexus	29	0.037	0.85	0.916480447	-22	-31	7
Scream < Reproduction	Right_G_pariet_inf-Supramar	29	0.028	0.869	0.919896739	59	-29	36
Scream < Reproduction	Right_Lat_Fis-post	29	0.024	0.877	0.919896739	42	-27	20
Scream < Reproduction	Right_G_and_S_subcentral	29	0.013	0.911	0.950394595	57	-8	14
Scream < Reproduction	Left_G_cuneus	29	0.006	0.938	0.968096257	-4	-84	19

Table S10 – Brain-behavior analysis: ROIs on accuracy of reproduction, based on phase (encoding, reproduction) and condition (safe/threat). For each of 15 a priori ROIs, comprising salience regions (amygdala, insular subregions, cingulate subregions) and the sensorimotor and parietal regions implicated in our phase contrast (precentral gyrus, postcentral gyrus, central sulcus, paracentral lobule, superior parietal gyrus, intraparietal sulcus), we correlated subject-level activation during each phase (encoding, reproduction, and scream) with mean AR in the corresponding condition. Across 75 tests, five correlations emerged as significant uncorrected, but none survived FDR correction applied across the full family (p_FDR ≥ .26).

ROI	Condition	Regressor	n	r	CI_low	CI_high	p-value	p-value (FDR-corr)
G_parietal_sup	threat	Scream	29	-0.524	-0.747	-0.195	0.0035	0.262
S_intrapariet_and_P_trans	threat	Scream	29	-0.464	-0.71	-0.118	0.0111	0.32
G_and_S_paracentral	threat	Scream	29	-0.456	-0.705	-0.108	0.0128	0.32
G_precentral	threat	Scream	29	-0.392	-0.663	-0.029	0.0356	0.609
G_and_S_cingul-Ant	threat	Reproduction	29	0.374	0.009	0.651	0.0456	0.609
G_Ins_lg_and_S_cent_ins	threat	Reproduction	29	0.354	-0.014	0.638	0.0594	0.609
G_and_S_cingul-Ant	threat	Encoding	29	0.351	-0.018	0.635	0.0623	0.609
G_and_S_paracentral	threat	Reproduction	29	0.347	-0.022	0.633	0.065	0.609
S_circular_insula_sup	threat	Scream	29	-0.325	-0.618	0.047	0.0855	0.639
S_circular_insula_ant	threat	Reproduction	29	0.312	-0.061	0.609	0.0988	0.639
G_parietal_sup	threat	Reproduction	29	0.306	-0.068	0.605	0.1063	0.639
S_central	threat	Scream	29	-0.3	-0.601	0.074	0.1135	0.639
G_cingul-Post-ventral	threat	Reproduction	29	0.289	-0.086	0.593	0.1278	0.639
G_cingul-Post-ventral	threat	Scream	29	-0.289	-0.592	0.087	0.1289	0.639
G_postcentral	threat	Scream	29	-0.28	-0.586	0.097	0.1415	0.639
S_circular_insula_inf	threat	Reproduction	29	0.275	-0.102	0.583	0.1487	0.639
G_Ins_lg_and_S_cent_ins	threat	Encoding	29	0.264	-0.114	0.575	0.1668	0.639
G_postcentral	threat	Reproduction	29	0.262	-0.116	0.573	0.17	0.639
G_postcentral	threat	Encoding	29	0.261	-0.116	0.573	0.1711	0.639
G_and_S_paracentral	threat	Encoding	29	0.259	-0.118	0.572	0.1741	0.639
G_and_S_cingul-Mid-Ant	threat	Scream	29	-0.253	-0.567	0.125	0.185	0.639
G_insular_short	threat	Scream	29	-0.249	-0.564	0.129	0.1924	0.639

S_circular_insula_ant	threat	Encoding	29	0.247	-0.131	0.563	0.1961	0.639
G_and_S_cingul-Mid-Ant	threat	Reproduction	29	0.24	-0.139	0.558	0.2095	0.655
G_insular_short	threat	Reproduction	29	0.226	-0.154	0.547	0.2392	0.718
G_and_S_cingul-Mid-Ant	threat	Encoding	29	0.214	-0.165	0.539	0.2642	0.744
G_parietal_sup	threat	Encoding	29	0.212	-0.167	0.537	0.2693	0.744
S_circular_insula_inf	threat	Scream	29	-0.208	-0.534	0.171	0.2779	0.744
G_cingul-Post-ventral	threat	Encoding	29	0.199	-0.18	0.527	0.2996	0.752
Amygdala	threat	Scream	29	-0.195	-0.524	0.185	0.3104	0.752
G_insular_short	threat	Encoding	29	0.194	-0.185	0.523	0.3127	0.752
G_Ins_lg_and_S_cent_ins	threat	Scream	29	-0.191	-0.521	0.189	0.3221	0.752
G_parietal_sup	safe	Reproduction	29	0.187	-0.193	0.518	0.3309	0.752
S_circular_insula_sup	threat	Reproduction	29	0.178	-0.201	0.511	0.3549	0.771
G_cingul-Post-ventral	safe	Reproduction	29	0.176	-0.203	0.51	0.3597	0.771
S_circular_insula_ant	safe	Encoding	29	0.172	-0.207	0.507	0.371	0.773
S_circular_insula_inf	threat	Encoding	29	0.162	-0.217	0.499	0.4012	0.794
S_circular_insula_ant	threat	Scream	29	-0.162	-0.499	0.218	0.4021	0.794
S_circular_insula_ant	safe	Reproduction	29	0.145	-0.234	0.485	0.4543	0.86
G_parietal_sup	safe	Encoding	29	0.141	-0.238	0.483	0.4658	0.86
S_circular_insula_sup	threat	Encoding	29	0.14	-0.239	0.481	0.4704	0.86
G_cingul-Post-ventral	safe	Encoding	29	0.135	-0.244	0.478	0.4861	0.868
Amygdala	threat	Reproduction	29	0.118	-0.259	0.465	0.5404	0.943
G_and_S_cingul-Ant	safe	Reproduction	29	0.112	-0.265	0.46	0.5613	0.955
G_precentral	threat	Reproduction	29	0.109	-0.268	0.457	0.5732	0.955
S_circular_insula_inf	safe	Reproduction	29	0.105	-0.272	0.454	0.5893	0.955
S_circular_insula_inf	safe	Encoding	29	0.102	-0.275	0.452	0.5986	0.955
G_precentral	threat	Encoding	29	0.099	-0.278	0.449	0.6109	0.955
G_and_S_cingul-Ant	safe	Encoding	29	0.095	-0.281	0.446	0.6239	0.955
G_postcentral	safe	Reproduction	29	0.086	-0.289	0.439	0.6561	0.966
G_Ins_lg_and_S_cent_ins	safe	Reproduction	29	0.078	-0.297	0.432	0.6881	0.966
G_postcentral	safe	Encoding	29	0.075	-0.3	0.43	0.6998	0.966
G_and_S_cingul-Ant	threat	Scream	29	-0.07	-0.426	0.304	0.7186	0.966

Amygdala	safe	Reproduction	29	0.068	-0.306	0.424	0.7271	0.966
S_central	threat	Encoding	29	0.067	-0.307	0.423	0.7315	0.966
G_Ins_lg_and_S_cent_ins	safe	Encoding	29	0.065	-0.309	0.421	0.7386	0.966
S_central	threat	Reproduction	29	0.064	-0.31	0.421	0.741	0.966
S_intrapariet_and_P_trans	safe	Reproduction	29	0.061	-0.313	0.418	0.7544	0.966
G_insular_short	safe	Encoding	29	0.049	-0.323	0.408	0.8003	0.966
G_precentral	safe	Encoding	29	0.045	-0.327	0.405	0.8173	0.966
G_and_S_cingul-Mid-Ant	safe	Encoding	29	0.043	-0.328	0.403	0.8235	0.966
G_and_S_paracentral	safe	Encoding	29	-0.042	-0.403	0.329	0.8269	0.966
S_intrapariet_and_P_trans	safe	Encoding	29	0.04	-0.331	0.401	0.8353	0.966
S_intrapariet_and_P_trans	threat	Encoding	29	-0.04	-0.401	0.331	0.8369	0.966
S_circular_insula_sup	safe	Reproduction	29	-0.039	-0.4	0.332	0.841	0.966
Amygdala	safe	Encoding	29	0.035	-0.336	0.396	0.8578	0.966
Amygdala	threat	Encoding	29	-0.032	-0.394	0.339	0.8693	0.966
G_and_S_cingul-Mid-Ant	safe	Reproduction	29	0.03	-0.34	0.392	0.8758	0.966
G_precentral	safe	Reproduction	29	0.023	-0.346	0.386	0.9047	0.98
S_central	safe	Encoding	29	-0.015	-0.379	0.354	0.939	0.98
G_insular_short	safe	Reproduction	29	0.013	-0.355	0.378	0.9462	0.98
S_circular_insula_sup	safe	Encoding	29	-0.013	-0.377	0.356	0.9485	0.98
G_and_S_paracentral	safe	Reproduction	29	-0.01	-0.375	0.357	0.9572	0.98
S_intrapariet_and_P_trans	threat	Reproduction	29	0.008	-0.359	0.373	0.9668	0.98
S_central	safe	Reproduction	29	0.003	-0.364	0.369	0.9878	0.988

Table S11 – Brain-behavior analysis. For each of 15 a priori ROIs, we fitted models testing whether neural response moderates the within-subject relationship between subjective anxiety and accuracy of reproduction (anxiety × ROI), and the full three-way interaction additionally including interoceptive accuracy (anxiety x interoception x ROI) (75 models per specification). No interaction reached significance (two-way: $p \geq .27$, three-way: $p \geq .12$).

TWO-WAY: Subjective Anxiety x mean beta ROIs on AR

Condition	ROI	Regressor	b	t-value	df	p-value	p-value (FDR-corr)
threat	G_and_S_cingul-Mid-Ant	Encoding	-0.0647	-1.1	989	0.2704	0.997
safe	G_and_S_cingul-Ant	Encoding	0.0726	0.98	435	0.3262	0.997
safe	G_and_S_cingul-Ant	Reproduction	0.0663	0.91	481	0.3627	0.997
threat	G_cingul-Post-ventral	Encoding	-0.0387	-0.88	1282	0.38	0.997
threat	G_and_S_cingul-Ant	Scream	0.0468	0.83	983	0.4074	0.997
threat	G_precentral	Scream	0.053	0.81	682	0.4162	0.997
threat	G_postcentral	Scream	0.044	0.8	968	0.4255	0.997
threat	G_and_S_cingul-Mid-Ant	Reproduction	-0.047	-0.78	1055	0.4349	0.997
safe	G_insular_short	Encoding	0.0622	0.75	618	0.4534	0.997
threat	G_precentral	Encoding	-0.0542	-0.73	743	0.4656	0.997
safe	G_insular_short	Reproduction	0.0619	0.72	603	0.4715	0.997
threat	S_central	Scream	0.035	0.6	1087	0.5458	0.997
threat	S_circular_insula_inf	Encoding	-0.0299	-0.55	769	0.5809	0.997
threat	Amygdala	Reproduction	0.0283	0.55	948	0.5819	0.997
threat	S_circular_insula_sup	Encoding	-0.0422	-0.55	710	0.5823	0.997
safe	S_circular_insula_ant	Encoding	0.0388	0.54	534	0.5898	0.997
threat	G_and_S_cingul-Mid-Ant	Scream	0.0298	0.54	882	0.5921	0.997
threat	G_precentral	Reproduction	-0.0377	-0.51	824	0.6097	0.997
threat	G_parietal_sup	Encoding	-0.0258	-0.49	952	0.6221	0.997
safe	S_circular_insula_ant	Reproduction	0.0365	0.49	543	0.6231	0.997
safe	G_Ins_lg_and_S_cent_ins	Reproduction	-0.0323	-0.44	1015	0.6631	0.997
threat	S_intrapariet_and_P_trans	Scream	-0.0234	-0.44	664	0.6636	0.997

threat	G_postcentral	Encoding	-0.0217	-0.43	848	0.6671	0.997
safe	G_postcentral	Encoding	0.0196	0.41	835	0.6855	0.997
threat	G_parietal_sup	Reproduction	-0.0217	-0.4	948	0.686	0.997
safe	G_postcentral	Reproduction	0.0195	0.4	850	0.6877	0.997
threat	G_insular_short	Scream	0.0313	0.4	1230	0.6921	0.997
threat	Amygdala	Scream	-0.022	-0.39	1216	0.6954	0.997
threat	G_postcentral	Reproduction	-0.0202	-0.39	855	0.6996	0.997
threat	G_cingul-Post-ventral	Scream	0.009	0.36	1004	0.7159	0.997
threat	S_circular_insula_ant	Reproduction	0.0169	0.36	863	0.7176	0.997
safe	G_cingul-Post-ventral	Reproduction	-0.0166	-0.35	459	0.7234	0.997
safe	G_Ins_lg_and_S_cent_ins	Encoding	-0.0255	-0.35	1015	0.7247	0.997
threat	S_central	Encoding	-0.0234	-0.34	700	0.7318	0.997
threat	S_circular_insula_sup	Reproduction	-0.0257	-0.34	858	0.7363	0.997
threat	S_central	Reproduction	-0.0223	-0.33	694	0.743	0.997
threat	G_Ins_lg_and_S_cent_ins	Scream	0.0182	0.31	1292	0.7549	0.997
threat	G_cingul-Post-ventral	Reproduction	-0.0123	-0.3	1211	0.762	0.997
safe	S_central	Reproduction	0.0146	0.28	1173	0.7809	0.997
safe	G_and_S_cingul-Mid-Ant	Encoding	0.0204	0.27	279	0.7866	0.997
threat	G_and_S_cingul-Ant	Reproduction	0.0159	0.27	1042	0.7869	0.997
safe	S_central	Encoding	0.0138	0.27	1193	0.7906	0.997
threat	S_circular_insula_sup	Scream	0.0176	0.26	1125	0.7969	0.997
threat	G_Ins_lg_and_S_cent_ins	Encoding	-0.018	-0.24	609	0.8068	0.997
threat	S_intrapariet_and_P_trans	Encoding	0.0114	0.24	1084	0.8073	0.997
threat	G_insular_short	Encoding	-0.0154	-0.24	662	0.8075	0.997
safe	G_cingul-Post-ventral	Encoding	-0.0111	-0.24	421	0.8132	0.997
safe	G_and_S_cingul-Mid-Ant	Reproduction	0.018	0.23	274	0.8207	0.997
safe	G_parietal_sup	Encoding	0.0146	0.21	695	0.8313	0.997
safe	G_and_S_paracentral	Encoding	0.0158	0.2	822	0.8393	0.997
threat	S_circular_insula_inf	Scream	-0.0105	-0.2	1288	0.842	0.997
threat	G_and_S_paracentral	Reproduction	0.0121	0.19	691	0.8466	0.997
safe	Amygdala	Encoding	0.0095	0.19	763	0.8498	0.997

threat	G_Ins_lg_and_S_cent_ins	Reproduction	0.0118	0.17	744	0.8634	0.997
threat	Amygdala	Encoding	0.0092	0.16	814	0.8733	0.997
threat	S_circular_insula_ant	Scream	0.0093	0.13	1136	0.8979	0.997
threat	G_and_S_paracentral	Scream	-0.0082	-0.13	680	0.8989	0.997
safe	G_precentral	Encoding	0.0085	0.12	689	0.902	0.997
safe	G_parietal_sup	Reproduction	0.0079	0.11	766	0.9094	0.997
safe	G_and_S_paracentral	Reproduction	0.0081	0.11	871	0.9145	0.997
safe	G_precentral	Reproduction	0.007	0.1	728	0.9211	0.997
threat	G_parietal_sup	Scream	0.0055	0.09	522	0.9244	0.997
safe	S_circular_insula_inf	Encoding	0.0055	0.09	526	0.9266	0.997
safe	S_intrapariet_and_P_trans	Encoding	0.0074	0.08	739	0.9372	0.997
threat	G_and_S_paracentral	Encoding	-0.0045	-0.07	730	0.9434	0.997
safe	S_circular_insula_sup	Reproduction	-0.0061	-0.07	664	0.9459	0.997
safe	S_circular_insula_sup	Encoding	-0.0048	-0.06	683	0.9554	0.997
threat	S_intrapariet_and_P_trans	Reproduction	0.0026	0.05	1110	0.9589	0.997
threat	S_circular_insula_inf	Reproduction	0.001	0.02	1029	0.9835	0.997
safe	Amygdala	Reproduction	0.001	0.02	723	0.9843	0.997
threat	S_circular_insula_ant	Encoding	0.0008	0.02	786	0.9864	0.997
safe	S_intrapariet_and_P_trans	Reproduction	0.0015	0.02	792	0.9874	0.997
safe	S_circular_insula_inf	Reproduction	0.0008	0.01	548	0.9896	0.997
threat	G_and_S_cingul-Ant	Encoding	-0.0005	-0.01	1076	0.9939	0.997
threat	G_insular_short	Reproduction	0.0002	0	724	0.9973	0.997

THREE-WAY: Subjective Anxiety x Interoceptive Accuracy x mean beta ROIs on AR

Condition	ROI	Regressor	b	t-value	df	p-value	p-value (FDR-corr)
threat	G_and_S_cingul-Ant	Encoding	0.1443	1.54	378	0.1254	0.916
threat	Amygdala	Reproduction	-0.1114	-1.43	543	0.1536	0.916
threat	G_and_S_cingul-Mid-Ant	Encoding	0.1185	1.42	1127	0.1548	0.916

threat	G_and_S_cingul-Mid-Ant	Reproduction	0.1242	1.4	1060	0.1612	0.916
threat	S_circular_insula_inf	Reproduction	0.0981	1.3	894	0.1944	0.916
threat	Amygdala	Encoding	-0.0926	-1.26	567	0.2083	0.916
threat	G_postcentral	Encoding	0.1069	1.25	445	0.2107	0.916
threat	S_circular_insula_ant	Scream	0.1132	1.24	601	0.2138	0.916
threat	G_cingul-Post-ventral	Encoding	0.0747	1.2	722	0.2319	0.916
threat	S_circular_insula_inf	Encoding	0.0811	1.2	1070	0.232	0.916
threat	G_postcentral	Reproduction	0.1116	1.19	463	0.2356	0.916
threat	G_and_S_cingul-Ant	Reproduction	0.1078	1.16	629	0.246	0.916
threat	G_parietal_sup	Reproduction	0.1024	1.16	810	0.2476	0.916
threat	G_parietal_sup	Encoding	0.0935	1.13	991	0.2605	0.916
threat	G_Ins_lg_and_S_cent_ins	Reproduction	0.1257	1.08	326	0.2829	0.916
threat	G_Ins_lg_and_S_cent_ins	Encoding	0.1138	1.07	501	0.2867	0.916
threat	G_precentral	Scream	-0.0897	-1.04	236	0.2974	0.916
threat	Amygdala	Scream	-0.1204	-1.02	589	0.3074	0.916
threat	G_cingul-Post-ventral	Reproduction	0.0449	0.99	983	0.3208	0.916
threat	S_central	Scream	-0.0977	-0.97	650	0.3333	0.916
threat	G_precentral	Encoding	0.0929	0.89	594	0.3732	0.916
threat	G_postcentral	Scream	-0.0592	-0.88	904	0.3809	0.916
threat	S_circular_insula_sup	Scream	-0.0895	-0.86	444	0.39	0.916
threat	G_precentral	Reproduction	0.0859	0.82	596	0.4103	0.916
threat	S_circular_insula_sup	Reproduction	0.088	0.81	1119	0.416	0.916
threat	S_circular_insula_sup	Encoding	0.0838	0.8	1168	0.4258	0.916
threat	G_insular_short	Encoding	0.0653	0.78	1010	0.4368	0.916
safe	G_Ins_lg_and_S_cent_ins	Reproduction	0.0671	0.75	346	0.4513	0.916
threat	S_central	Encoding	0.0797	0.75	419	0.4542	0.916
safe	G_Ins_lg_and_S_cent_ins	Encoding	0.0618	0.74	353	0.4613	0.916
threat	G_and_S_cingul-Mid-Ant	Scream	-0.0461	-0.67	447	0.5011	0.916
threat	G_insular_short	Reproduction	0.0568	0.63	936	0.5288	0.916
threat	G_and_S_cingul-Ant	Scream	0.0457	0.63	706	0.5299	0.916
threat	S_central	Reproduction	0.0611	0.59	456	0.5568	0.916

safe	G_precentral	Encoding	0.0406	0.58	292	0.5614	0.916
safe	S_circular_insula_inf	Encoding	0.0374	0.57	326	0.5664	0.916
threat	S_circular_insula_ant	Encoding	0.0374	0.57	714	0.5698	0.916
threat	G_Ins_lg_and_S_cent_ins	Scream	-0.0731	-0.56	491	0.5732	0.916
threat	G_and_S_paracentral	Scream	-0.0314	-0.55	386	0.5828	0.916
safe	G_precentral	Reproduction	0.0396	0.54	290	0.5906	0.916
safe	S_circular_insula_inf	Reproduction	0.0351	0.52	320	0.6052	0.916
safe	S_circular_insula_sup	Reproduction	0.0492	0.51	548	0.6091	0.916
threat	S_circular_insula_ant	Reproduction	0.0366	0.51	660	0.6124	0.916
threat	G_cingul-Post-ventral	Scream	-0.0172	-0.51	632	0.6137	0.916
safe	S_circular_insula_sup	Encoding	0.0434	0.46	509	0.6424	0.916
safe	G_and_S_cingul-Ant	Encoding	-0.0478	-0.45	172	0.6511	0.916
safe	G_and_S_cingul-Ant	Reproduction	-0.0415	-0.41	200	0.6828	0.916
threat	S_intrapariet_and_P_trans	Reproduction	0.0259	0.36	1133	0.7221	0.916
safe	Amygdala	Reproduction	0.0247	0.35	408	0.7269	0.916
threat	G_parietal_sup	Scream	-0.0207	-0.34	320	0.7353	0.916
safe	S_circular_insula_ant	Reproduction	0.0286	0.33	262	0.7408	0.916
safe	G_and_S_cingul-Mid-Ant	Reproduction	0.0295	0.32	227	0.7462	0.916
safe	S_circular_insula_ant	Encoding	0.0254	0.3	281	0.7663	0.916
safe	G_postcentral	Reproduction	0.0181	0.28	274	0.7807	0.916
safe	S_intrapariet_and_P_trans	Reproduction	0.031	0.28	594	0.7825	0.916
safe	G_postcentral	Encoding	0.0161	0.26	285	0.7936	0.916
safe	G_and_S_paracentral	Reproduction	0.0228	0.24	438	0.809	0.916
threat	S_intrapariet_and_P_trans	Encoding	0.0162	0.24	1163	0.8129	0.916
safe	S_central	Encoding	0.0174	0.23	477	0.8171	0.916
safe	S_central	Reproduction	0.0178	0.23	460	0.8176	0.916
safe	G_cingul-Post-ventral	Encoding	-0.0141	-0.22	222	0.8232	0.916
safe	G_and_S_cingul-Mid-Ant	Encoding	0.0185	0.22	211	0.8283	0.916
threat	S_circular_insula_inf	Scream	0.0161	0.22	1113	0.8297	0.916
threat	G_insular_short	Scream	0.0212	0.21	726	0.8326	0.916
safe	Amygdala	Encoding	0.0152	0.21	425	0.8369	0.916

threat	G_and_S_paracentral	Encoding	-0.018	-0.19	476	0.8459	0.916
safe	G_parietal_sup	Encoding	-0.0163	-0.19	383	0.8478	0.916
safe	G_and_S_paracentral	Encoding	0.0191	0.19	439	0.8496	0.916
safe	G_cingul-Post-ventral	Reproduction	-0.0105	-0.18	280	0.8569	0.916
safe	S_intrapariet_and_P_trans	Encoding	0.0193	0.17	493	0.8617	0.916
threat	S_intrapariet_and_P_trans	Scream	0.0094	0.17	523	0.8684	0.916
safe	G_parietal_sup	Reproduction	-0.0131	-0.15	388	0.8795	0.916
threat	G_and_S_paracentral	Reproduction	-0.0128	-0.13	353	0.8948	0.918
safe	G_insular_short	Reproduction	-0.011	-0.12	294	0.9059	0.918
safe	G_insular_short	Encoding	-0.0073	-0.08	288	0.9356	0.936
